# Hydrolysis of 5-methylfuran-2-yl to 2,5-dioxopentanyl allows for stable bio-orthogonal proximity-induced ligation

**DOI:** 10.1038/s42004-021-00584-1

**Published:** 2021-10-22

**Authors:** Alex Manicardi, Enrico Cadoni, Annemieke Madder

**Affiliations:** Organic and Biomimetic Chemistry Research Group, Department of Organic and Macromolecular Chemistry, Ghent University, Krijgslaan 281-S4, 9000 Ghent, Belgium

**Keywords:** Synthetic chemistry methodology, Self-assembly

## Abstract

Ligation methodologies featuring bio-orthogonal units and leading to the formation of a stable adduct are the ideal candidates for being applied in a biological context. However, most of the available strategies rely on highly reactive species that require careful handling, or on the activation of pro-reactive functional groups. We here report on a proximity-induced ligation reaction that relies on a stable 2,5-dione, that can be conveniently generated under acidic conditions from a 2,5-dialkylfuran building block, and hydrazine nucleophiles. This bio-orthogonal ligation, which proceeds under physiological conditions, does not require any stimulus or trigger and leads to the formation of a pyridazinium adduct that demonstrates excellent stability under harsh conditions (24 h at 90 °C). The reaction was applied to the formation of PNA-PNA adducts, DNA- and RNA-templated ligations, and for the formation of peptide-peptide adducts in solution. This convenient methodology was further implemented on plastic and glass surfaces to realize self-addressable covalent constructs.

## Abstract (web summary)

Proximity-based ligations commonly require an external stimulus such as a catalyst or irradiation, or highly reactive functional groups. Here the reaction of alpha effect nucleophiles and 2,5-dioxopentanyl derivatives allows direct proximity-based ligation while avoiding highly reactive moieties.

## Introduction

Small complementary chemical functionalities that allow for selective and specific formation of a stable product are ideal candidates for the development of new ligation methodologies^1,2^. If these functionalities, and thus the resulting reaction between them, are bio-orthogonal, the new ligation has the potential to be applied to biologically relevant molecules or environments^3^. Recently, scenarios where proximity is used as the sole prerequisite for the envisaged ligation to occur, have gained considerable interest in view of the high selectivity that can be guaranteed under such conditions for specific ligation between selected partners in complex mixtures. The lack of addition of any chemical trigger renders such reactions ideally suited for the on-demand connection between two highly functionalized and sensitive moieties^4–7^. Through the years, different chemistries for bio-orthogonal ligation have been reported, some of which have found applications in such proximity-induced ligations, including, among others, nucleophilic substitutions^6–8^, native chemical ligations^9–11^, cycloadditions^12–16^, Michael additions^17^, sulfur-fluoride exchange reactions^18^, and, by extension, photochemical reactions^19–23^. However, the number of available chemistries remains limited and, often, the required functional groups suffer from reduced stability upon prolonged exposure to biologically relevant conditions, thus limiting their shelf-life if special precautions are not taken. In particular, thiols and phosphines can be easily oxidized by dissolved oxygen, while reactive electrophiles (e.g., thio- and seleno-esters, sulfur halides) and strained alkenes and alkynes are susceptible to hydrolysis in aqueous environments or might cross-react with biological components. In addition, some of the required functionalities are not easily synthetically accessible (e.g., strained alkynes, tetrazines).

Formation of oximes and hydrazones stands out due to the simplicity of the functional groups involved and their stability^24^. Unfortunately, both reactions fail to find solid ground in proximity-induced applications for the reversible nature of the bonds formed, although in recent years remarkable progress has been made towards irreversible alternatives, that, in some cases, require bulkier moieties^25–29^.

**Fig. 1:**
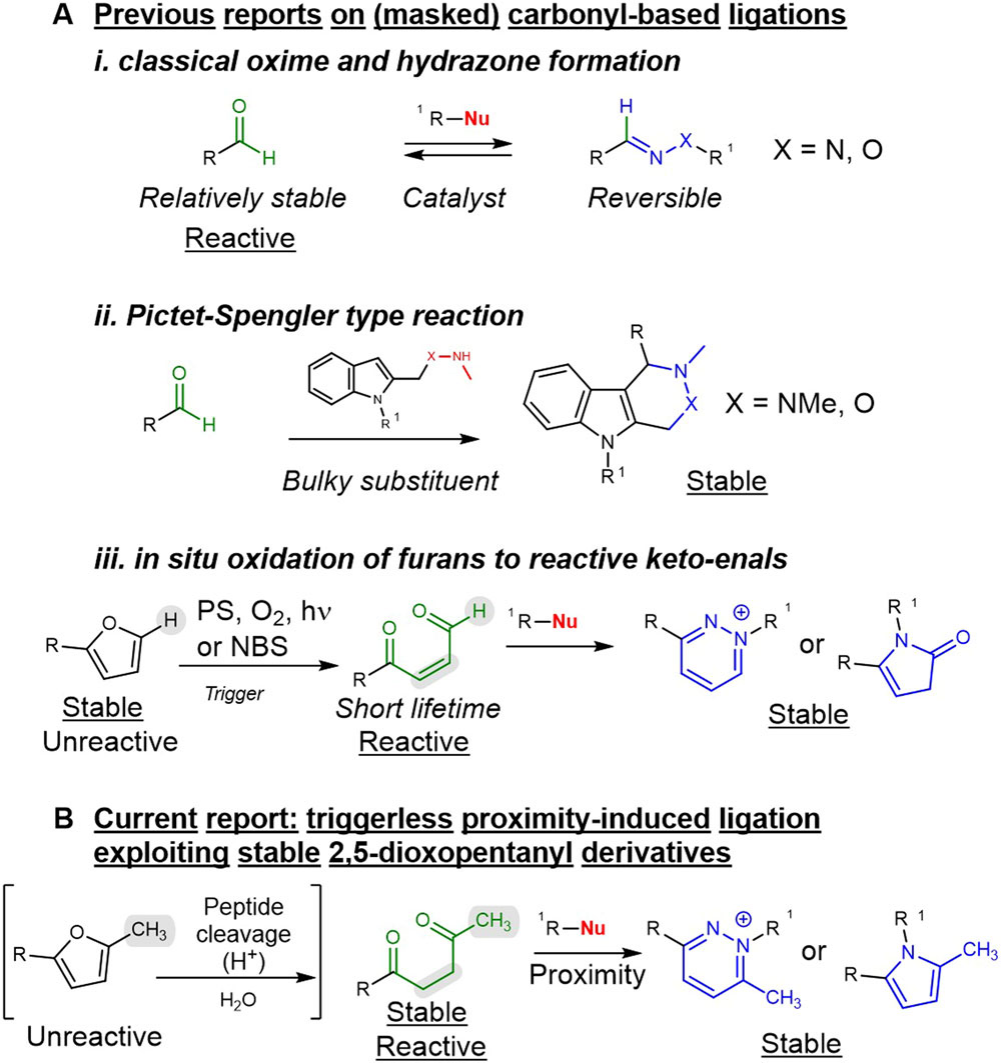
Comparison between previously reported carbonyl-based biorthogonal ligations and the triggerless proximity-induced ligation reported in this work. **A** previous reports: (i) reversible oxime and hydrazone formation; (ii) irreversible Pictet-Spengler variation exploiting bulky indoles; (iii) furan-based oxidation-induced ligation. **B** DOP-ligation. PS = photosensitizer; NBS: N-bromosuccinimide.

In this work, we report on a ligation reaction where the only requirement for the reaction is the proximity between two, otherwise stable, functional groups, which leads to the formation of a stable product under biologically relevant conditions (Fig. 1). The reaction, initially applied to peptide nucleic acids (PNAs) for PNA-PNA ligation, was then extended to oligonucleotide templated reactions, peptide-peptide ligation, and was demonstrated to work both in solution (including in cell lysate) and on plastic and glass surfaces.

## Results and discussion

Recently, we reported on the incorporation of a furan-2-yl moiety and α-effect nucleophiles into peptides for the realization of a light-triggered peptide-peptide ligation^30^. This ligation protocol relies on the in situ oxidative conversion of the furan moiety into a highly reactive, short-lived, keto-enal functionality susceptible to nucleophilic attack. This approach was later exploited for the ‘on surface’ ligation of complementary PNAs, where the induced proximity allowed the use of standard amines as suitable nucleophiles^20^. In this context, 5-methylfuran-modified PNA sequences were foreseen as potential negative controls. We surmised that oxidation of these derivatives would lead to the in-situ formation of a less reactive keto-enone. Unfortunately, the insertion of this functionality on solid support failed to provide the desired 5-methylfuran-2-yl-containing probe. Indeed, during the final cleavage step, required to release the PNA from the resin (10% m-cresol in trifluoroacetic acid, TFA, 1 h), an acid-catalyzed retro-Paal-Knorr reaction led to the clean and complete hydrolysis of the aromatic ring to the corresponding 2,5-dioxopentanyl (2,5-DOP) derivative (see Supplementary Information for examples of crude HPLCs, Supplementary Fig. 3), although this kind of reaction is generally reported under harsher conditions (i.e. stronger acids, high temperatures, longer times). As this reaction was never observed for furan-2-yl derivatives, we reasoned that the extra carbocation-stabilizing effect introduced by the presence of the 5-alkyl modification (i.e., methyl) on the furan ring allowed this efficient hydrolysis during the TFA cleavage.

Given this unexpected, but very convenient and clean conversion of the 5-methylfuran-2-yl moiety into the stable 2,5-DOP entity, and intrigued by the nature of this product, we explored the possibility to exploit this functional group as a stable handle for the post-cleavage modification of the PNA probes.

When the reactivity of this system was tested in presence of different nucleophiles, under conditions normally exploited for the generation of pyrazoles from 1,3-diketones^28,31^, no significant modification of the dicarbonylic system could be observed. However, when 2,5-DOP-containing PNA probes were hybridized in presence of fully matching hydrazine-containing PNA probes, proximity-induced reaction between the DOP and nucleophilic moiety was observed to proceed with excellent conversion resulting in a stable adduct (vide infra).

Intrigued by the unique and proximity-induced nature of the observed process, we decided to further investigate the full scope and limitations of this ligation by exploiting different scenarios for induction of proximity between biomolecules including, nucleic acid hybridization, templated recognition between nucleic acid probes and formation of coiled-coil peptide dimers.

### PNA-PNA ligation

**Fig. 2:**
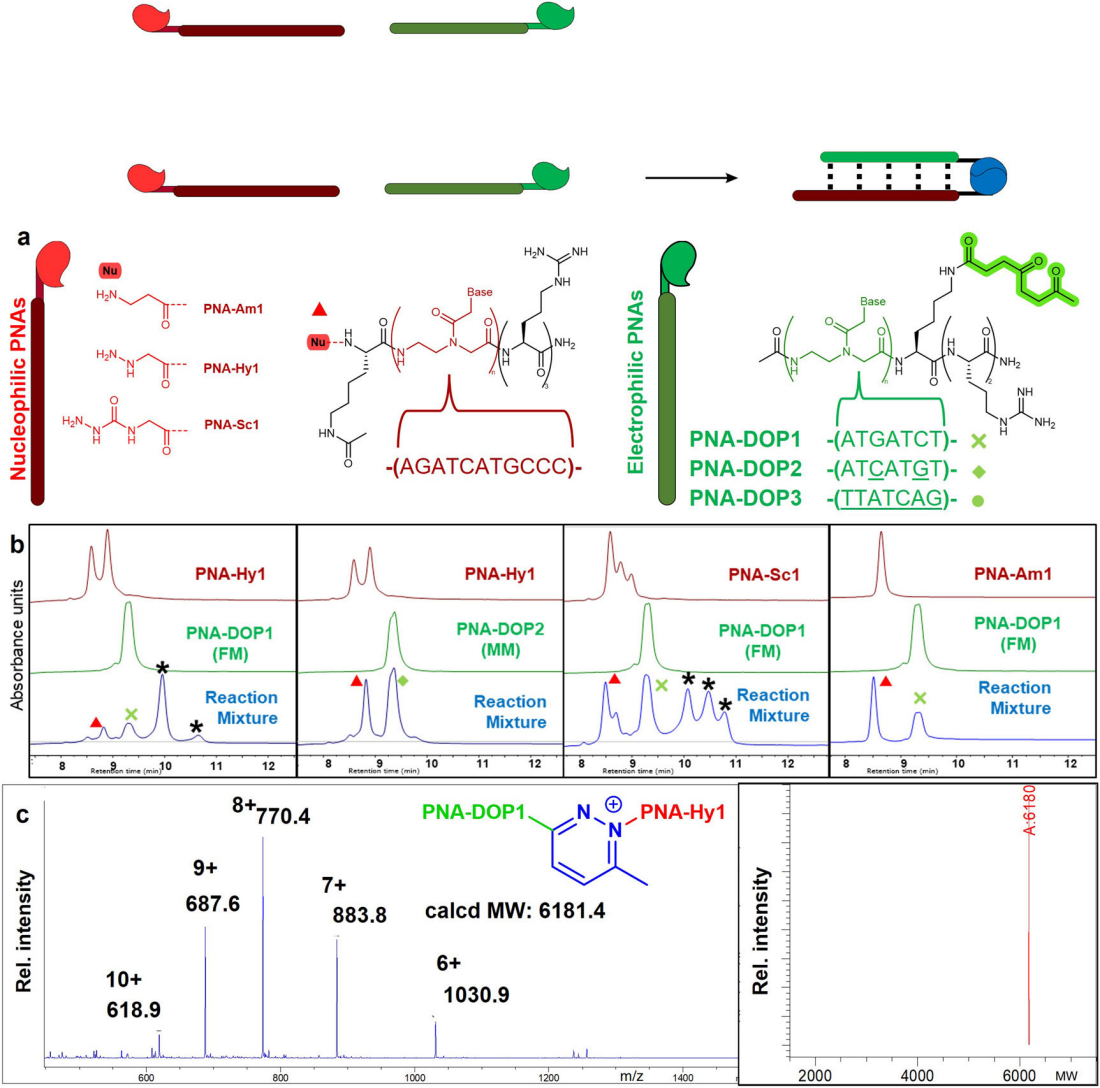
PNA-PNA ligation. **a** Structure and sequence of the PNA probes; mismatched bases are underlined. **b** HPLC-UV traces of PNA-PNA ligation experiments (red trace: nucleophilic PNA reference; green trace: DOP PNA reference; blue trace: reaction mixture after o.n. incubation). **c** ESI-MS spectrum of the ligation product peak (left) and corresponding deconvolution (right). All experiments were performed at 5 μM strand concentration in PBS buffer pH 7.4, 25 °C, overnight. * Indicates confirmed ligation product (all peaks, once isolated showed identical MW), red triangles indicate the peaks corresponding to the nucleophilic probes, green cross indicates the peak of fully matched **PNA-DOP1**, and green diamond indicates the peak of mismatched **PNA-DOP2**. The multiple peaks present in **PNA-Hy1** and **PNA-Sc1** reference chromatograms correspond to the same species in different protonation states. FM: full match, MM: mismatch.

Different 2,5-DOP-containing PNA probes were synthesized and their potential ligation behavior was tested in presence of PNA probes equipped with different nucleophiles (structure and sequence of DOP- and nucleophile-containing probes are shown in Fig. 2a). The HPLC-UV analyses of the PNA-PNA ligation experiments, performed at 5 μM probe concentration in PBS pH 7.4, are shown in Fig. 2b (also see the Supplementary Information for additional HPLC-UV and Urea-SDS-PAGE analyses, Supplementary Figs. 4–9). The formation of a ligation product was observed only when fully matched (FM) **PNA-DOP1** is employed, and highly reactive α-effect nucleophiles are installed on the target strands **PNA-Hy1** (containing a hydrazine moiety) and **PNA-Sc1** (containing a semicarbazide moiety). The formation of a covalent ligation product was further confirmed via HPLC-ESI-MS (see Fig. 2c and Supplementary Fig. 10). Importantly, the reaction proved highly selective as no ligation product was detected for mismatched (**PNA-DOP2**) or scrambled (**PNA-DOP3**) sequences, underscoring the proximity dependence of the reported reaction. For the mismatched and scrambled cases, even when performing multiple freeze-thawing cycles^32^, using longer probes or higher concentrations and long reaction times (200 μM for 5 days) no ligated products could be detected (Supplementary Figs. 11–13), further confirming the need for close positioning of the functional groups within the fully hybridized duplexes.

Interestingly, nucleophilic probes bearing alkylic hydrazides and various (unreactive) nucleophiles (e.g., a terminal lysine), did not lead to the formation of any ligation product (see Supplementary Information, Supplementary Figs. 4, 5, 8, and 14).

Finally, the ligation was evaluated in cell lysate (reducing conditions) and under more challenging oxidative conditions. In both scenarios the reaction outcome was not affected, validating the tolerance of this system to a broad range of conditions (see Supplementary Information, Supplementary Figs. 15 and 16).

### Characterization of the ligation product

It is well known that 2,5-diones can be converted to the corresponding pyrroles under Paal-Knorr conditions in presence of amines^33–39^, while 2,4-diones lead to the formation of pyrazoles when hydrazine or hydrazide nucleophiles are employed^31,40^. Nevertheless, reports describing the reaction between 2,5-diones and α-effect nucleophiles (usually performed at high temperatures and under acid catalysis) are scarce and provide poor product characterization^41–45^. For this reason, we decided to investigate the nature of the formed ligation products using representative small molecules.

**Fig. 3:**
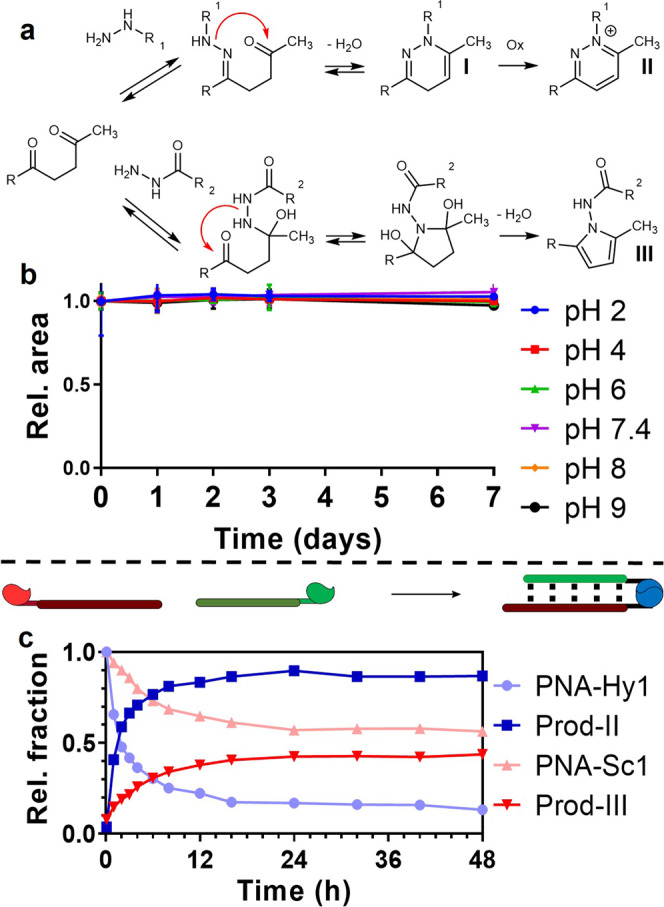
PNA-ligation product stability and kinetics of formation. **a** Proposed reaction mechanisms and product structures. **b** Evaluation of the stability of the pyridazinium moiety at 37 °C at different pH values. **c** Kinetics of formation of ligation product of type **II** (dark blue) and **III** (dark red) in a PNA:PNA geometry; light colors show starting material consumption.

Hexan-2,5-dione was reacted with benzylhydrazine using similar conditions as exploited in the previous PNA-PNA ligation experiments (i.e., neutral pH and r.t). After screening different experimental parameters, it was found that in DMF and DMSO (the only solvents that allowed to ensure high reagent concentration), product formation could only be observed (albeit in low yield) using such very high reactant concentrations (2.2 M) and long reaction times (3 days). The formed product was identified as a pyridazinium derivative of type **II** (Fig. 3a) based on ^1^H-NMR (Supplementary Fig. 17). Detailed HPLC monitoring revealed the fast formation of a species with higher polarity, which is then converted into the final pyridazinium form. We hypothesize a relatively fast formation of a dihydropyridazine derivative of type **I**, which is then slowly oxidized to the final aromatic derivative.

No product formation was observed in presence of aliphatic hydrazides, while a N-(1H-pyrrol-1-yl)amide derivative of type **III** was isolated when benzhydrazide was used (Supplementary Fig. 18). Due to the lower solubility of semicarbazides, it was not possible to isolate and confirm the structure of the reaction product with small molecules. Given the lack of nucleophilicity of the nitrogens in alpha to the carbonyl, we can surmise that the reaction with the semicarbazide follows a similar pathway as the one observed for benzhydrazide, leading to the formation of a pyrrol-like ligation product of type **III**. Putative ligation mechanisms are depicted in Fig. 3a.

The obtained ligation product of type **II** was shown to be remarkably stable when compared to classical hydrazone ligation products. Within the pH range 2–9, the pyridazinium linkage showed no significant degradation even after 7 days at 37 °C (Fig. 3b). Additionally, under neutral or acidic conditions the ligation product proved to be stable when heated at 90 °C for 24 h, with minor hydrolysis being observed under basic conditions (16% degradation at pH 9, Supplementary Fig. 19).

PNA-PNA ligation kinetics were then evaluated at low probe concentration and neutral pH, monitoring the ligation reaction at 5 μM **PNA-Hy1** or **PNA-Sc1** probe concentration in presence of a slight excess (1.1 eq.) of complementary **PNA-DOP1**. A faster conversion profile was observed for hydrazine-containing **PNA-Hy1** as compared to the semicarbazide-containing **PNA-Sc1** (Fig. 3c). Indeed, a 50% conversion of **PNA-Hy1** occurs within 2 h with a steady increase until 85% after 16 h (blue line). In contrast, only 15% conversion was observed for **PNA-Sc1** after 2 h and only about 40% conversion was reached after 24 h. Measured half-lives for hydrazine and semicarbazide conversion are 27 min and 4.5 h, respectively. For these reasons, semicarbazide-modified probes were excluded, leaving hydrazine as the only candidate for further investigations.

### DNA-templated ligation

**Fig. 4:**
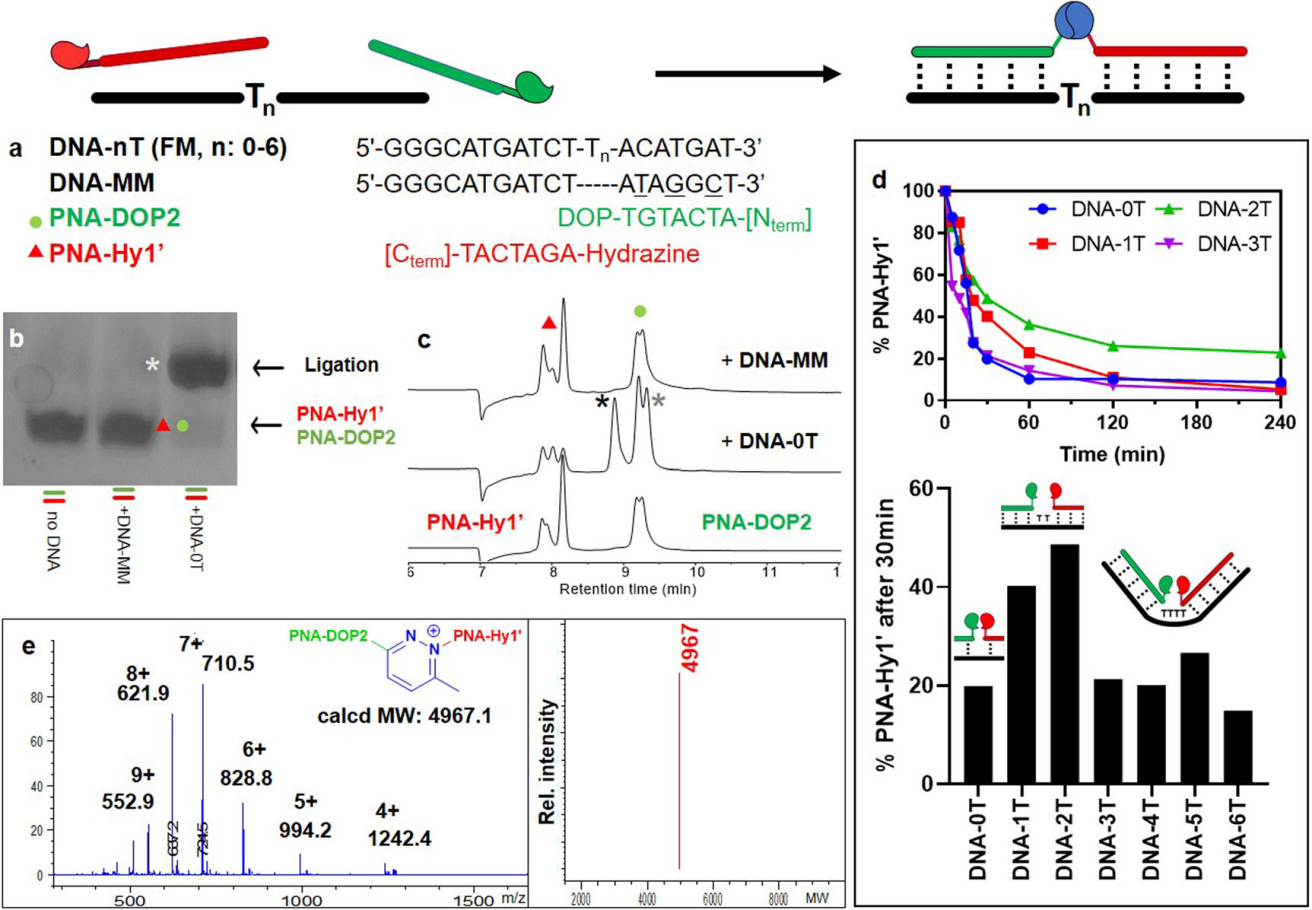
DNA-templated PNA-PNA ligation. **a** Sequences of PNA and DNA probes. Urea-SDS-PAGE (**b**) and HPLC-UV (**c**) of DNA-templated ligation experiments performed between **PNA-DOP2** and **PNA-Hy1’** (bottom: no DNA; middle: in presence of fully matching **DNA-0T**, top: in presence of mismatched **DNA-MM**). **d** Consumption profiles of **PNA-Hy1’** in presence of different DNA strands (*n* = 0–3, top) and percentage of starting material left after 30 min of reaction in presence of different DNA strands (*n* = 0–6, bottom). **e** ESI-MS spectra of the ligation product peak (left) and corresponding deconvolution (right). All experiments were conducted at 5 μM strand concentration in PBS buffer pH 7.4, 25 °C, overnight. *: confirmed ligation product (all peaks, once isolated showed identical MW, the observed different retention times were attributed to partial PNA:DNA complex melting during HPLC analysis: black star single stranded PNA, gray star PNA:DNA duplex). Double peaks present in **PNA-Hy1** and **PNA-DOP2** reference chromatograms correspond to the same species in different protonation states. FM: full match, MM: mismatch.

Next, we set out to evaluate the possibility of forming a ligation product between a DOP-probe and a shorter non-complementary hydrazine-containing PNA (7mer, **PNA-Hy1’**) using a complementary DNA templating strand as an alternative way to generate the required proximity. After overnight incubation of the two PNA probes with different DNA sequences, the formation of the desired ligation product could only be observed when a fully complementary DNA probe was employed, as confirmed by Urea-SDS-PAGE, HPLC, and ESI-MS (Fig. 4 and Supplementary Figs. 20–23).

The presence of a DNA strand to direct the two reactive units also resulted in an increased reaction speed as a consequence of a better orientation of the two reactive functionalities. On the other hand, the insertion of an oligonucleotide gap to outdistance the reactive groups, resulted in a consistently slower reaction when one or two thymines were inserted between the two guiding regions. In the **DNA-2T** case, this reduced reaction rate also reflects in a lower conversion of the starting **PNA-Hy1’** observed after 4 h of reaction. Further insertion of nucleotides (from 3 to 6 extra thymines) resulted in a kinetic profile comparable to that in the absence of a gap. This can be ascribed to the increased flexibility of a larger single-stranded gap junction that is no longer able to keep the two reactive units apart. This allows the two flanking, double-stranded PNA:DNA, regions to move toward each other, and the consequent reaction of the two functionalities (see Fig. 4d and Supplementary Fig. 24). This behavior was confirmed by the reaction half-life: 12.3 min in presence of **DNA-0T**, 16.8 min in presence of **DNA-1T**, 40.27 min in presence of **DNA-2T**, and 15.7 min in presence of **DNA-3T**.

### Templated ligation on surface: 96-well plates and glass micro-arrays

**Fig. 5:**
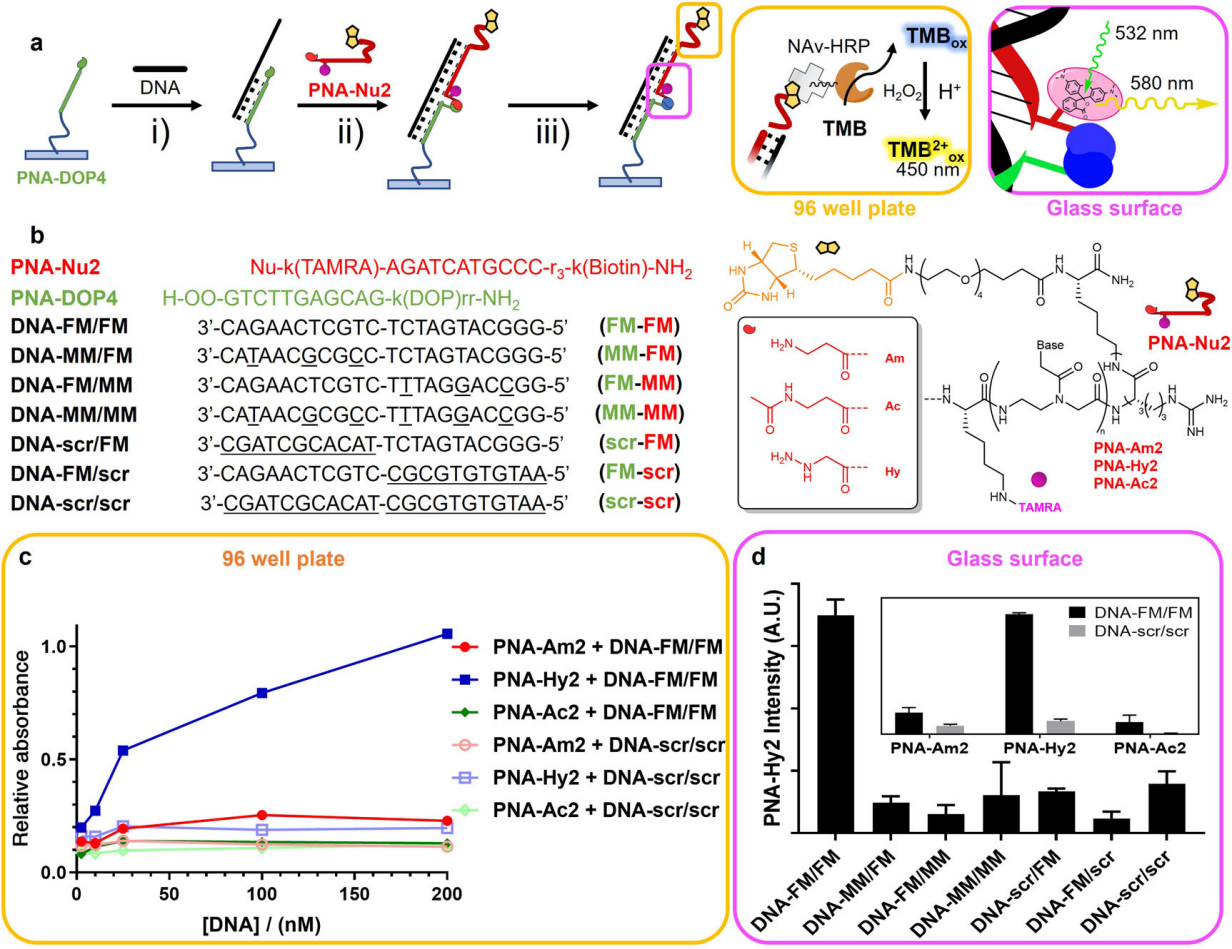
Templated ligation on surface. **a** Schematic workflow of the detection system: (i) hybridization of DNA target to surface-immobilized PNA; (ii) hybridization of the nucleophile-containing probe and formation of the PNA_2_:DNA complex; (iii) overnight incubation and formation of the PNA-PNA:DNA complex; detection of the final ligation product can be performed through incubation with NAv-HRP and evaluation of the resulting peroxidase activity (black box, for 96-well plates) or by fluorescence emission of the TAMRA reporting group (orange box, for microarray glass surfaces). **b** Structure of the nucleophilic probes and sequences used for surface experiments. **c** TMB_ox_ signal generation after overnight incubation of a 1 μM solution of **PNA-Hy2**, **PNA-Am2**, or **PNA-Ac2** in presence of different concentrations of fully matched **DNA-FM/FM** or scrambled **DNA-scr/scr** in a 96-well plate format. **d** TAMRA emission recorded after overnight incubation of 50 nM **PNA-Hy2** in presence of 50 nM of the different DNA sequences in a microarray format. The insert shows the signal generated after overnight incubation of **PNA-Hy2**, **PNA-Am2**, or **PNA-Ac2** in presence of 50 nM of fully matched **DNA-FM/FM** and scrambled **DNA-scr/scr**. O: (2-(2-aminoethoxy)ethoxy)acetyl spacer.

Given the simple mix-and-wait conditions required for the hydrazine-DOP ligation, we tested the possibility to translate this chemistry to more challenging on-surface scenarios, such as 96-well plates and microarray slides (Fig. 5a). In short, a DOP-containing PNA probe was covalently linked to the surface and exploited as capture probe for the target DNA sequence. In turn, the formed complex is able to recruit a hydrazine-containing PNA from the solution, complementary to the non-paired part of the DNA target sequence, and acts as reporter probe (containing a biotin or rhodamine tag). The recruiting of the two PNA probes to the surface, templated by the target DNA, allows for the ligation to take place. In the plastic 96-well plate format, the product formation was monitored by the recognition of the biotin-tag by a Neutravidin-Horseradish peroxidase (NAv-HRP) conjugate and measurement of the resulting peroxidase activity via 3,3′,5,5′-tetramethylbenzidine (TMB) oxidation, in an ELISA-like detection format. Alternatively, on microarray glass surfaces quantification could be directly achieved using a fluorescent tetramethylrhodamine (TAMRA)-tag. Figure 5a depicts the complete reaction and detection schemes for both scenarios. For the current scenarios, a longer DOP-containing **PNA-DOP4** (sequence included in Fig. 5b) was synthesized and linked to the surface, and the surface-ligation reaction was evaluated in presence of different DNA sequences and nucleophilic PNAs.

In the 96-well plate format, without any special optimization or harsh washing steps, sequence selectivity and reaction chemoselectivity were confirmed in the low nM range (as shown in Fig. 5c). Only very low signal intensities were obtained with scrambled DNA targets. In particular, the absence of a TMB_ox_ signal in presence of any of the non-reactive nucleophilic PNAs (**PNA-Am2** (red trace), **PNA-Ac2** (green trace)) further validates that the signal obtained with **PNA-Hy2** (blue trace) results from the formation of a covalent bond rather than a very stable PNA_2_:DNA complex. Using 200 nM of target DNA in presence of a 1 μM solution of **PNA-Hy2**, it was possible to obtain quantitative formation of the desired ligation product. Indeed, the generated TMB_ox_ signal was as intense as the one generated in the positive control well, where the biotin-containing **PNA-Am2** was directly linked to the well surface. Similar results were obtained on a microarray glass surface using 50 nM PNA and DNA probe concentration (Fig. 5d and Supplementary Fig. 25).

### Peptide-peptide ligation

**Fig. 6:**
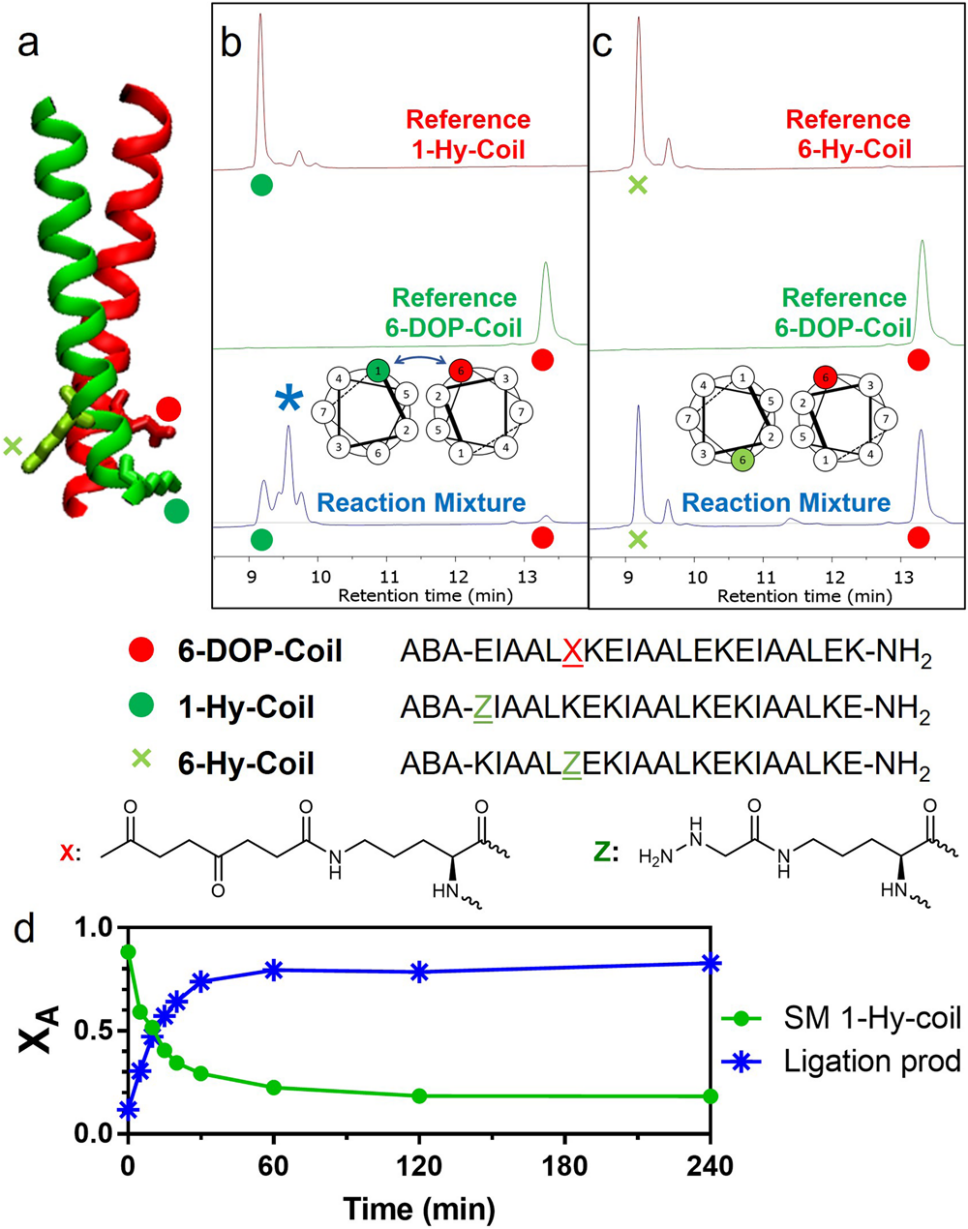
Coiled-coil ligation. **a** Schematic representation of the coiled-coil structure and disposition of modification sites based on the 4EPT crystal structure. **b** HPLC trace of the ligation experiment (blue) between **6-DOP-Coil** (red) and **1-Hy-Coil** (green dot). **c** HPLC trace of the ligation experiment (blue) between **6-DOP-Coil** (red) and **6-Hy-Coil** (green cross). **d** Reaction kinetics in the coiled-coil system. *: ligation product; ABA: 4-acetamidobenzoic acid. All experiments were conducted at 5 μM coil concentration in PBS buffer pH 7.4, 25 °C, overnight.

Finally, in order to broaden the scope of this proximity-induced ligation, we investigated the possibility to translate this approach to peptide-peptide ligation, exploiting the supramolecular recognition between α-helix coiled-coils^46–48^. As model we selected the synthetic heterodimeric system (EIAALEK)_3_/(KIAALKE)_3_ able to form a parallel coiled-coil structure, also employed for the dimerization of the Kar3Vik1 protein^49,50^. The original structure of the two peptides was modified to accommodate the two required functional groups. In particular, to minimize the perturbation induced by the sequence modification, the DOP moiety was appended on an ornithine side chain replacing the glutamic acid at position 6 of the first heptad of the E-rich coil (**6-DOP-Coil**, see Fig. 6). A hydrazine-modified ornithine was exploited for the replacement of either the lysine at position 1 or 6 of the first heptad of the K-rich coil strand (**1-Hy-Coil** and **6-Hy-Coil**, respectively). As shown in Fig. 6, upon overnight incubation of the two coils at 5 μM concentration, selective formation of the desired ligation product of **6-DOP-Coil** occurs in presence of **1-Hy-Coil**, where the required hydrazine function is correctly oriented on the same side of the coiled-coil structure.

In this architecture the reaction proved to have a similar kinetic profile as for the DNA-templated case, with a half-life of 11.9 min, resulting in 70% **1-Hy-Coil** conversion in under 30 min and reaching a plateau at 80% probe conversion after 2 h (Fig. 6d). Results were further validated on the hydrazine-containing arginine (RIAALRE)_3_, analog of the K-rich coil, were all lysines were replaced by arginines and in presence of a short hydrazine-containing random peptide (see the Supplementary Information, Supplementary Figs. 24 and 27). All ligation products were confirmed by HPLC-MS (see the SI, Supplementary Figs. 28 and 29).

### Conclusions

We here reported on a proximity-induced ligation methodology between biomolecules functionalized with two small, very stable and bio-orthogonal, functionalities: a hydrazine and a γ-diketone moiety, respectively. The main advantages of this ligation approach reside in the catalyst-free reaction conditions leading to the formation of a covalent link without any external trigger and resulting in a ligation product that was proven to be very stable under a broad range of conditions. The reaction was shown to proceed under physiologically relevant conditions (low probe concentration, neutral pH, cell lysate) and proven to be selective even at higher probe concentration, extending its possible application to a broader range of conditions. In addition, the chemical and enzymatic stability of the required functionalities and the tolerance of other functional groups allows their introduction in a broad variety of functional molecules, without the need for special care to preserve the reactivity of the system, a problem that commonly occurs in other approaches that rely on trigger-free systems (cfr. oxidation or hydrolysis of sensitive reactive functionalities). This reflects positively on the shelf-life of the current probes, which can be stored for prolonged times without affecting their reactivity. Furthermore, the possibility to readily include the electrophilic system through a small 2,5-dialkylfuran, and the possibility to directly obtain a “ligation-ready” probe during the final step of deprotection/cleavage from the solid support, renders this ligation particularly suited for peptides and peptide-like probes.

The examples shown in this work demonstrate the potential of this reaction, which tolerates different architectures in solution and the possibility to extend it to surface applications, including the realization of self-addressable systems. Given the simplicity, robustness, and complete bio-orthogonality of the functional groups involved, this hydrazine-DOP ligation can foster the development of stable, self-assembled supramolecular architectures. This will enable the possibility to re-establish a central role of chemical engineering in the realization of bottom-up approaches that nowadays are only accessible through cumbersome biological manipulations, such as in DNA- and protein-origami technologies^51,52^. In particular, functionalities that can lead to selective proximity-induced ligation will allow breaking down these big macromolecules to smaller and chemically accessible building blocks that can be connected on-demand while avoiding enzyme-based ligations.

## Methods

### PNA-PNA ligation

In a typical experiment, 100 µL of buffered solutions (PBS pH 7.4) containing probes at 5 µM concentration (from a 100 μM stock solution), were prepared in a 0.5 mL Eppendorf and allowed to react overnight at 25 °C. The solutions were collected in the morning and analyzed via HPLC-UV, HPLC-MS, and USDS-PAGE.

### DNA templated PNA-PNA ligation

In a typical experiment, 100 µL of buffered solutions (PBS pH 7.4) containing all probes at 5 µM concentration (from a 100 μM stock solution), were prepared in a 0.5 mL Eppendorf. The complex between DNA and the nucleophilic PNA was allowed to equilibrate for 5 min before the final addition of DOP-PNA probe, and the mixtures were allowed to react overnight at 25 °C. The solutions were collected in the morning and analyzed via HPLC-UV, HPLC-MS, and USDS-PAGE.

### 96-well plate functionalization

In a typical experiment, 100 µL of buffered solutions (PBS pH 7.4) containing all probes at 5 µM concentration (from a 100 μM stock solution), were prepared in a 0.5 mL Eppendorf. The complex between DNA and the nucleophilic PNA was allowed to equilibrate for 5 min before the final addition of DOP-PNA probe, and the mixtures were allowed to react overnight at 25 °C. The solutions were collected in the morning and analyzed via HPLC-UV, HPLC-MS, and USDS-PAGE.

### Surface templated ligation in 96-well plate

Oligonucleotides and PNA solutions were freshly prepared in PBS pH 7.4 supplemented with 0.001% SDS (PBS-S) from a 10 μM stock solutions in mQ. Surfaces were pre-wetted for 30 min with a 0.001% SDS solution. Then, 50 μL of oligonucleotide and 50 μL of 1 μM PNA solutions were allowed to react overnight at 40 °C. Wells were washed with a mQ/MeCN 1:1 + 0.1% TFA solution (4 × 5 min, 45 °C) before the quantification of the attached biotin, using 100 μL of 20 ng/mL Pierce High Sensitivity NeutrAvidin-HRP-conjugate (Thermo Scientific) and 1-step Ultra TMB-ELISA (Thermo Scientific) as reagent solution. Final readout of the oxidized TMB was performed by monitoring the absorption at 450 nm after quenching the reaction with 2 M H_2_SO_4_.

### Microarray slide functionalization

NHS-active ester XL-CX slides (Xantec, Germany) were used as solid support. Functionalization was performed spotting 0.3 μL of a 1 μM PNA solution in 100 mM carbonate buffer pH 9.0 containing 30% glycol and 0.0001% SDS. Functionalization was allowed overnight in a humid chamber (75% relative humidity) and remaining active sites were quenched for 4 h using a 6% ethanolamine solution in 100 mM carbonate buffer pH 9.0. Finally, surfaces were washed with a 0.01% SDS solution (10 min, twice), 0.001% SDS solution (10 min, twice), and mQ. Slides were dried with a stream of clean air and stored over CaCl2. All steps were performed away from direct light.

### Surface template ligation on microarray slides

Oligonucleotide and PNA solutions were freshly prepared in PBS-S from a 10 μM stock solution in mQ. Surfaces were pre-wetted for 30 min with a 0.001% SDS solution and then dried with a stream of clean air, before the application of the desired mask (in a typical experiment a 16 well mask is employed). 50 μL of oligonucleotide and 50 μL of 100 nM PNA solutions were added and allowed to react overnight at 40 °C. Slides were washed in PBS pH 7.4, supplemented with 0.05% TWEEN-20 (2×10 min, 50 °C) and mQ (1 min, r.t.). Slides were then dried with a stream of clean air before image acquisition. All steps were performed away from direct light.

### Proximity induced peptide ligation

In a typical experiment, 100 µL of buffered solution (PBS pH 7.4) containing probes at 5 µM concentration (from a 100 μM stock solution), were prepared in a 0.5 mL Eppendorf and allowed to react overnight at 25 °C. The solutions were collected in the morning and analyzed via HPLC-UV, HPLC-MS.

## Supplementary information

Supplementary Materials

## Data Availability

All data generated or analyzed during this study are included in this published article (and its Supplementary Information file.

## References

[1] Zhang Y, Park KY, Suazo KF, Distefano MD (2018). Recent progress in enzymatic protein labelling techniques and their applications. Chem. Soc. Rev..

[2] Liu G, Hu J, Liu S (2018). Emerging applications of fluorogenic and non-fluorogenic bifunctional linkers. Chem. A Eur. J..

[3] Sletten EM, Bertozzi CR (2009). Bioorthogonal chemistry: fishing for selectivity in a sea of functionality. Angew. Chem. Int. Ed..

[4] Pavagada S (2019). Oligonucleotide-templated lateral flow assays for amplification-free sensing of circulating microRNAs. Chem. Commun..

[5] Domaille DW, Cha JN (2014). Aniline-terminated DNA catalyzes rapid DNA-hydrazone formation at physiological pH. Chem. Commun..

[6] Velema WA, Kool ET (2017). Fluorogenic templated reaction cascades for RNA detection. J. Am. Chem. Soc..

[7] Patzke V, McCaskill JS, Von Kiedrowski G (2014). DNA with 3′-5′-disulfide links - Rapid chemical ligation through isosteric replacement. Angew. Chem. Int. Ed..

[8] Sayers J, Payne RJ, Winssinger N (2018). Peptide nucleic acid-templated selenocystine–selenoester ligation enables rapid miRNA detection. Chem. Sci..

[9] Reinhardt U (2014). Peptide-templated acyl transfer: a chemical method for the labeling of membrane proteins on live cells. Angew. Chem. Int. Ed..

[10] Brauckhoff N, Hahne G, Yeh JTH, Grossmann TN (2014). Protein-templated peptide ligation. Angew. Chem. Int. Ed..

[11] Kern A, Seitz O (2015). Template-directed ligation on repetitive DNA sequences: a chemical method to probe the length of Huntington DNA. Chem. Sci..

[12] Fan W (2018). Click chemical ligation-initiated on-bead DNA polymerization for the sensitive flow cytometric detection of 3′-terminal 2′-O-methylated plant microRNA. Anal. Chem..

[13] Shelbourne M, Chen X, Brown T, El-Sagheer AH (2011). Fast copper-free click DNA ligation by the ring-strain promoted alkyne-azide cycloaddition reaction. Chem. Commun..

[14] Werther P, Möhler JS, Wombacher R (2017). A bifunctional fluorogenic rhodamine probe for proximity-induced bioorthogonal chemistry. Chem. A Eur. J..

[15] Šečkute J, Yang J, Devaraj NK (2013). Rapid oligonucleotide-templated fluorogenic tetrazine ligations. Nucleic Acids Res..

[16] El-Sagheer AH, Cheong VV, Brown T (2011). Rapid chemical ligation of oligonucleotides by the Diels-Alder reaction. Org. Biomol. Chem..

[17] Metcalf GADD (2016). Amplification-free detection of circulating microRNA biomarkers from body fluids based on fluorogenic oligonucleotide-templated reaction between engineered peptide nucleic acid probes: application to prostate cancer diagnosis. Anal. Chem..

[18] Yang B (2018). Proximity-enhanced SuFEx chemical cross-linker for specific and multitargeting cross-linking mass spectrometry. Proc. Natl Acad. Sci. USA.

[19] Anzola M, Winssinger N (2019). Turn On of a Ruthenium(II) Photocatalyst by DNA‐Templated Ligation. Chem. A Eur. J..

[20] Manicardi A, Cadoni E, Madder A (2020). Visible-light triggered templated ligation on surface using furan-modified PNAs. Chem. Sci..

[21] Mendez-Gonzalez D (2018). Photochemical ligation to ultrasensitive DNA detection with upconverting nanoparticles. Anal. Chem..

[22] De Fazio AF (2019). Light-induced reversible DNA ligation of gold nanoparticle superlattices. ACS Nano.

[23] Harimech PK, Gerrard SR, El-Sagheer AH, Brown T, Kanaras AG (2015). Reversible ligation of programmed DNA-gold nanoparticle assemblies. J. Am. Chem. Soc..

[24] Kölmel DK, Kool ET (2017). Oximes and hydrazones in bioconjugation: mechanism and catalysis. Chem. Rev..

[25] Agarwal P, Van Der Weijden J, Sletten EM, Rabuka D, Bertozzi CR (2013). A Pictet-Spengler ligation for protein chemical modification. Proc. Natl Acad. Sci. USA.

[26] Agarwal P (2013). Hydrazino-pictet-spengler ligation as a biocompatible method for the generation of stable protein conjugates. Bioconjug. Chem..

[27] Cambray S, Gao J (2018). Versatile bioconjugation chemistries of ortho-boronyl aryl ketones and aldehydes. Acc. Chem. Res..

[28] Mailig M, Liu F (2020). Pyrazolone ligation-mediated versatile sequential bioconjugations. Chem. Sci..

[29] Mailig M, Hymel D, Liu F (2020). Further exploration of hydrazine-mediated bioconjugation chemistries. Org. Lett..

[30] Antonatou E, Verleysen Y, Madder A (2017). Singlet oxygen-mediated one-pot chemoselective peptide-peptide ligation. Org. Biomol. Chem..

[31] Wu WN (2018). A highly sensitive and selective off–on fluorescent chemosensor for hydrazine based on coumarin β-diketone. Spectrochim. Acta Part A Mol. Biomol. Spectrosc..

[32] Agten SM, Suylen DPL, Hackeng TM (2016). Oxime catalysis by freezing. Bioconjug. Chem..

[33] Paal C (1885). Synthese von thiophen- und pyrrolderivaten. Ber. der Dtsch. Chem. Ges..

[34] Knorr L (1884). Synthese von pyrrolderivaten. Ber. der Dtsch. Chem. Ges..

[35] Truong Nguyen H, Nguyen Chau D-K, Tran PH (2017). A green and efficient method for the synthesis of pyrroles using a deep eutectic solvent ([CholineCl][ZnCl_2_]_3_) under solvent-free sonication. N. J. Chem..

[36] Minetto G, Raveglia LF, Sega A, Taddei M (2005). Microwave-assisted Paal-Knorr reaction—three-step regiocontrolled synthesis of polysubstituted furans, pyrroles and thiophenes. Eur. J. Org. Chem..

[37] Bharadwaj AR, Scheidt KA (2004). Catalytic multicomponent synthesis of highly substituted pyrroles utilizing a one-pot Sila-Stetter/Paal−Knorr strategy. Org. Lett..

[38] Nguyen HT, Thuy Nguyen LH, Le Hoang Doan T, Tran PH (2019). A mild and efficient method for the synthesis of pyrroles using MIL-53(Al) as a catalyst under solvent-free sonication. RSC Adv..

[39] Dasari R, La Clair JJ, Kornienko A (2017). Irreversible protein labeling by Paal-Knorr conjugation. ChemBioChem.

[40] Flood DT (2018). Leveraging the Knorr Pyrazole synthesis for the facile generation of thioester surrogates for use in native chemical ligation. Angew. Chem. Int. Ed..

[41] Overberger CG, Byrd NR, Mesrobian RB (1956). Azo compounds. investigation of the structure of the products from the reaction of acetonylacetone and hydrazine. J. Am. Chem. Soc..

[42] Allah HMF, Soliman R (1987). Cyclization of diketones to pyridazine and furan derivatives. J. Heterocycl. Chem..

[43] Lie Ken Jie MSF, Kalluri P (1997). Synthesis of pyridazine fatty ester derivatives in water: a sonochemical approach. J. Chem. Soc. Perkin Trans. 1.

[44] Touil S, Zantour H (1998). Action des hydrazines Sur Les γ,ß′-dicarbonylphosphonates et phosphineoxides: synthese de 4-phosphopyridazines et pyridazin-3-ones. Phosphorus Sulfur. Silicon Relat. Elem..

[45] Al-Hossainy AF (2016). Synthesis, spectral, thermal, optical dispersion and dielectric properties of nanocrystalline dimer complex (PEPyr-diCd) thin films as novel organic semiconductor. Bull. Mater. Sci..

[46] Litowski JR, Hodges RS (2002). Designing heterodimeric two-stranded α-helical coiled-coils. Effects of hydrophobicity and α-helical propensity on protein folding, stability, and specificity. J. Biol. Chem..

[47] Rink WM, Thomas F (2019). De novo designed α-helical coiled-coil peptides as scaffolds for chemical reactions. Chem. A Eur. J..

[48] Gavins GC (2021). Live cell PNA labelling enables erasable fluorescence imaging of membrane proteins. Nat. Chem..

[49] Lindhout DA, Litowski JR, Mercier P, Hodges RS, Sykes BD (2004). NMR solution structure of a highly stable de novo heterodimeric coiled-coil. Biopolymers.

[50] Rank KC (2012). Kar3Vik1, a member of the Kinesin-14 superfamily, shows a novel kinesin microtubule binding pattern. J. Cell Biol..

[51] Seeman Nadrian C. & Sleiman Hanadi F. *DNA nanotechnology*. *Nature Reviews Materials* **3** (2018).

[52] Ljubetič A (2017). Design of coiled-coil protein-origami cages that self-assemble in vitro and in vivo. Nat. Biotechnol..

