# Supplementary material for: Hydrolysis of 5-methylfuran-2-yl to 2,5-dioxopentanyl allows for stable bio-orthogonal proximity-induced ligation
Source: Commun Chem. 2021 Oct 22;4:146. doi: 10.1038/s42004-021-00584-1 (PMC9814669; doi:10.1038/s42004-021-00584-1)
Supplement: Supplementary file 1 — Supplementary Materials [file 42004_2021_584_MOESM1_ESM.pdf]

Hydrolysis of 5-methylfuran-2-yl to 2,5-dioxopentanyl allows for stable biorthogonal proximity-induced ligation

Alex Manicardi*, Enrico Cadoni, and Annemieke Madder*

Supporting Information

Table of contents

1. General information 2
  1.1 PNA synthesis 3
  1.2 Peptide synthesis 3
2. PNA:PNA ligation 6
  2.1 Sequence and nucleophile selectivity of 2.5-DOP ligation 6
  2.2 2,5-DOP ligation at higher concentration 12
  2.3 2,5-DOP ligation in presence of a lysine terminated PNA 15
  2.4 Effect of oxidizing conditions on the ligation reaction 16
  2.5 2,5-DOP ligation in cell lysate 16
3. Structural investigation on small molecules 18
4. Stability evaluation of the pyridazinium ligation product 20
5. DNA templated PNA-PNA ligation 21
6. Surface ligation 23
  6.1 96-well plate functionalization 23
  6.2 Surface template ligation in 96-well plate 23
  6.3 Microarray slide functionalization 23
  6.4 Surface template ligation on microarray slides 23
7. Proximity induced peptide ligation 25
8. Supplementary References 27
9. HPLC-MS chromatograms of pure PNAs and peptides 28

1. General information

All reagents were purchased from Sigma-Aldrich, Fluka, Merck, TCI Europe, fluorochem and used without further purification. Dry DMF was stored over 4 Å molecular sieves. DNA and RNA sequences were purchased from IDT (Leuven, Belgium).

NMR spectra were recorded on a Brucker Avance 300 or 400. δ values are expressed in ppm relatively either to $CDCl_3$ (7.29 ppm for proton and 76.9 ppm for carbon) or DMSO-d^6 (2.50 ppm for proton and 39.5 ppm for carbon). The following abbreviations are used to explain the multiplicities: s=singlet, d=doublet, t=triplet, q=quartet, m=multiplet, and br=broad.

HPLC-MS data were collected on an Agilent 1100 Series instrument equipped with a Phenomenex Kinetex C18 100 Å column (150 x 4.6 mm, 5 µm at 35 °C) connected to an ESMSD type VL mass detector (quadrupole ion trap mass spectrometer) with a flow rate of 1.5 ml/min was used with the following solvent systems: (A): 0.1% HCOOH in H_2O and (B) MeCN. Gradient: 100% A for 2 min, then a gradient from 0 to 100% B over 6 min was used, followed by 2 min of flushing with 100% B (HPLC1) or 100% A for 0.5 min, a gradient from 0 to 10% B over 0.1 min and then from 10 % to 30 % B over 7.7 minutes was used, followed by 2 min of flushing with 100% B (HPLC2).

HPLC-UV data were collected on an Agilent 1100 Series instrument equipped with a Waters XTERRA RP18 5µm column (250 x 2.1 mm at 40°C or 50°C) connected to a DAD using a flow rate of 0.35 ml/min with the following solvent systems: (A): 0.1% TFA in H_2O and (B) 0.1% TFA in MeCN. Gradient: 100% A for 1 min, then a gradient from 0 to 10% B in 1 min, then to 30% B in 10 min, and finally to 100% B in 1 min, followed by 3.5 min of flushing with 100% B (HPLC3); 100% A for 1 min, then a gradient from 0 to 30% B in 1 min, then to 60% B in 10 min, and finally to 100% B in 1 min, followed by 3.5 min of flushing with 100% B (HPLC4). PNA oligomers were purified using a Phenomenex Luna C18(2) (5 µm, 100 Å, 250x4.6 mm) (HPLC5, 100% A for 5 min, then a gradient from 0 to 30% B over 18 min at a flow rate of 4.0 ml/min). Peptide oligomers were purified using a Phenomenex Luna C18(2) (5 µm, 250 Å, 250x21.2 mm) on an Agilent 218 solvent delivery system (HPLC6, 100% A for 2 min, then a gradient to 100% in 30 min at a flow rate of 17.5 ml/min).

UV-VIS spectra were recorded using a Trinean DropSense96 UV/VIS droplet reader.

Thermal denaturation experiments were recorded on a Varian Cary 300 Bio instrument equipped with a six-cell thermostatted cell holder.

USDS-PAGE analysis were performed using 15% polyacrylamide gels (5% crosslink, 19:1 acrylamide/bisacrylamide) prepared in Tris-Acetate buffer (50mM Tris-Acetate, pH 7.6) containing 7 M urea and 0.1% SDS. The temperature of the gel was stabilized with a Julabo F12 at 25°C. The power supply used for gel electrophoresis was a consort EV202 and a voltage of 200 V for 0.75 mm thickness or 100 V for 1.0 mm thickness was used to run the gels (15 minutes pre-run). 2 µL of sample solution were mixed with 3 µL formamide and 5 µL loading buffer (100 mM Tris-Acetate pH 7.6, 7 M urea, 20 % formamide, 2 % SDS) from this mixture 5 or 8 µL were loaded on the gel. Gels were stained with Pierce Silver Stain (Thermo Fisher Scientific) and pictures were scanned with an HP Photosmart B110.

Surface ligation experiments were performed using Stuart SSM1 mini orbital shaker for uniform shaking of the wells. The temperature control was ensured by a homemade thermostat incubation chamber.

Microarray slides were scanned with an Agilent G2565CA.

Synthesis of **PNA-Am1**, **PNA-Am2**, **PNA-Ac1**, **PNA-Ac2**, **PNA-Hy1**, **PNA-Hy2**, **PNA-Sc1**, and **PNA-Hd1** was previously described.[1]

1.1 PNA synthesis

The synthesis of the PNA probes was performed under standard manual Fmoc-based solid-phase synthesis conditions using HBTU/DIPEA as coupling mixture, using commercially available Fmoc-PNA-OH monomers (Biosearch Technologies, Scotland). Rinkamide-ChemMatrix resin was first loaded with Fmoc-$Arg_{(Pbf)}$-OH, Fmoc-$Lys_{(Mtt)}$-OH or Fmoc-$Lys_{(Dde)}$-OH as first monomer (0.2 mmol/g). Modification of lysine side chains were performed after Dde (for a 5 µmol scale: shake vigorously the resin for 1h in a solution containing 250 mg hydroxylamine hydrochloride and 184 mg imidazole in 1.2 mL NMP/DMF 5:1)[2] or Mtt (0.5% BtOH·H_2O in HFIP/DCM 1:1, Mtt deprotection can be visually followed by adding 1 drop of TFA to the deprotection solution)[3] removal for 3-(5-methyfuran-2-yl-)propionate and TAMRA coupling, respectively. All coupling steps were performed using HBTU/DIPEA as activating. Cleavage was performed using a TFA/m-cresol (9:1) cleavage cocktail. After RP-HPLC purification (HPLC5), the purity and identity of the PNAs were evaluated by LC-MS (HPLC1).

1.2 Peptide synthesis

The synthesis of the peptide probes was performed under standard automatic Fmoc-based solid-phase synthesis conditions on a Syro automatic peptide synthesizer, using HBTU/DIPEA as coupling mixture and Fmoc-$Orn_{(Mtt)}$-OH as well as standard Fmoc-protected amino acids. Rink amide-AM Champion resin was directly loaded in the synthesis reactor (0.69 mmol/g). Modification of the ornithine side chain was performed after Mtt removal (0.5% BtOH·H_2O in HFIP/DCM 1:1, Mtt deprotection can be visually followed by adding 1 drop of TFA to the deprotection solution)[3] for 3-(5-methyfuran-2-yl-)propionic acid or tri-Boc-hydrazinoacetic acid coupling. All coupling steps were performed using HBTU/DIPEA as activating mixture. Cleavage was performed using a TFA/m-cresol (9:1) cleavage cocktail. After RP-HPLC purification (HPLC6), the purity and identity of the peptides were evaluated by LC-MS (HPLC1). Probe yield was calculated by dissolving the pure compounds in water and calculating the probe concentration by measuring the absorbance at 260nm (unless mentioned otherwise) using Lambert-Beer's law.

Table S1: PNA sequences employed in this study. Capital letters indicate PNA monomers, small letters indicate *L*-amino acids, modifications on the lysine or ornithine side chains are inserted inside brackets. DOP: 4,7-dioxooctanoyl; O: 2-(2-aminoethoxy)ethoxyacetyl (AEEA spacer); ABA: 4-Acetamidobenzoyl; TAMRA: 5-carboxytetramethylrhodamine;

PNA	Sequence	MW
PNA-DOP1	Ac-ATGATCT-k(DOP)rr-NH_2	2545.6
PNA-DOP2	Ac-ATCATGT-k(DOP)rr-NH_2	2545.6
PNA-DOP3	Ac-TTATCAG-k(DOP)rr-NH_2	2545.6
PNA-DOP4	H-OO-GTCTTGAGCAG-k(DOP)rr-NH_2	3919.0
PNA-DOP5	H-OO-GGGCATGATCT-k(DOP)rr-NH_2	3919.0
PNA-Am1	H-β-Ala-k(Ac)-AGATCATGCCC-rrr-NH_2	3672.8
PNA-Ac1	Ac-β-Ala-k(Ac)-AGATCATGCCC-r3-NH_2	3714.8
PNA-Hy1	H_2N-g-k(Ac)-AGATCATGCCC-rrr-NH_2	3673.8
PNA-Hy1'	H_2N-g-AGATCAT-rrr-NH_2	2458.5
PNA-Hd1	H_2N-NHCO(CH_2)$_2$CO-k(Ac)-AGATCATGCCC-rrr-NH_2	3715.9
PNA-Sc1	H_2N-NHCONH-CH2CO-k(Ac)-AGATCATGCCC-rrr-NH_2	3716.8
PNA-Am2	Biot-PEG_4-k(H-β-Ala-k(TAMRA)-AGATCATGCCC-rrr-->)-NH_2	4644.9
PNA-Hy2	Biot-PEG_4-k(H_2N-g-k(TAMRA)-AGATCATGCCC-rrr-->)-NH_2	4687.0
PNA-Ac2	Biot-PEG_4-k(Ac- β-Ala-k(TAMRA)-AGATCATGCCC-rrr-->)-NH_2	4645.9
PNA-K1	H-k-AGATCATGCCC-r_3-NH_2	3559.6
6-DOP-coil	ABA-eiaal-Orn(DOP)-keiaalekeiaalek-NH_2	2582.98
1-Hy-coil	ABA-Orn(H_2N-g-)-iaalkekiaalkekiaalke-NH_2	2498.02
6-Hy-coil	ABA-kiaal-Orn(H_2N-g-)-ekiaalkekiaalke-NH_2	2498.02
1-Hy-coil(R)	ABA-Orn(H_2N-g-)-iaalreriaalreriaalre-NH_2	2638.12
6-Hy-coil(R)	ABA-riaal-Orn(H_2N-g-)-eriaalreriaalre-NH_2	2638.12
Hy-random	H-Orn(H_2N-g-)-fgydaky-NH_2	1048.16

Table S2: DNA sequences employed in this study.

**DNA**	**Sequence**
DNA-1T	5'- GGGCATGATCT-ACATGAT -3'
DNA-2T	5'- GGGCATGATCT-TT- ACATGAT -3'
DNA-3T	5'- GGGCATGATCT-TTT- ACATGAT -3'
DNA-4T	5'- GGGCATGATCT-TTTT- ACATGAT -3'

DNA-5T	5'- GGGCATGATCT-TTTTT- ACATGAT -3'
DNA-6T	5'- GGGCATGATCT-TTTTTT- ACATGAT -3'
DNA-MM	5'- GGGCATGATCT-ATAAGCT -3'
DNA-S1	5'- GGGCATGATCT-CTGATAA -3'
DNA-S2	5'- GGGCATGATCT-CCGACAA -3'
DNA-S3	5'- GGGCATGATCT-CAGGTTA -3'
DNA-S4	5'- GGGCATGATCT-ATATAAT -3'
DNA-FF	5'-GGGCATGATCT-CTGCTCAAGAC-3'
DNA-MF	5'-GGGCATGATCT-CCGCGTAATAC-3'
DNA-FM	5'-GGCCAGGATTT-CTGCTCAAGAC-3'
DNA-MM	5'-GGCCAGGATTT-CCGCGTAATAC-3'
DNA-SF	5'-GGGCATGATCT-TACACGCTAGC-3'
DNA-FS	5'-AATGTGTGCGC-CTGCTCAAGAC-3'
DNA-SS	5'-AATGTGTGCGC-TACACGCTAGC-3'

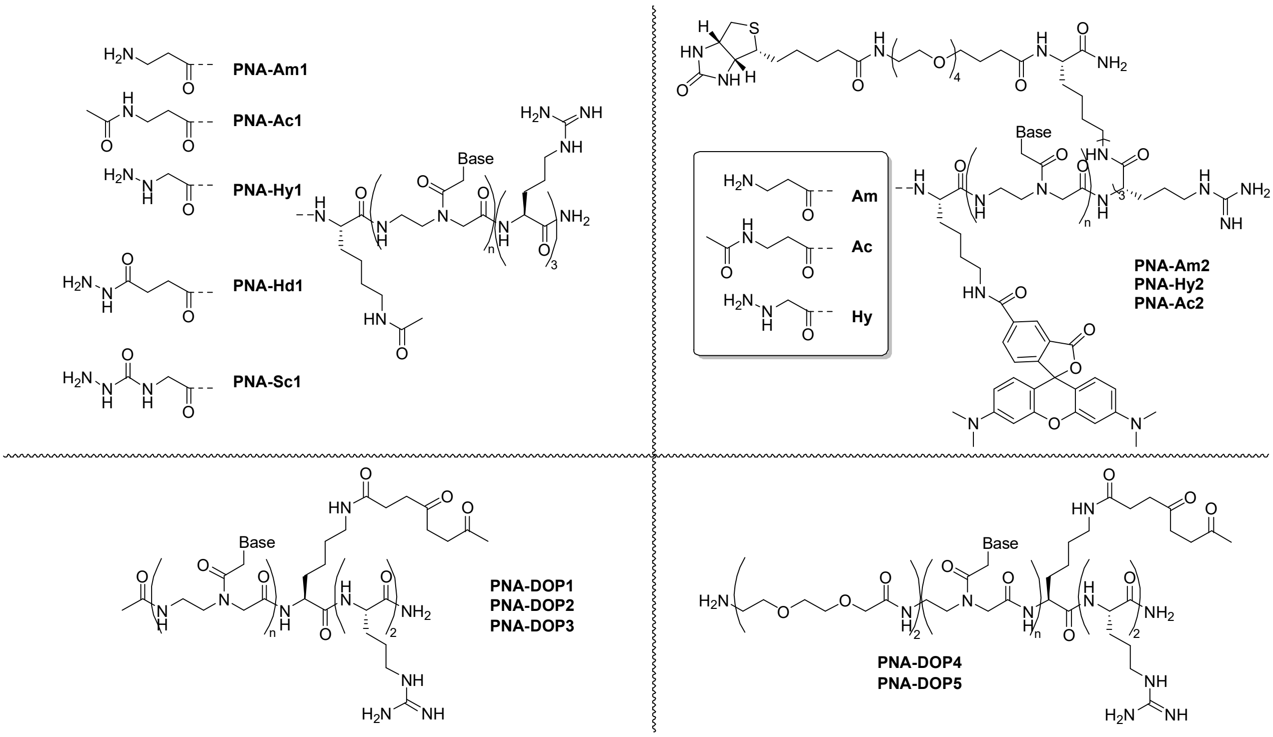

Figure S1: structures of the different PNA probes used in this study.

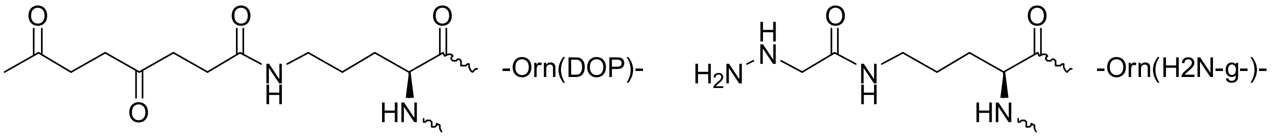

Figure S2: structures of modified amino acids employed in peptide synthesis.

PNA-Hy1': 9.3%; t_r: 2.83 min (HPLC1); ε = 76600 $M^{-1}cm^{-1}$; ESI-MS: *m/z* calcd 2458.5 [M]: 1229.7 $[M+2H]^{2+}$, 814.5 $[M+3H]^{3+}$, 615.5 $[M+4H]^{4+}$, 492.6 $[M+5H]^{5+}$, 410.7 $[M+6H]^{6+}$, 352.2 $[M+7H]^{7+}$; **PNA-DOP1:** 8.5%; t_r: 3.08 min (HPLC1); ε = 71500 $M^{-1}cm^{-1}$; ESI-MS: *m/z* calcd 2545.6 [M]: 1273.3 $[M+2H]^{2+}$, 849.2 $[M+3H]^{3+}$, 637.2 $[M+4H]^{4+}$, 510.0 $[M+5H]^{5+}$; **PNA-DOP2:** 10.0%; t_r: 3.13 min (HPLC1); ε = 71500 $M^{-1}cm^{-1}$; ESI-MS: *m/z* calcd 2545.6 [M]: 1273.3 $[M+2H]^{2+}$, 849.2 $[M+3H]^{3+}$, 637.2 $[M+4H]^{4+}$, 510.0 $[M+5H]^{5+}$, 422.2 $[M+6H]^{6+}$; **PNA-DOP3:** 8.6%; t_r: 3.13 min (HPLC1); ε = 71500 $M^{-1}cm^{-1}$; ESI-MS: *m/z* calcd 2545.6 [M]: 1273.3 $[M+2H]^{2+}$, 849.2 $[M+3H]^{3+}$, 637.2 $[M+4H]^{4+}$, 510.0 $[M+5H]^{5+}$; **PNA-DOP4:** 7.1%; t_r: 3.09 min (HPLC1); ε = 113200 $M^{-1}cm$-1; ESI-MS**:** *m/z* calcd 3918.9 [M]: 1306.9 $[M+3H]^{3+}$, 980.5 $[M+4H]^{4+}$, 784.7 $[M+5H]^{5+}$, 654.0 $[M+6H]^{6+}$, 560.8 $[M+7H]^{7+}$, 490.8 $[M+8H]^{8+}$; **PNA-DOP5:** 9.1%; t_r: 3.10 min (HPLC1); ε = 113200 $M^{-1}cm$-1; ESI-MS**:** *m/z* calcd 3918.9 [M]: 1306.9 $[M+3H]^{3+}$, 980.5 $[M+4H]^{4+}$, 784.7 $[M+5H]^{5+}$, 654.0 $[M+6H]^{6+}$, 560.7 $[M+7H]^{7+}$, 490.8 $[M+8H]^{8+}$; **PNA-K1:** 10.2%; t_r: 2.82 min (HPLC1); ε = 108100 $M^{-1}cm$-1; ESI-MS**:** *m/z* calcd 3559.6 [M]: 1187.1 $[M+3H]^{3+}$, 890.5 $[M+4H]^{4+}$, 712.7 $[M+5H]^{5+}$, 594.2 $[M+6H]^{6+}$, 509.4 $[M+7H]^{7+}$, 446.0 $[M+8H]^{8+}$, 396.5 $[M+9H]^{9+}$. **6-DOP-Coil:**

19.9%; t_r: 4.69 min (HPLC1); ε = 17989 $M^{-1}cm^{-1}$ at 270 nm; ESI-MS: *m/z* calcd 2583.0 [M]: 1291.5 $[M+2H]^{2+}$, 861.3 $[M+3H]^{3+}$, 646.3 $[M+4H]^{4+}$; **1-Hy-Coil:** 16.6%; t_r: 3.81 min (HPLC1); ε = 17989 $M^{-1}cm^{-1}$ at 270 nm; ESI-MS: *m/z* calcd 2498.02 [M]: 1249.5 $[M+2H]^{2+}$, 833.4 $[M+3H]^{3+}$, 625.3 $[M+4H]^{4+}$, 500.5 $[M+5H]^{5+}$, 417.3 $[M+6H]^{6+}$; **6-Hy-Coil:** 28.4%; t_r: 3.82 min (HPLC1); ε = 17989 $M^{-1}cm^{-1}$ at 270 nm; ESI-MS: *m/z* calcd 2498.02 [M]: 1249.5 $[M+2H]^{2+}$, 833.4 $[M+3H]^{3+}$, 625.3 $[M+4H]^{4+}$, 500.5 $[M+5H]^{5+}$, 417.3 $[M+6H]^{6+}$; **1-Hy-Coil(R):** 7.4%; t_r: 3.84 min (HPLC1); ε = 17989 $M^{-1}cm^{-1}$ at 270 nm; ESI-MS: *m/z* calcd 2638.1 [M]: 1319.6 $[M+2H]^{2+}$, 880.0 $[M+3H]^{3+}$, 660.3 $[M+4H]^{4+}$, 528.5 $[M+5H]^{5+}$, 440.5 $[M+6H]^{6+}$; **6-Hy-Coil(R):** 11.2%; t_r: 3.85 min (HPLC1); ε = 17989 $M^{-1}cm^{-1}$ at 270 nm; ESI-MS: *m/z* calcd 2638.1 [M]: 1319.6 $[M+2H]^{2+}$, 880.0 $[M+3H]^{3+}$, 660.3 $[M+4H]^{4+}$, 528.5 $[M+5H]^{5+}$, 440.5 $[M+6H]^{6+}$; **Hy-Random:** 48.5%; t_r: 3.15 min (HPLC1); ε = 2560 $M^{-1}cm^{-1}$ at 280 nm; ESI-MS: *m/z* calcd 1048.2 [M]: 1049.0 $[M+H]^{+}$, 524.7 $[M+2H]^{2+}$, 350.3 $[M+3H]^{3+}$;

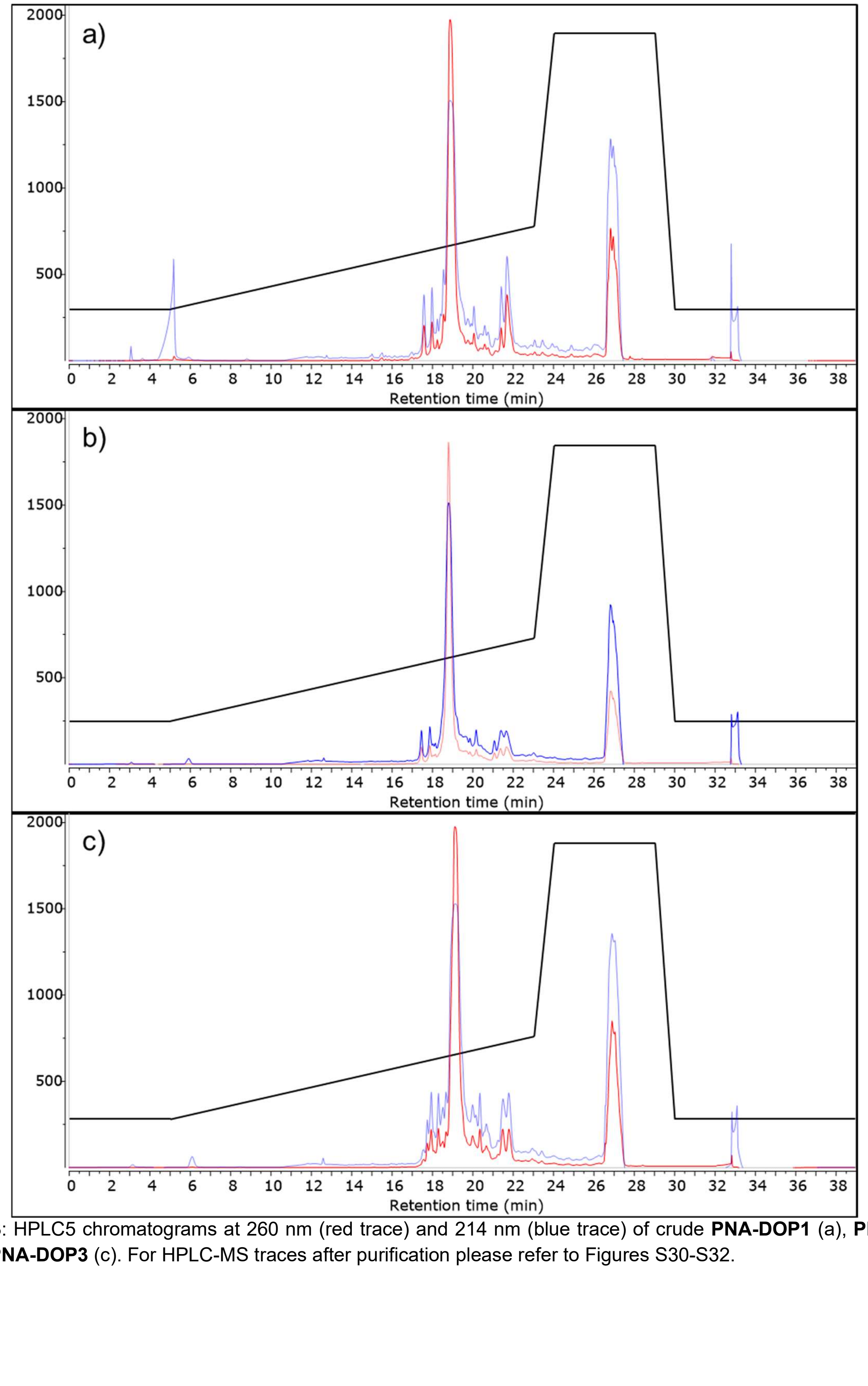

Figure S3: HPLC5 chromatograms at 260 nm (red trace) and 214 nm (blue trace) of crude **PNA-DOP1** (a), **PNA-DOP2** (b), and **PNA-DOP3** (c). For HPLC-MS traces after purification please refer to Figures S30-S32.

2. PNA:PNA ligation

In a typical experiment, 100 µL of buffered solutions (PBS pH 7.4) containing probes at 5 µM concentration (from a 100 µM stock solution), were prepared in a 0.5 mL Eppendorf and allowed to react overnight at 25 °C. The solutions were collected in the morning and analyzed via HPLC-UV, HPLC-MS, and USDS-PAGE.

2.1 Sequence and nucleophile selectivity of 2.5-DOP ligation

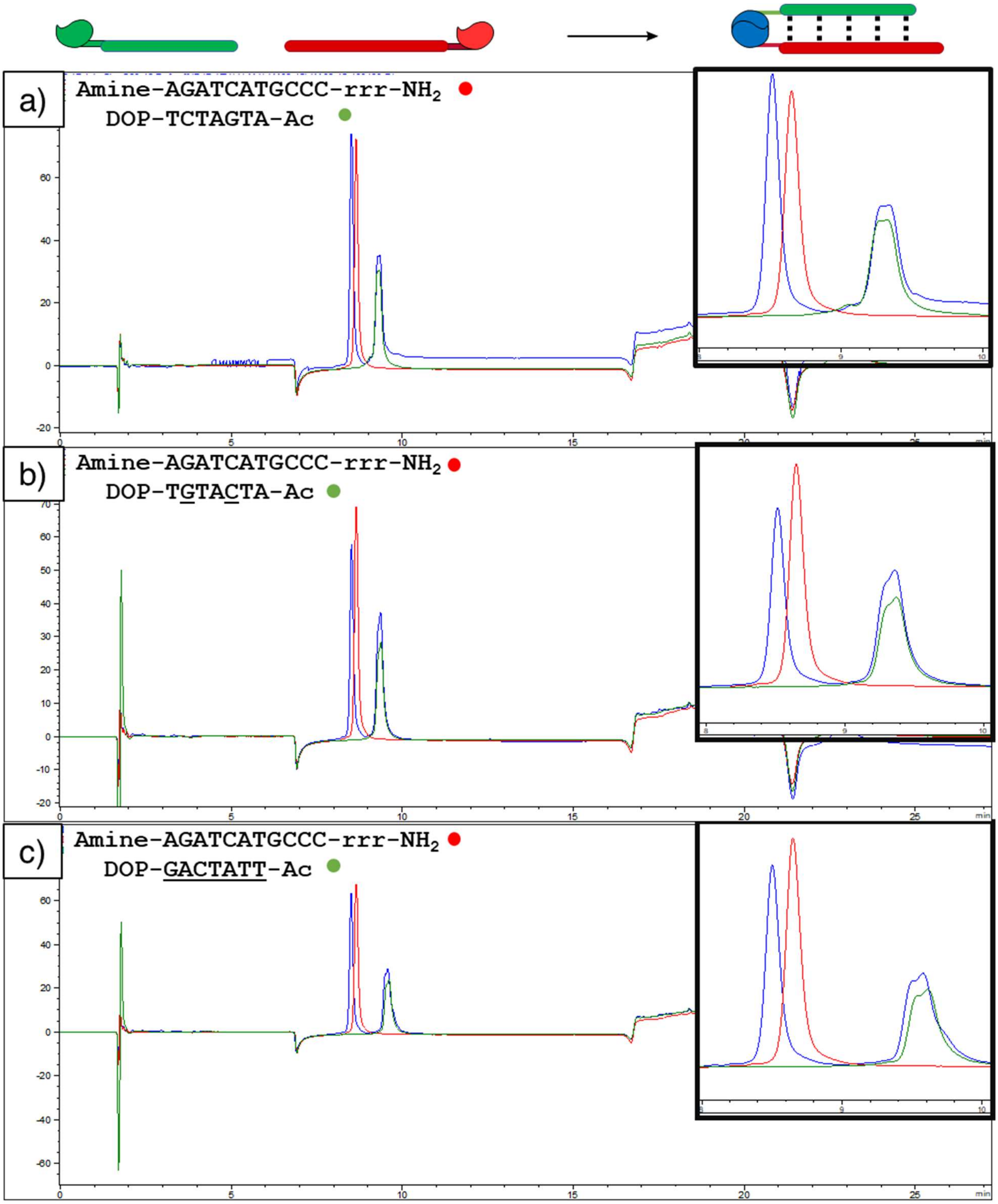

Figure S4: HPLC3 traces of the ligation experiments between amine-modified **PNA-Am1** and fully matched **PNA-DOP1** (a), double mismatched **PNA-DOP2** (b), and the scrambled **PNA-DOP3** (c). The inset shows a zoom of the probe region. Blue trace: ligation experiment; Red trace: nucleophilic PNA; Green trace: electrophilic PNA. Underlined bases indicate mismatches. A minor retention time difference was observed between references (red and green traces) and experiment (blue trace).

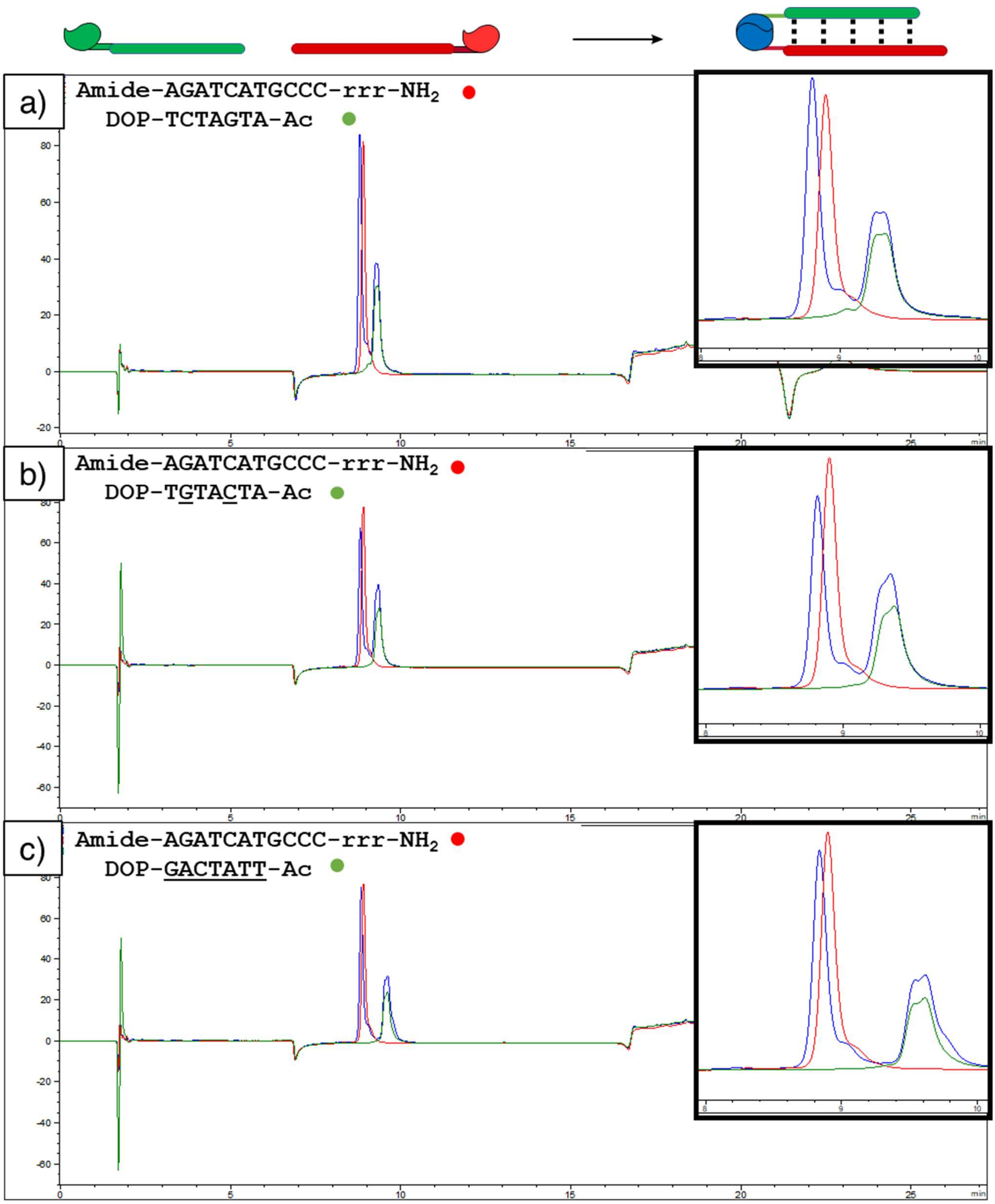

Figure S5: HPLC3 traces of the ligation experiments between amide-modified **PNA-Ac1** and fully matched **PNA-DOP1** (a), doubly mismatched **PNA-DOP2** (b), and scrambled **PNA-DOP3** (c). The inset shows a zoom of the probe region. Blue trace: ligation experiment; Red trace: nucleophilic PNA; Green trace: electrophilic PNA. Underlined bases indicate mismatches. A minor retention time difference was observed between references (red and green traces) and experiment (blue trace).

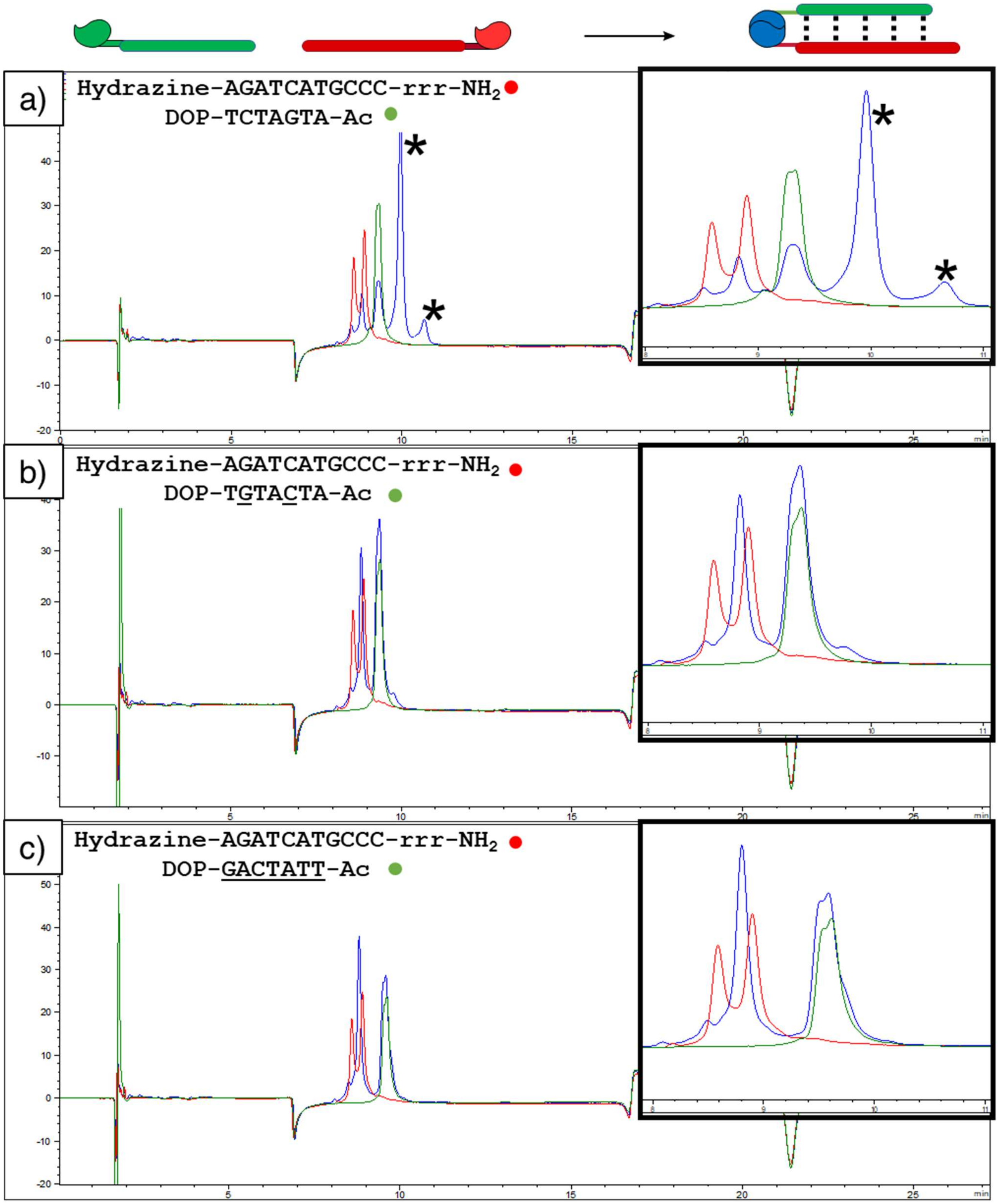

Figure S6: HPLC3 traces of the ligation experiments between hydrazine-modified **PNA-Hy1** and fully matched **PNA-DOP1** (a), doubly mismatched **PNA-DOP2** (b), and scrambled **PNA-DOP3** (c). The inset shows a zoom of the probe region. Blue trace: ligation experiment; Red trace: nucleophilic PNA; Green trace: electrophilic PNA. Underlined bases indicate mismatches; *: ligation product (all peaks, once isolated showed identical MW, this was then attributed to a difference in protonation state during HPLC analysis). Under the chromatographic conditions employed to monitor the reaction, the multiple peaks present in the **PNA-Hy1** reference were attributed to different protonation states of the probe, as reflected in the ligation product. A minor retention time difference was observed between references (red and green traces) and experiment (blue trace).

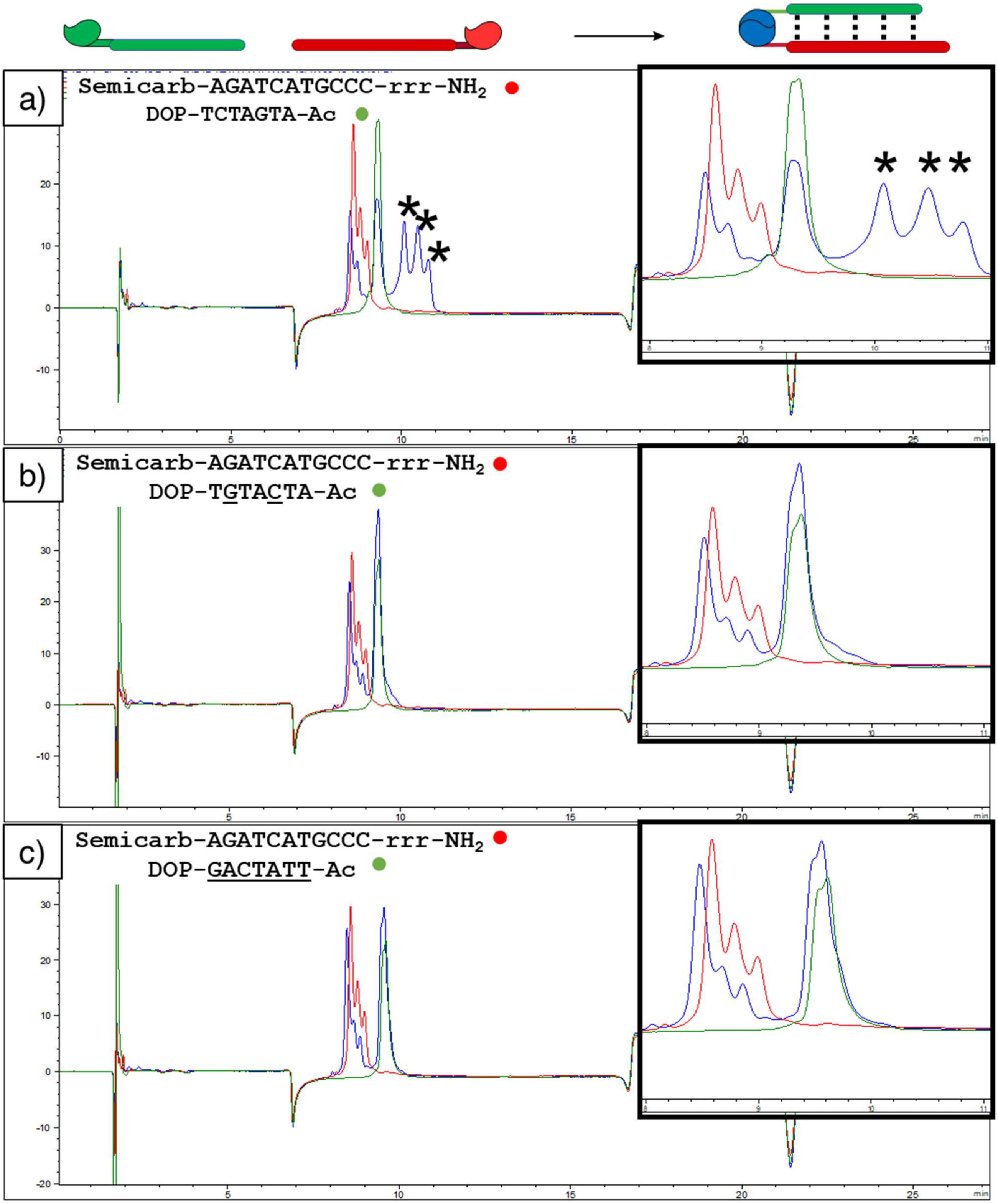

Figure S7: HPLC3 traces of the ligation experiments between semicarbazide-modified **PNA-Sc1** and fully matched **PNA-DOP1** (a), doubly mismatched **PNA-DOP2** (b), and scrambled **PNA-DOP3** (c). The inset shows a zoom of the probe region. Blue trace: ligation experiment; Red trace: nucleophilic PNA; Green trace: electrophilic PNA. Underlined bases indicate mismatches; *: ligation product (all peaks, once isolated showed identical MW, this was then attributed to a difference in protonation state during HPLC analysis or to hydrated forms). Under the chromatographic conditions employed to monitor the reaction, the multiple peaks present in the **PNA-Sc1** reference were attributed to different protonation states of the probe, as reflected in the ligation product. A minor retention time difference was observed between references (red and green traces) and experiment (blue trace).

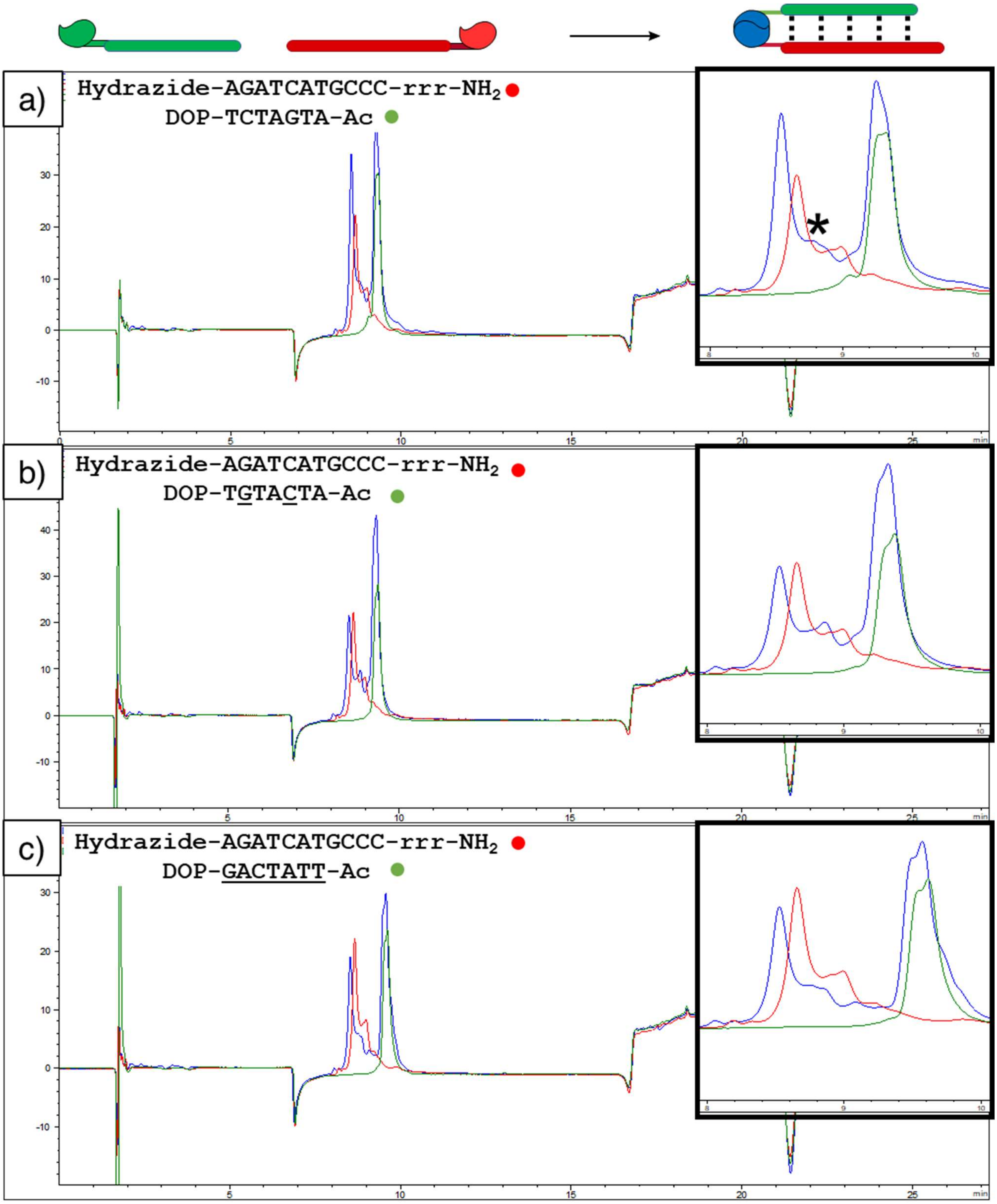

Figure S8: HPLC3 traces of the ligation experiments between hydrazide-modified **PNA-Hd1** and fully matched **PNA-DOP1** (a), doubly mismatched **PNA-DOP2** (b), and scrambled **PNA-DOP3** (c). The inset shows a zoom of the probe region. Blue trace: ligation experiment; Red trace: nucleophilic PNA; Green trace: electrophilic PNA. Underlined bases indicate mismatches; *: possible ligation product. A minor retention time difference was observed between references (red and green traces) and experiment (blue trace).

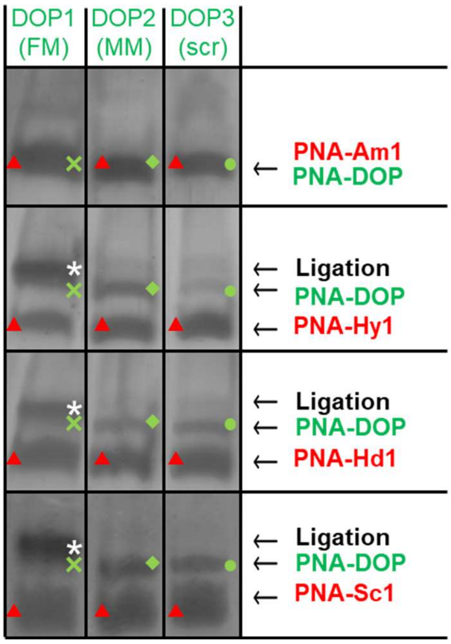

Figure S9: USDS-PAGE analysis of the ligation experiments of the different DOP-modified PNAs in presence of amine-modified **PNA-A1**, hydrazine-modified **PNA-H1**, hydrazide-modified **PNA-Hd1**, and semicarbazide-modified **PNA-Sc1**. For clarity, the positions of the electrophilic PNAs (green symbols), nucleophilic PNAs (red triangles), and ligation products (white star) are marked.

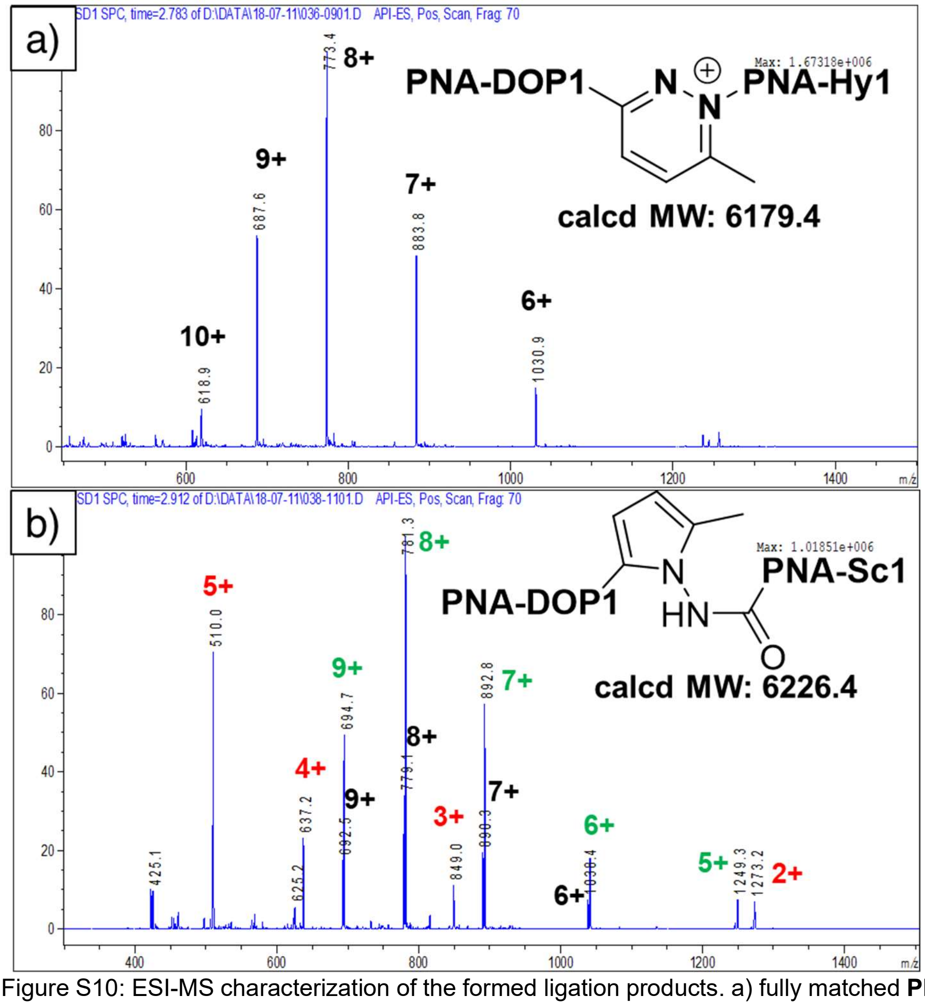

Figure S10: ESI-MS characterization of the formed ligation products. a) fully matched **PNA-DOP1** and hydrazine-modified PNA-H1; b) **PNA-DOP1** + and semicarbazide-modified **PNA-S1.** The green and red multicharged signals correspond to the hydrated adduct and the product with scission of the N-N bond, respectively.

2.2 2,5-DOP ligation at higher concentration

50 µL of buffered solutions (PBS pH 7.4) containing probes at 200 µM concentration, were prepared in a 0.2 mL Eppendorf and allowed to react over a period of 5 days. The solutions were finally diluted to 5 µM and injected in HPLC-MS. In this experiment, longer PNA probes were used (*vide infra*).

As the probe concentration employed in testing the sequence and nucleophile selectivity of the DOP-ligation was relatively low, we wanted to explore if it was possible to obtain similar results in terms of selectivity at higher probe concentrations. As confirmed by the ESI-MS analyses shown in Figure S12-Figure S13, the formation of the ligation product only occurs in presence of hydrazine-containing PNA probe in presence of the corresponding fully matching PNA-DOP.
As can be noted from the aforementioned ESI-MS spectra, nucleophilic probes and products may show a +16 Da difference from the expected MW. This can be ascribed to the oxidation of the biotin tag induced by the presence of TAMRA as previously described for this type of probes and for other fluorescent biotinylated molecules.[1,4]

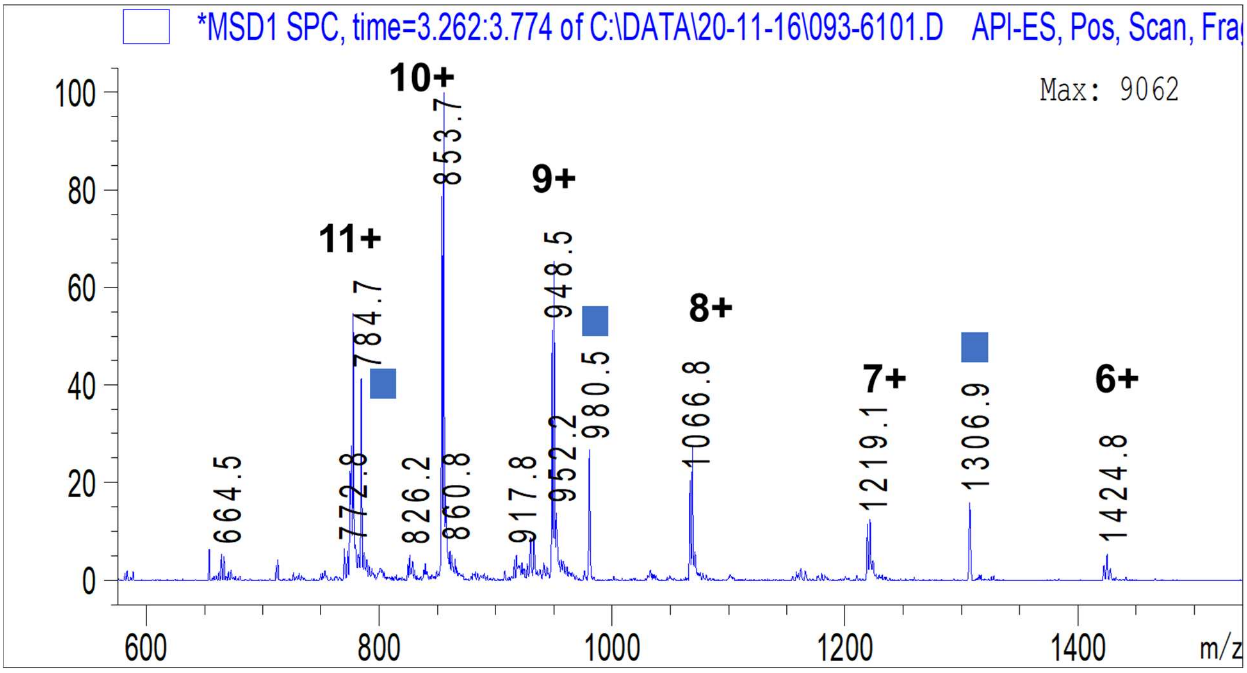

Figure S11: ESI-MS characterization of the reaction crude after performing the reaction between **PNA-DOP5** (3919 Da, fully matching probe) and hydrazine-modified **PNA-Hy2** (4646 Da, as in Figure S13b) at high concentration; blue squares indicate starting material signals. Ligation product MW: 8527 Da.

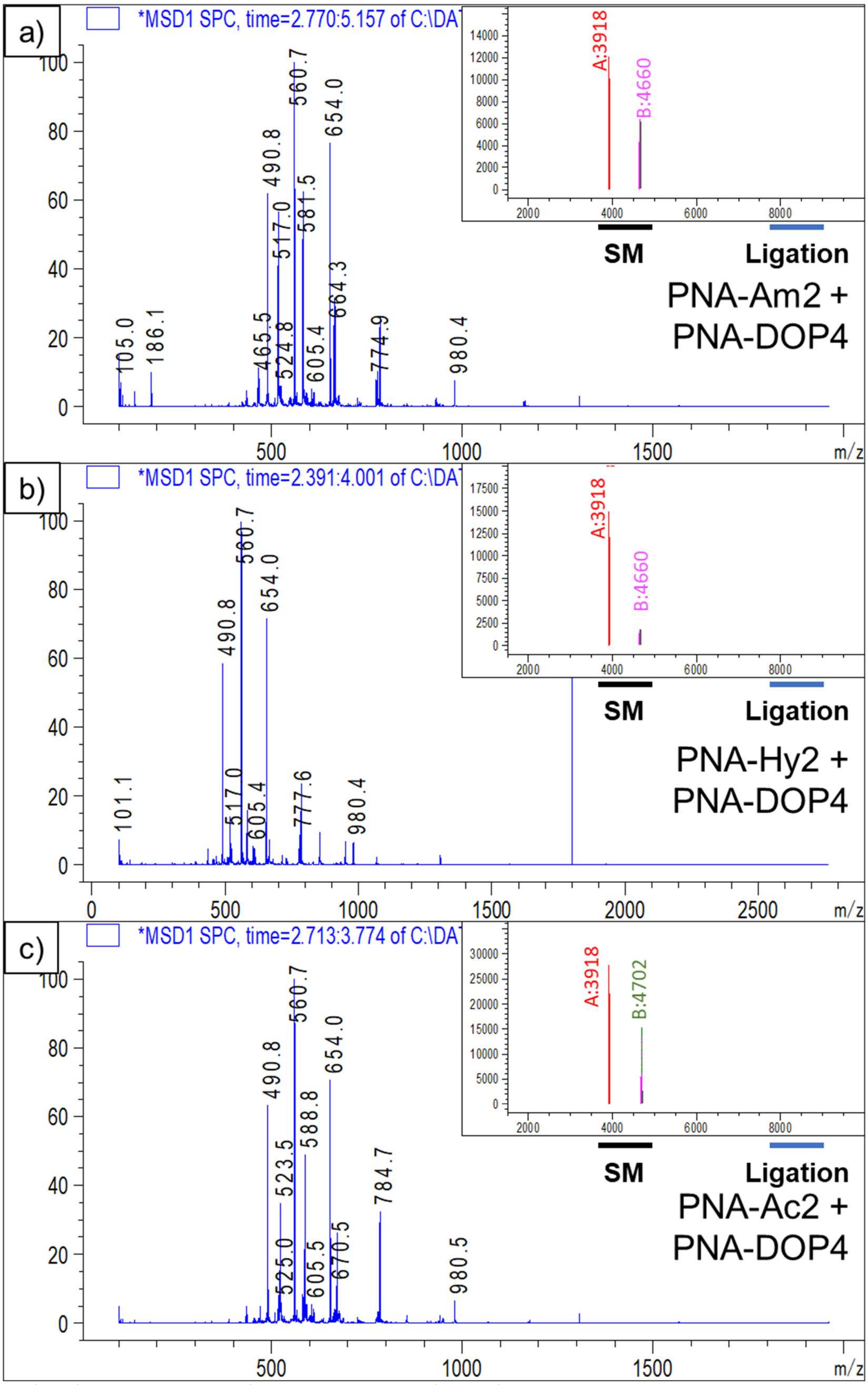

Figure S12: ESI-MS characterization of the reaction crude after performing the reaction at high concentration in presence of scrambled **PNA-DOP4**. a) amine-containing **PNA-Am2**; b) hydrazine-containing **PNA-Hy2**; c) amide-containing **PNA-Ac2**. MW: **PNA-DOP4**: 3919 Da, **PNA-Am2**: 4645 Da, **PNA-Hy2**: 4646 Da, **PNA-Ac2**: 4687 Da, ligation product: 8527 Da.

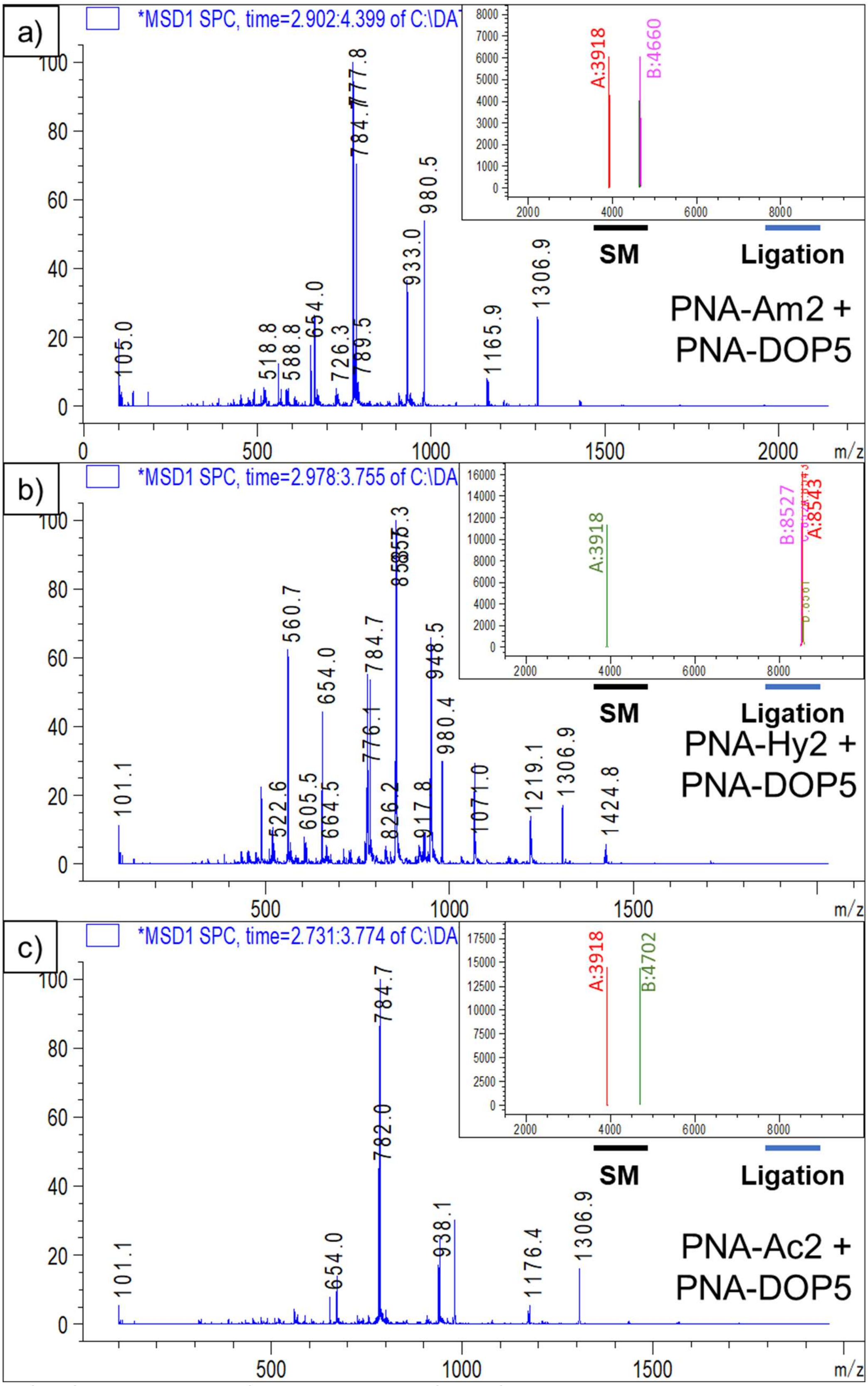

Figure S13: ESI-MS characterization of the reaction crude after performing the reaction at high concentration in presence of fully matching **PNA-DOP5**. a) amine-containing **PNA-Am2**; b) hydrazine-containing **PNA-Hy2**; c) amide-containing **PNA-Ac2**. MW: **PNA-DOP5**: 3919 Da, **PNA-Am2**: 4645 Da, **PNA-Hy2**: 4646 Da, **PNA-Ac2**: 4687 Da, ligation product: 8527 Da.

2.3 2,5-DOP ligation in presence of a lysine terminated PNA

100 µL of buffered solutions (PBS pH 7.4) containing probes at 5 µM concentration (from a 100 µM stock solution), were prepared in a 0.5 mL Eppendorf and allowed to react overnight at 25 °C. The solutions were collected in the morning and analyzed via HPLC-UV.
In this experiment both PNA:PNA and templated PNA_2:D(R)NA geometries were tested.

Probes bearing a terminal lysine were tested to evaluate the influence on the reaction outcome of probes bearing various (unreactive) nucleophiles. As can be observed from the following HPLC-UV traces, no new peaks were formed in any of the tested geometries.

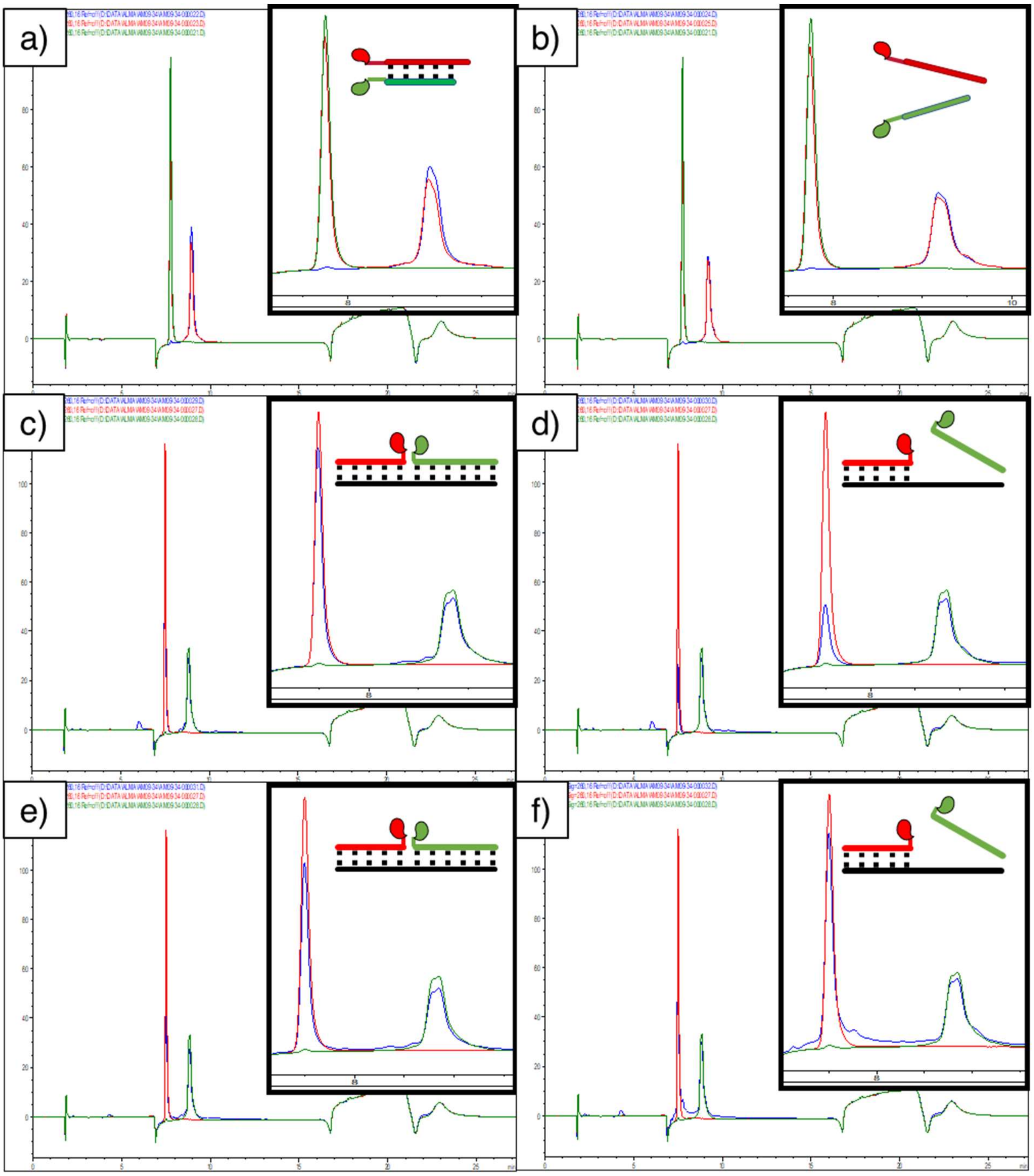

Figure S14: HPLC3 traces of the ligation experiments performed in presence of **PNA-K1.** (a) with **PNA-DOP1**, (b) with **PNA-DOP3**, (c) with **PNA-DOP3** and **DNA-1**, (d) with **PNA-DOP3** and **DNA-2**, (e) with **PNA-DOP3** and **RNA-1**, (f) with **PNA-DOP3** and **RNA-2**. The inset shows a zoom of the probe region. Blue trace: ligation experiment; Red trace: nucleophilic PNA; Green trace: electrophilic PNA.

2.4 Effect of oxidizing conditions on the ligation reaction

In a typical experiment, 100 µL of buffered solutions (PBS pH 7.4) containing probes at 5 µM concentration (from a 100 µM stock solution), were prepared in a 1.5 mL Eppendorf and allowed to hybridize at 25°C for 15 minutes before the addition of Rhodamine B at 5 µM final concentration. The lamp (Illuminator EK-1 lamps, 100W halogen lamp LE.5210, equipped with Euromex LE.5214 dual-arm light conductor) is then placed on top of the Eppendorf vial for the duration of the experiment. The reaction mixture was sampled at different irradiation times, and the samples left to react overnight at 25°C. Results were analyzed via HPLC-UV experiments

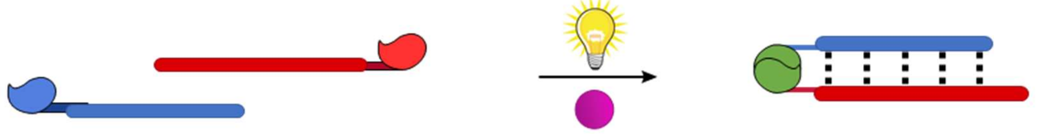

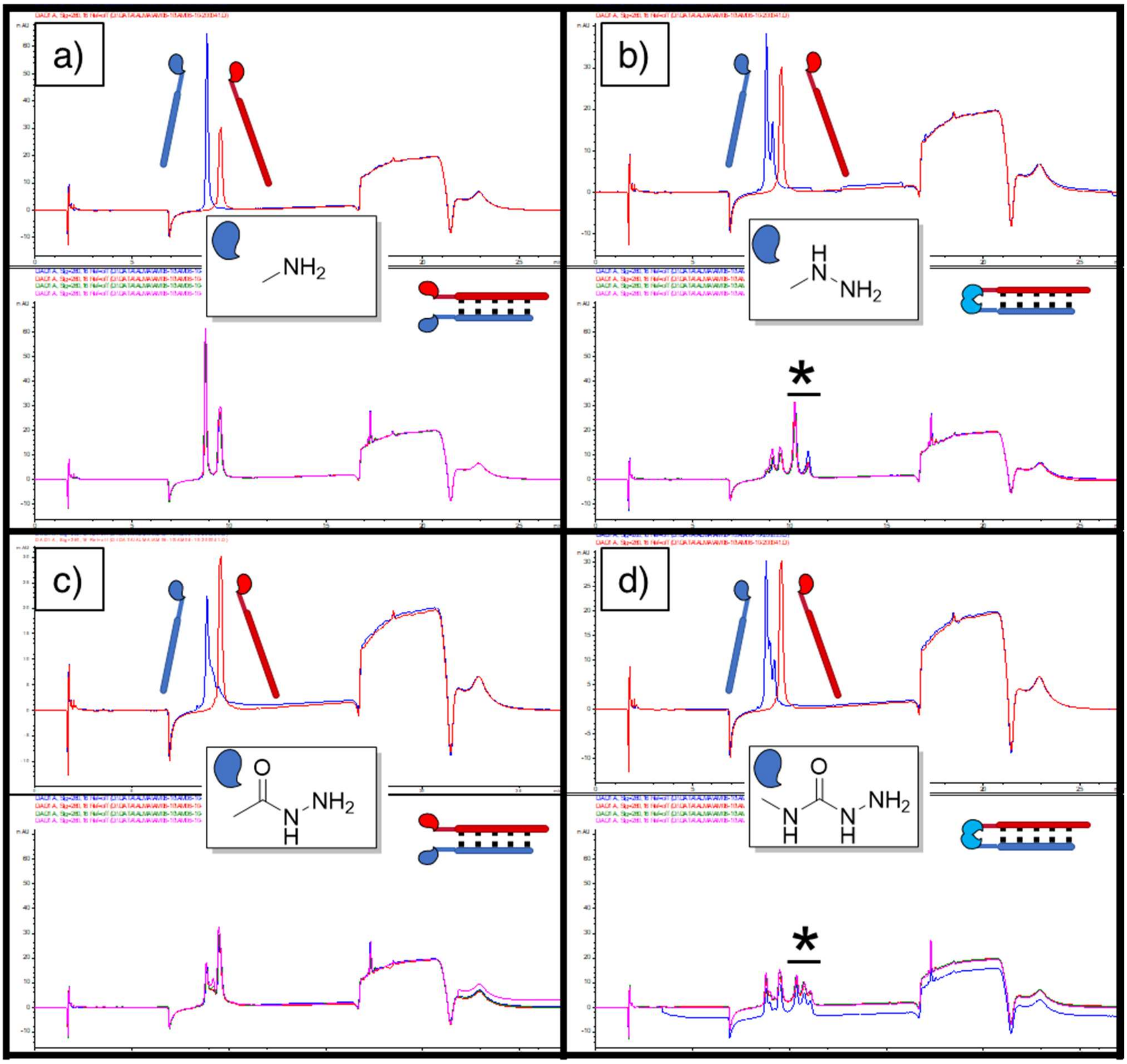

Figure S15: HPLC3 traces of the ligation experiments between different nucleophilic PNAs (a: **PNA-Am1**; b: **PNA-Hy1**; c: **PNA-Hd1**; d: **PNA-Sc1**) and fully matched **PNA-DOP1**. In each panel, the top traces show the reference PNAs, while the bottom traces show the effect of different light irradiation times (0', 15', 30', and 60') in presence of 5µM rhodamine B. *: newly formed peaks.

2.5 2,5-DOP ligation in cell lysate

60 µL of buffered solutions (PBS pH 7.4) or 60 µL of cell lysate (4 million SK-MEL-28 cells/mL) containing

probes at 5 µM concentration (from a 100 µM stock solution), were prepared in a 0.2 mL Eppendorf and allowed to react overnight at 25 °C. The solutions were collected in the morning and analyzed via USDS-PAGE.

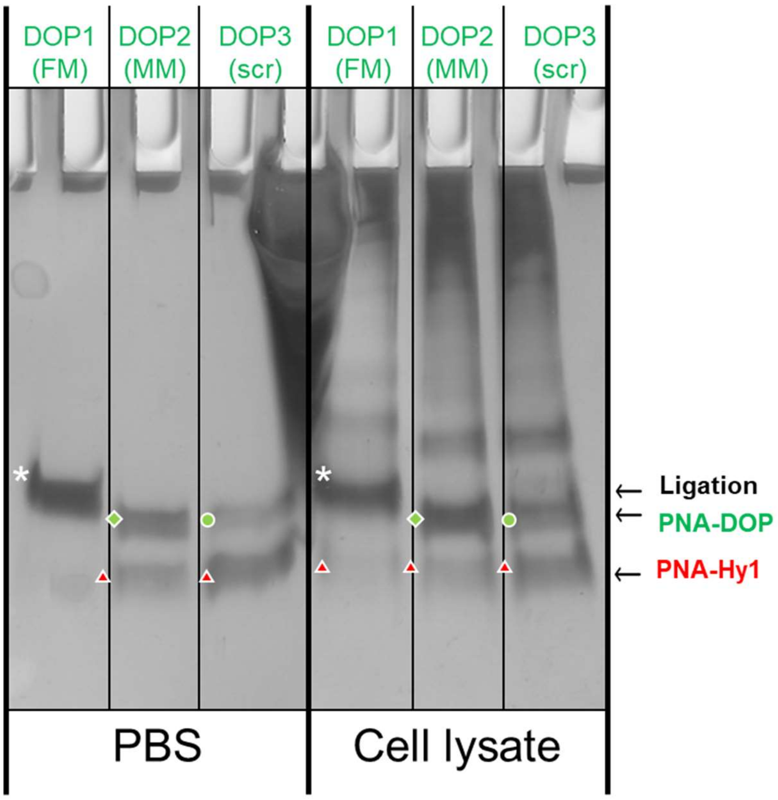

Figure S16: USDS-PAGE analysis of the ligation experiments of the different DOP-modified PNAs in presence of hydrazine-modified **PNA-H1** in PBS or cell lysate. For clarity, the positions of the electrophilic PNAs (green symbols), nucleophilic PNA (red triangles), and ligation product (white star) are marked.

3. Structural investigation on small molecules

General protocol: To an ice-cold solution of 100 mg hexan-2,5-dione (876 µmol) in 200 µL DMF, a solution of the desired α-effect nucleophile (876 nmol in 200 µL DMF, 1 eq) was added, to obtain a concentration after mixing of ~2M. The resulting solution was left to react at room temperature over the 3 days. Target compounds were then isolated through RP-HPLC.

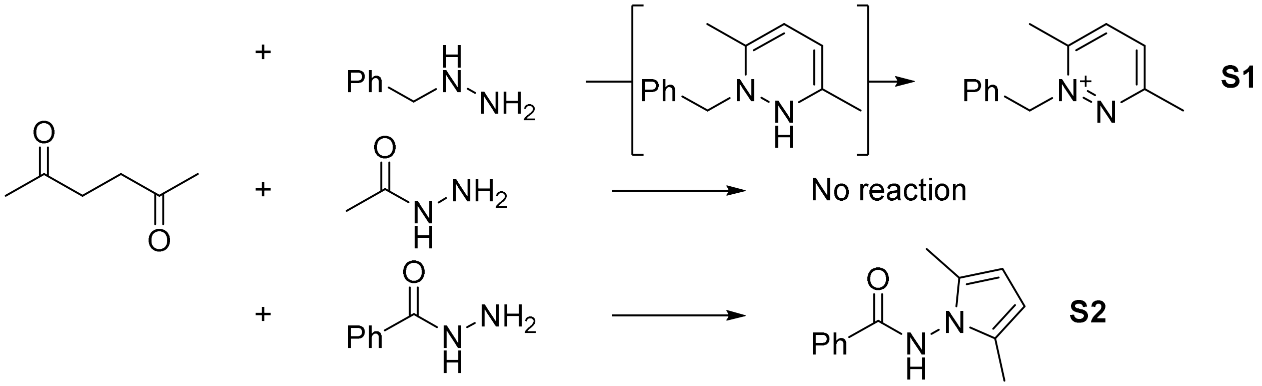

Scheme S1: Reactions performed with hexan-2,5-dione as starting material and different α-effect nucleophiles and the respective products.

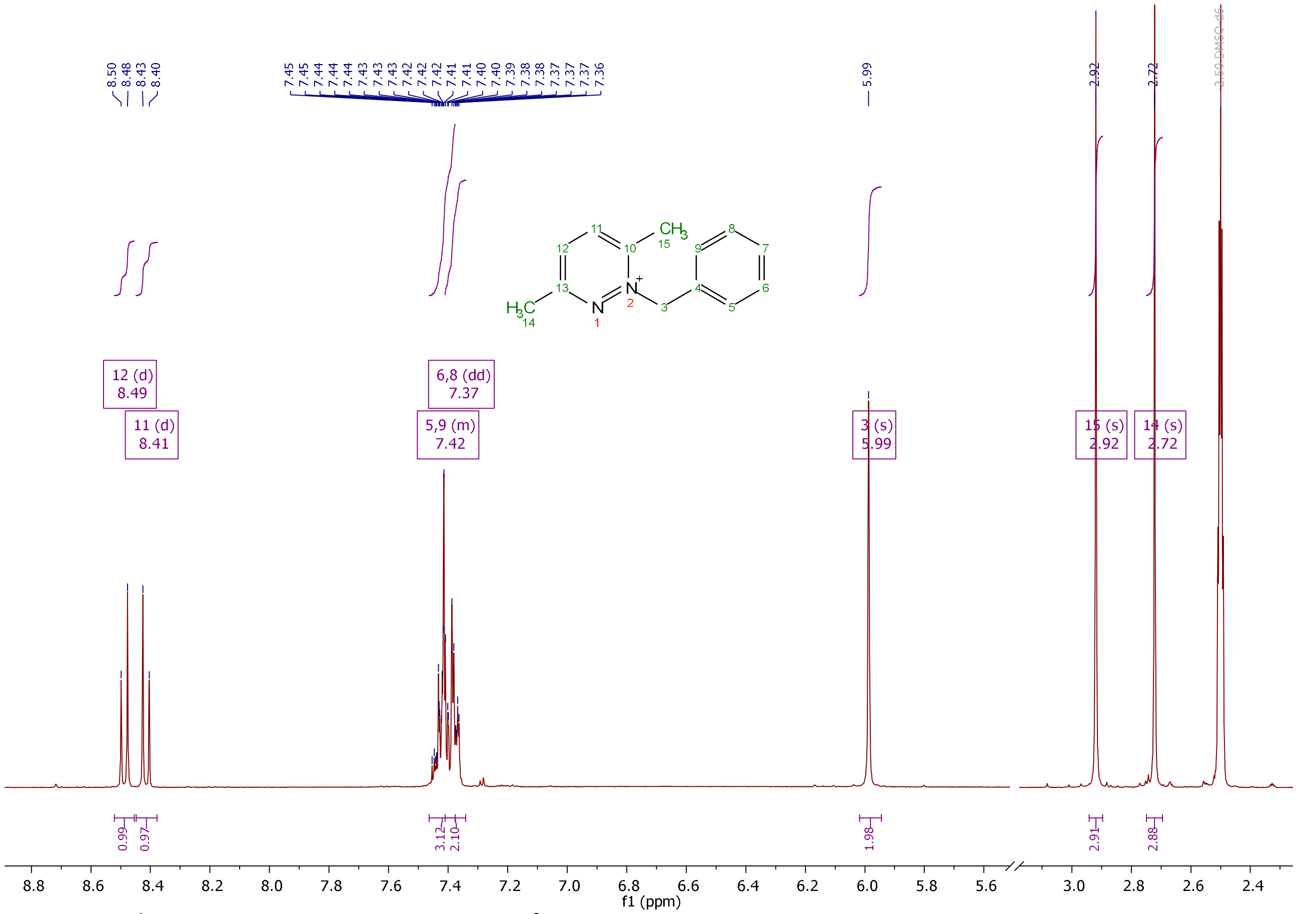

Figure S17: ^{1}H-NMR of compound **S1** in DMSO-d^6.

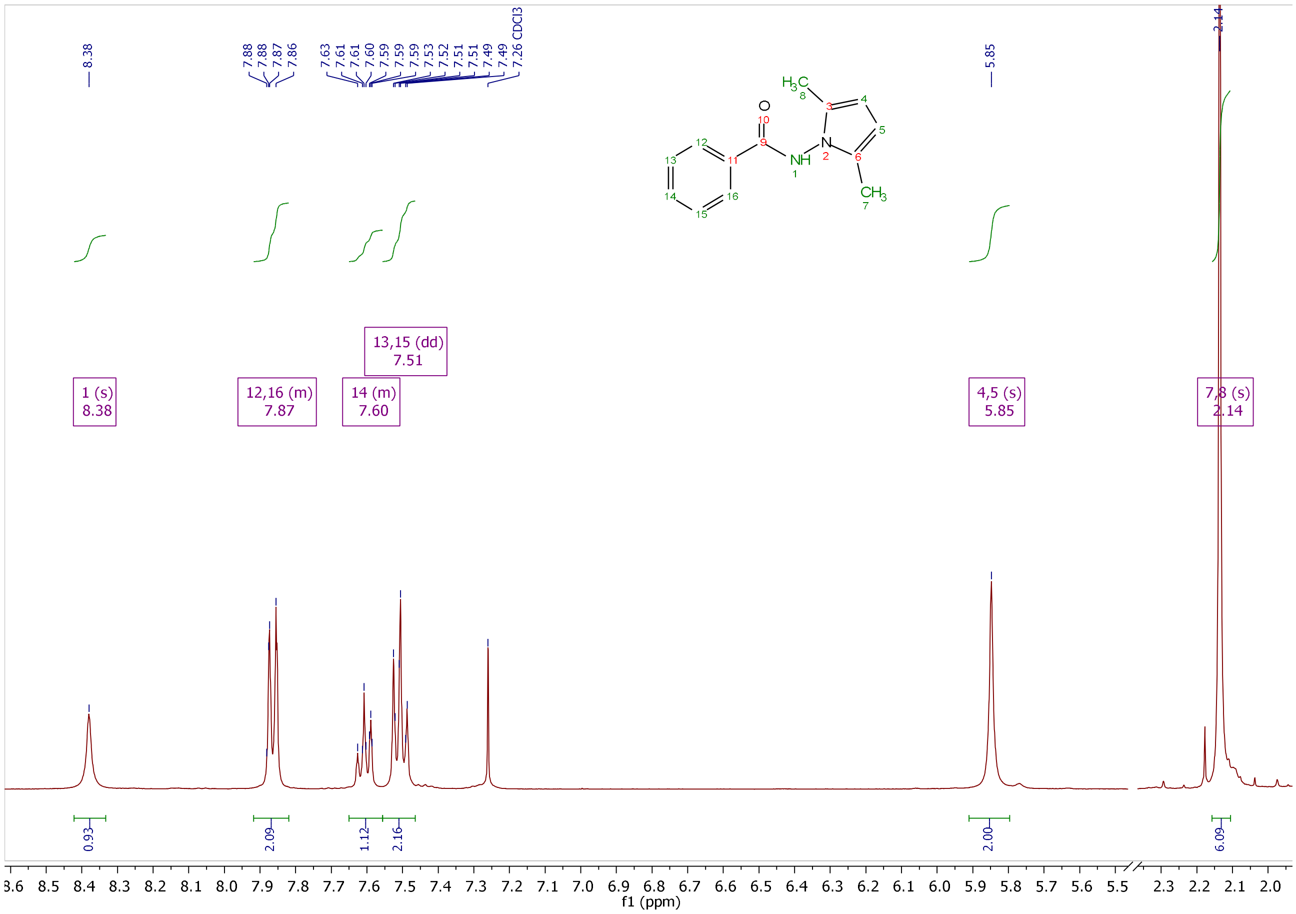

Figure S18: ^{1}H-NMR of compound **S2** in $CDCl_3$.

4. Stability evaluation of the pyridazinium ligation product

In a 1.5 mL Eppendorf, 500 µL of buffered solutions at different pH values were prepared (PBS buffer, pH 7.4 or borate-citrate-phosphate saline buffer), containing compound **S1** at a final concentration of 100 µM, added from a 1 mM stock solution. The mixtures were shaken at 37 °C for 7 days or at 90°C for 24 h. 50 µL aliquots were sampled at different time points and analyzed via HPLC-UV analysis.

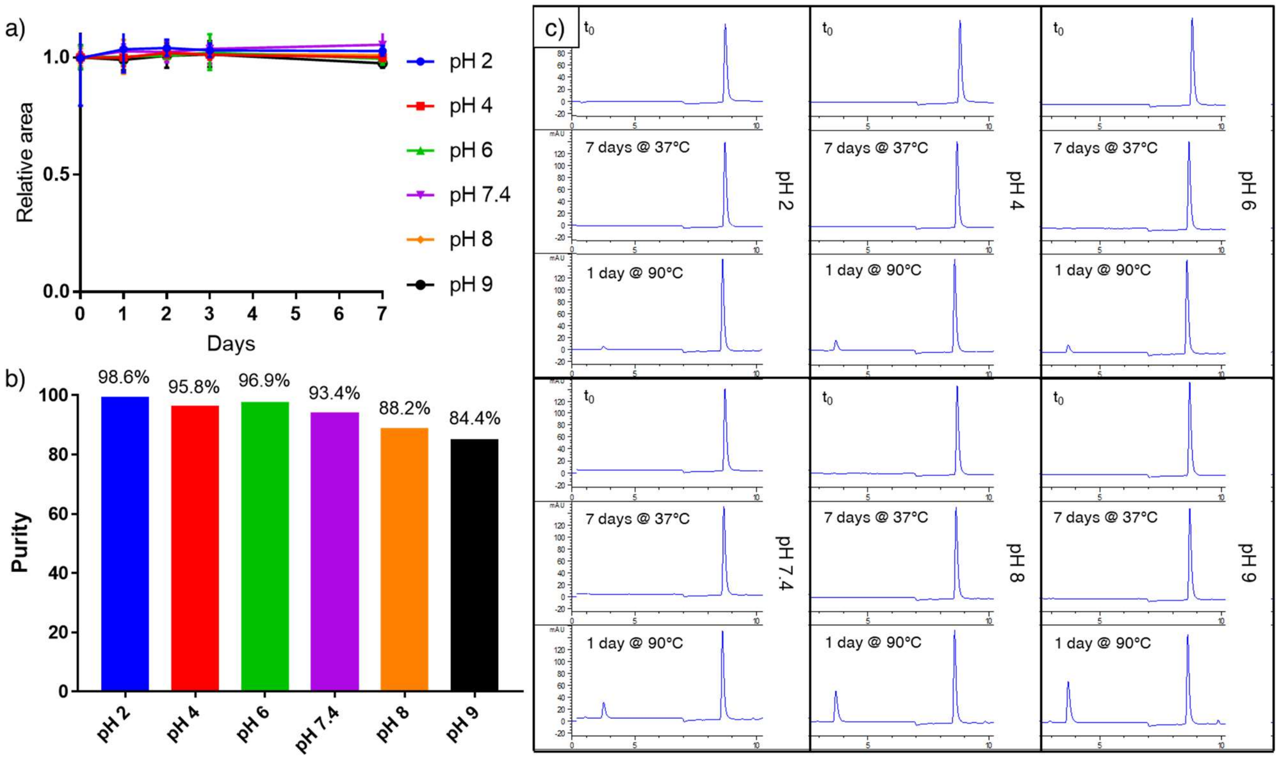

Figure S19: a) Relative HPLC-UV peak areas of **S1** as a function of time for the incubation at 37°C under different pH values; b) sample purity at 214 nm after heating at 90°C for 24h, under different pH values; c) comparison of the HPLC-UV traces at 260 nm obtained after degradation of **S1** under different conditions.

5. DNA templated PNA-PNA ligation

In a typical experiment, 100 µL of buffered solutions (PBS pH 7.4) containing all probes at 5 µM concentration (from a 100 µM stock solution), were prepared in a 0.5 mL Eppendorf. The complex between DNA and the nucleophilic PNA was allowed to equilibrate for 5 minutes before the final addition of DOP-PNA probe, and the mixtures were allowed to react overnight at 25 °C. The solutions were collected in the morning and analyzed via HPLC-UV, HPLC-MS, and USDS-PAGE.

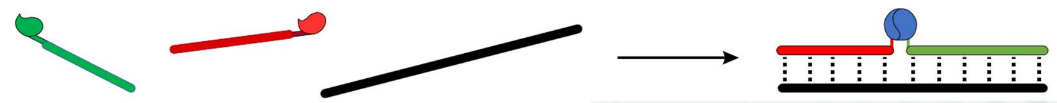

**Lane 1**	Ref DNA-1	-
**Lane 2**	Ref PNA-Hy1 + DNA-1	-
**Lane 3**	Ref PNA-DOP2 + DNA-1	-
**Lane 4**	Ref PNA-Hy1 + PNA-DOP2	Mismatched PNA sequences
**Lane 5**	PNA-Hy1 + PNA-DOP2	Without DNA
**Lane 6**	PNA-Hy1 + PNA-DOP2 + DNA-0T	With FM-DNA
**Lane 7**	PNA-Hy1 + PNA-DOP2 + DNA-S4	With 2MM-DNA
**Lane 8**	PNA-Hy1 + PNA-DOP2 + DNA-6	With 3MM-DNA
**Lane 9**	Ref PNA-Hy1 + DNA-3	Fully random PNA sequence
**Lane 10**	PNA-Hy1 + PNA-DOP3	Without DNA
**Lane 11**	PNA-Hy1 + PNA-DOP3 + DNA-S1	With FM-DNA
**Lane 12**	PNA-Hy1 + PNA-DOP3 + DNA-S2	With 2MM-DNA
**Lane 13**	PNA-Hy1 + PNA-DOP3 + DNA-S3	With 3MM-DNA
**Lane 14**	Color	-

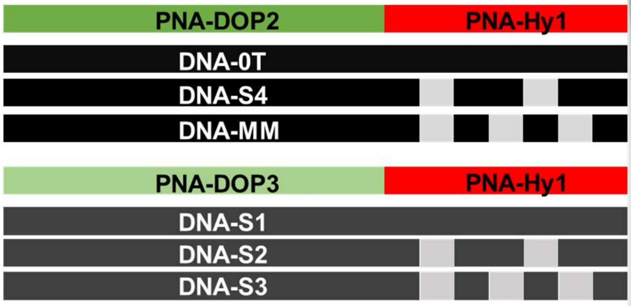

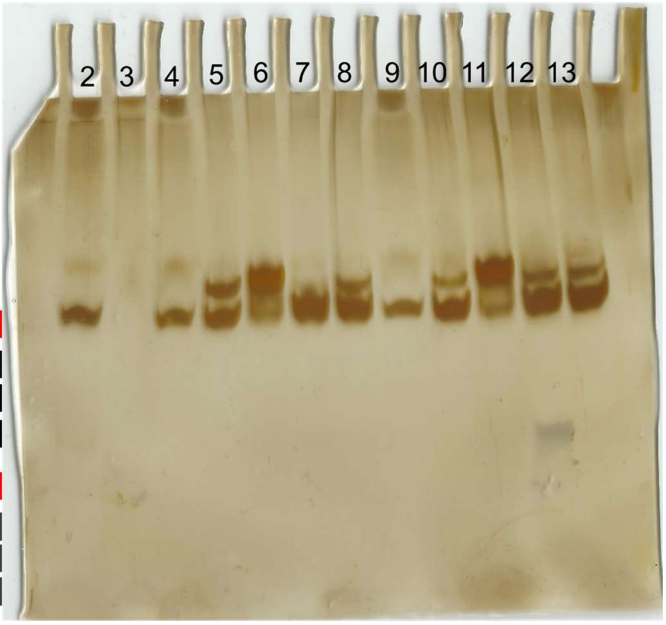

Figure S20: USDS-PAGE analysis of the ligation experiments between **PNA-Hy1** and **PNA-DOP2** or **PNA-DOP3**, in presence of different DNA templates. The cartoon on the lower left side of the picture shows the position of the mismatches (in gray) with respect to the DNA sequences.

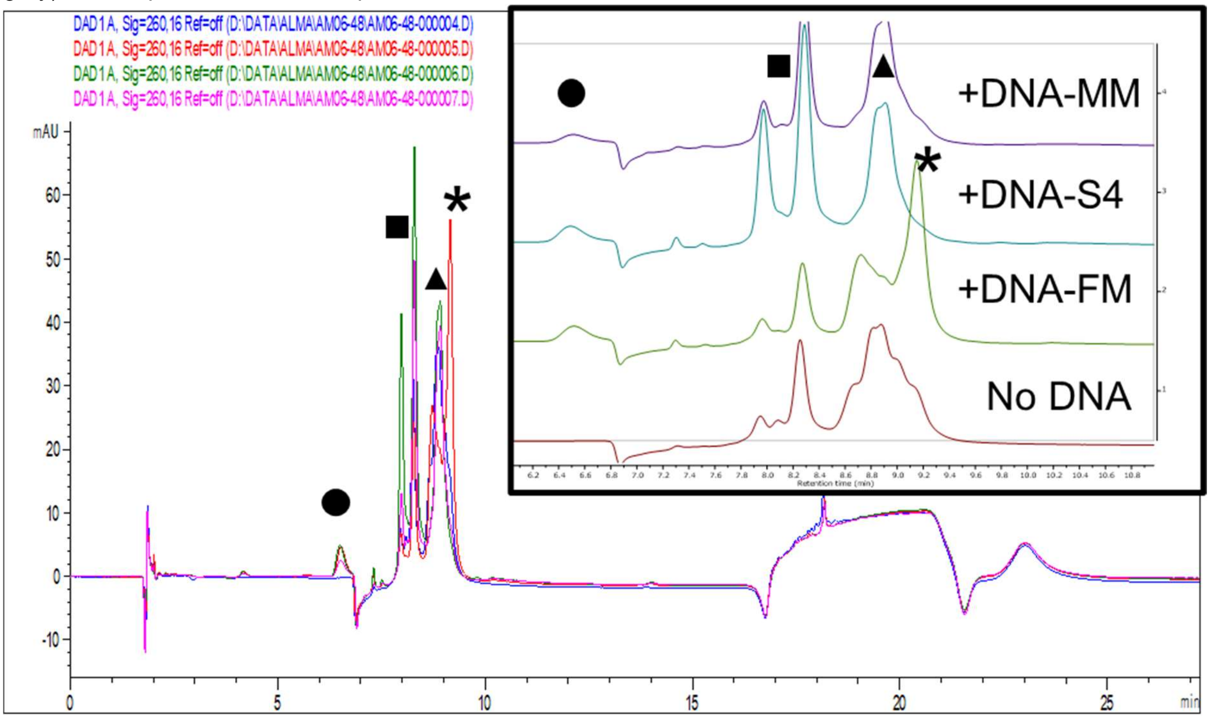

Figure S21: HPLC3 traces of the ligation experiments between **PNA-Hy1** (■) and **PNA-DOP2** (▲) in presence of different templating DNA strands (●). The inset shows a zoom of the probe region, from bottom to top: no DNA, **DNA-0T** (FM), **DNA-S4** (2MM), and **DNA-MM** (3MM). Product peak is identified with an asterisk (*).

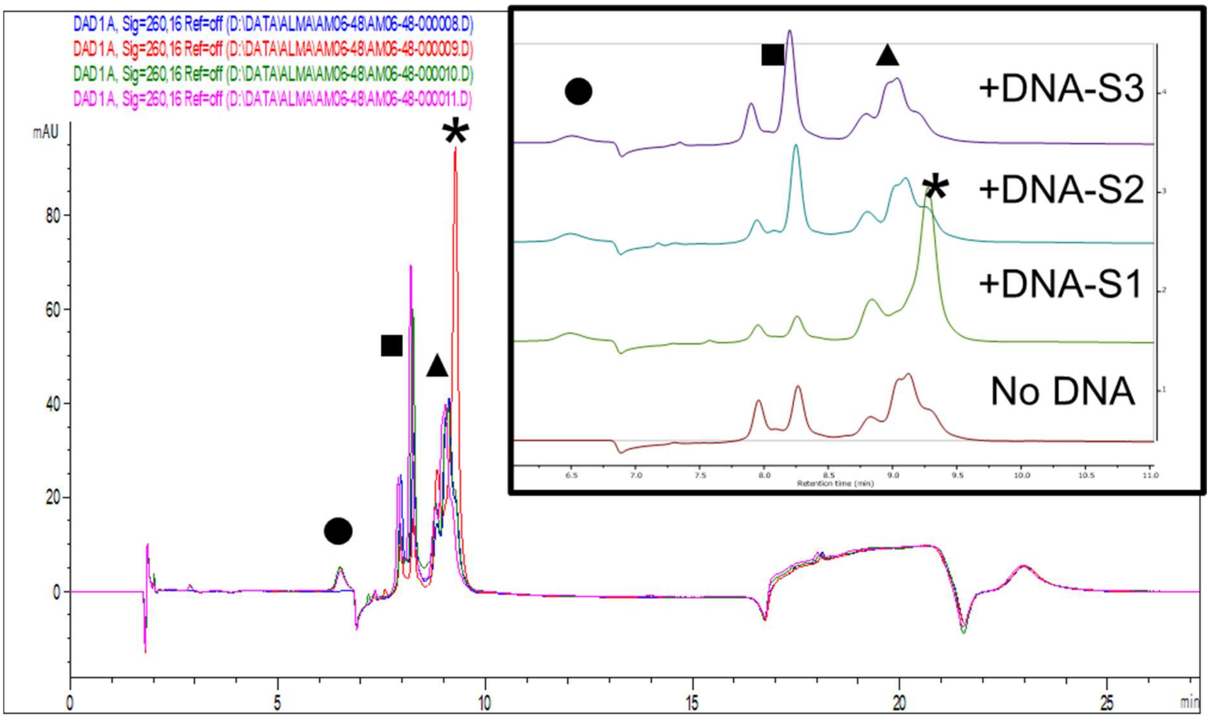

Figure S22: HPLC3 traces of the ligation experiments between **PNA-Hy1** (■) and **PNA-DOP3** (▲) in presence of different templating DNA strands (●). The inset shows a zoom of the probe region, from bottom to top: no DNA, **DNA-S1** (FM), **DNA-S2** (2MM), and **DNA-S3** (3MM). Product peak is identified with an asterisk (*).

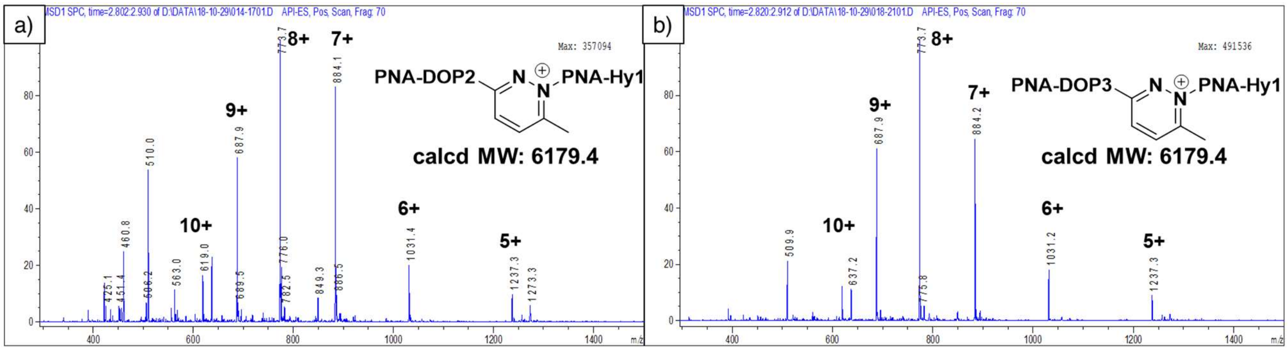

Figure S23: ESI-MS characterization of the formed products. a) **PNA-DOP2** (2546 Da) + **PNA-Hy1** (3674 Da) in presence of template **DNA-FM**; b) **PNA-DOP3** + **PNA-Hy1** in presence of template **DNA-S1**. Ligation Expected MW: 6179 Da**.**

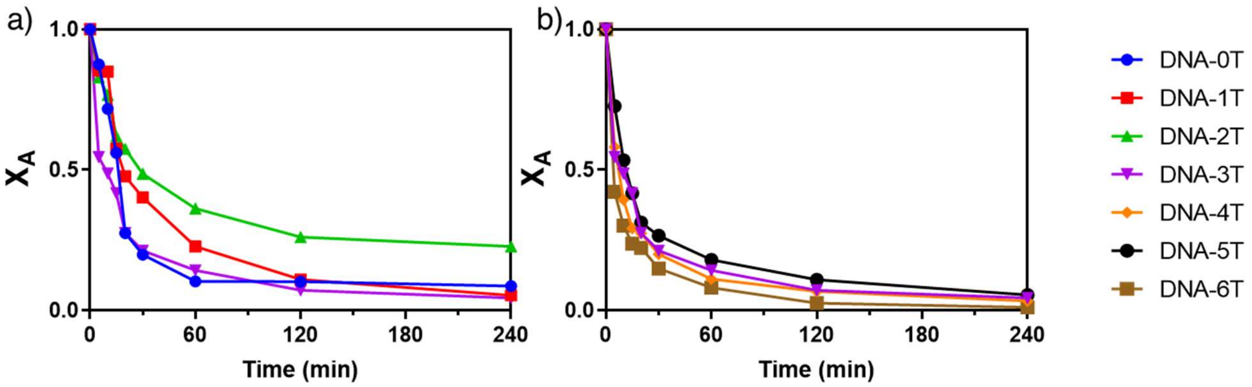

Figure S24: Consumption profiles of **PNA-Hy1'** in presence of 1.1 eq of **PNA-DOP2** and different **DNA-nT** strands (a: n=0-3, b: n= 3-6).

6. Surface ligation

6.1 96-well plate functionalization

Pierce Amine-binding, maleic anhydride activated, 96-well plate strips (Thermo-Fisher) were used as solid support. Functionalization was performed by adding 100 µL of a 500 nM PNA buffered solution (100 mM carbonate buffer, pH 9.0) containing 20% acetonitrile and 0.001% SDS. The plates were shaken overnight at 300 RPM (orbital shaking), at room temperature. Unreacted sites were quenched using a 50 mM ethanolamine solution in 100 mM carbonate buffer pH 9.0 (300 µL, 260 RPM, 2h). Finally, the surfaces were washed with a 0.01% SDS solution (2 minutes, twice), 0.001% SDS solution (2 minutes, twice), and milliQ water (mQ). The 96 well plates were then dried and stored over $CaCl_2$.

6.2 Surface template ligation in 96-well plate

Oligonucleotides and PNA solutions were freshly prepared in PBS pH 7.4 supplemented with 0.001% SDS (PBS-S) from a 10 µM stock solutions in mQ. Surfaces were pre-wetted for 30 minutes with a 0.001% SDS solution. Then, 50 µL of oligonucleotide and 50 µL of 1 µM PNA solutions were allowed to react overnight at 40°C. Wells were washed with a mQ/MeCN 1:1 + 0.1% TFA solution (4x 5minutes, 45°C) before the quantification of the attached biotin, using 100 µL of 20 ng/mL Pierce High Sensitivity NeutrAvidin-HRP-conjugate (Thermo Scientific) and 1-step Ultra TMB-ELISA (Thermo Scientific) as reagent solution. Final readout of the oxidized TMB was performed by monitoring the absorption at 450 nm after quenching the reaction with 2M H_2SO_4.

6.3 Microarray slide functionalization

NHS-active ester XL-CX slides (Xantec, Germany) were used as solid support. Functionalization was performed spotting 0.3 µL of a 1 µM PNA solution in 100 mM carbonate buffer pH 9.0 containing 30% glycol and 0.0001% SDS. Functionalization was allowed overnight in a humid chamber (75% relative humidity) and remaining active sites were quenched for 4h using a 6% ethanolamine solution in 100 mM carbonate buffer pH 9.0. Finally, surfaces were washed with a 0.01% SDS solution (10 minutes, twice), 0.001% SDS solution (10 minutes, twice), and mQ. Slides were dried with a stream of clean air and stored over $CaCl_2$. All steps were performed away from direct light.

6.4 Surface template ligation on microarray slides

Oligonucleotide and PNA solutions were freshly prepared in PBS-S from a 10 µM stock solution in mQ. Surfaces were pre-wetted for 30 minutes with a 0.001% SDS solution and then dried with a stream of clean air, before the application of the desired mask (in a typical experiment a 16 well mask is employed). 50 µL of oligonucleotide and 50 µL of 100 nM PNA solutions were added and allowed to react overnight at 40°C. Slides were washed in PBS pH 7.4, supplemented with 0.05% TWEEN-20 (2x 10 minutes, 50°C) and mQ (1 minute, r.t.). Slides were then dried with a stream of clean air before image acquisition. All steps were performed away from direct light.

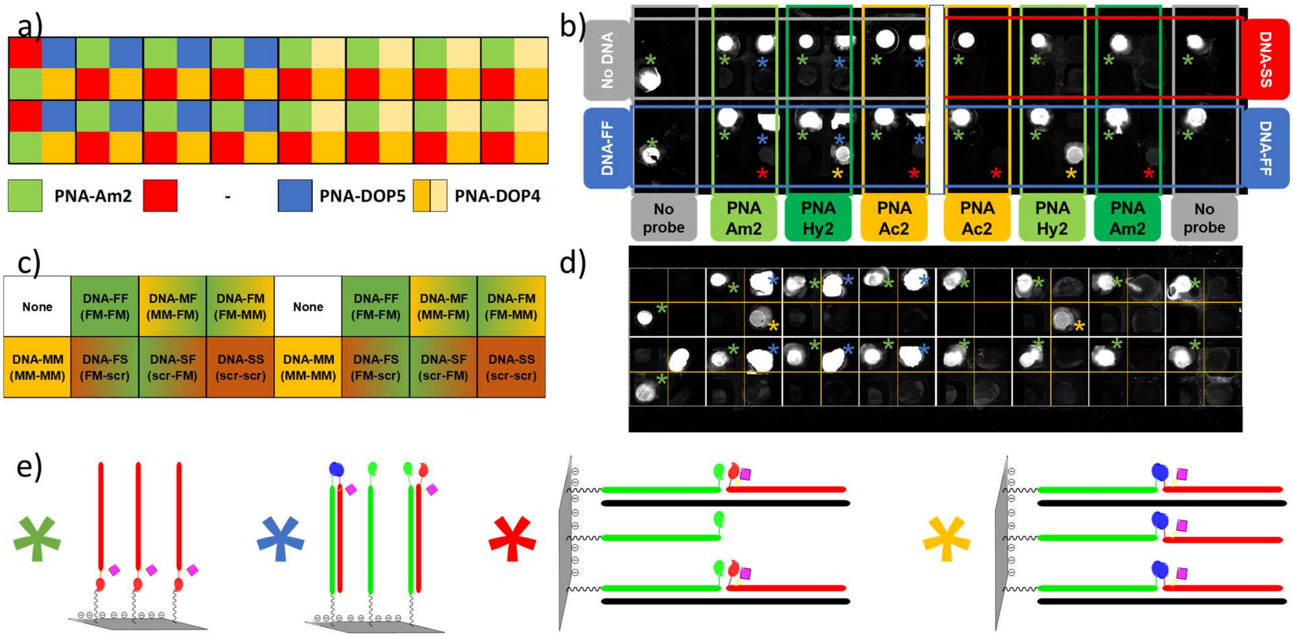

Figure S25: templated ligation on microarray surfaces: (a) scheme of the probes spotted on the surface; (b) picture of the microarray slide after weak washes, with indication of the probes loaded before incubation; (c) scheme of the DNA loaded for the evaluation of **PNA-Hy2** selectivity and (d) picture of the relative microarray slide after wash; (e) graphical explanation of the surface architecture for: positive control (green star), strong PNA:PNA hybridization that does not allow to distinguish between non- and ligated probes (blue star), non-ligated PNA_2:DNA complex (red star), and ligated PNA-PNA:DNA complex (orange star).

In brief, the microarray slide was divided into two distinct regions (as depicted in Figure S25a), to test the possibility to discriminate ligation in the PNA:PNA duplex (left side, blue squares) or the effect of PNA concentration during surface functionalization (right side, light yellow square indicates 200 nM PNA solution for spotting). The surface image was acquired after stringent washing conditions to remove unbound probes and discriminate between stable PNA_2:DNA complexes (red stars) and the PNA-PNA ligation products (orange stars). In the first experiment (Figure S25b) the chemoselectivity of the reaction among the different nucleophiles is shown. In a second experiment (Figure S25d) the selectivity of the surface ligation in presence of different DNA templating strands (Figure S25c) is shown.

7. Proximity induced peptide ligation

In a typical experiment, 100 µL of buffered solution (PBS pH 7.4) containing probes at 5 µM concentration (from a 100 µM stock solution), were prepared in a 0.5 mL Eppendorf and allowed to react overnight at 25 °C. The solutions were collected in the morning and analyzed via HPLC-UV, HPLC-MS.

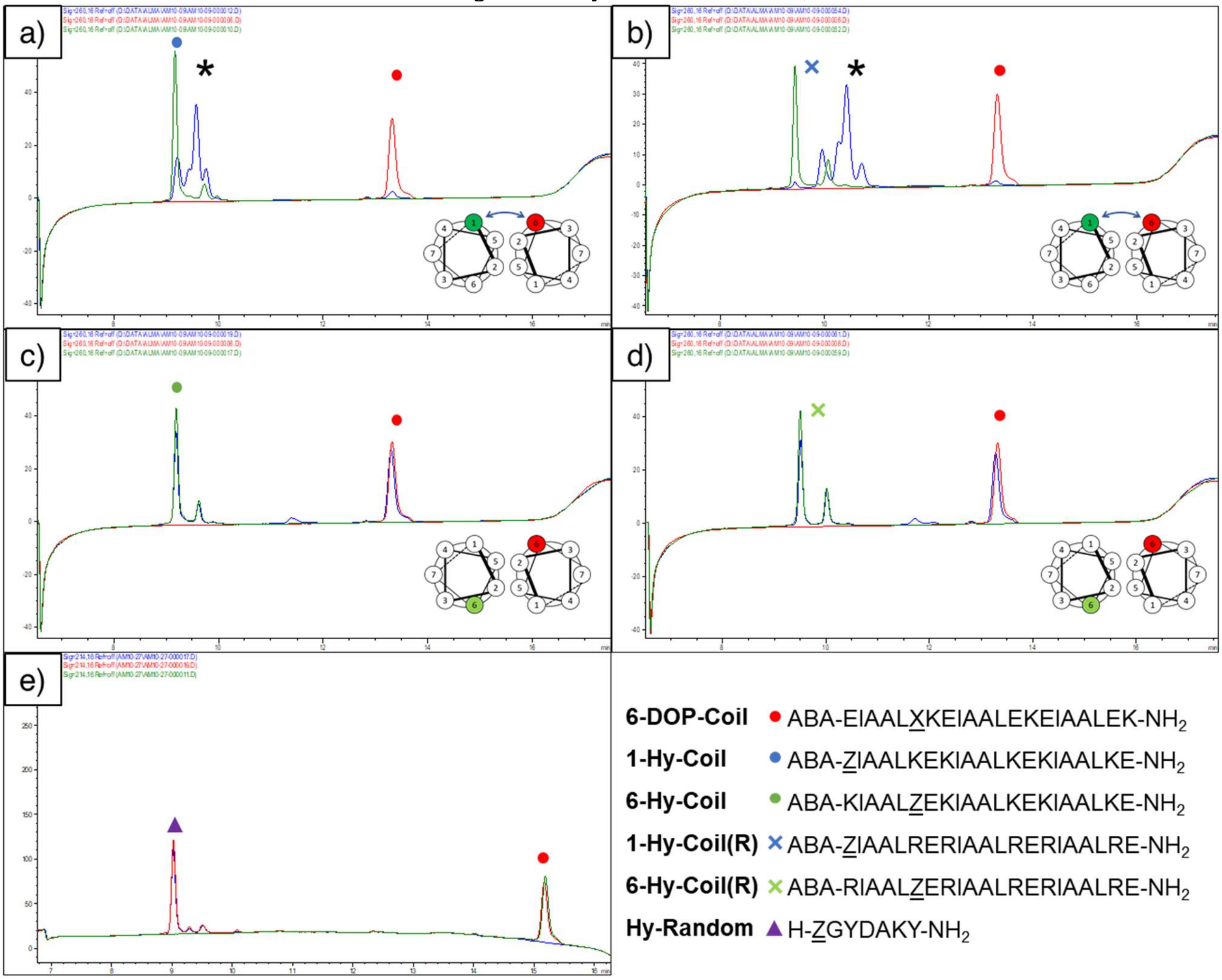

Figure S26: HPLC4 traces of the ligation experiments performed in presence of **6-DOP-Coil.** (a) with **1-Hy-Coil**, (b) with **1-Hy-Coil(R)**, (c) with **6-Hy-Coil**, (d) with **6-Hy-Coil(R)**, (e) with **Hy-Random**. X: DOP-modified ornithine; Z: hydrazine-modified ornithine; *: ligation product. In panels (c) and (d) a traces of unselective ligation (< 5%) are present at Rt 11.5 minutes; no unselective ligation was observed in panel (e).

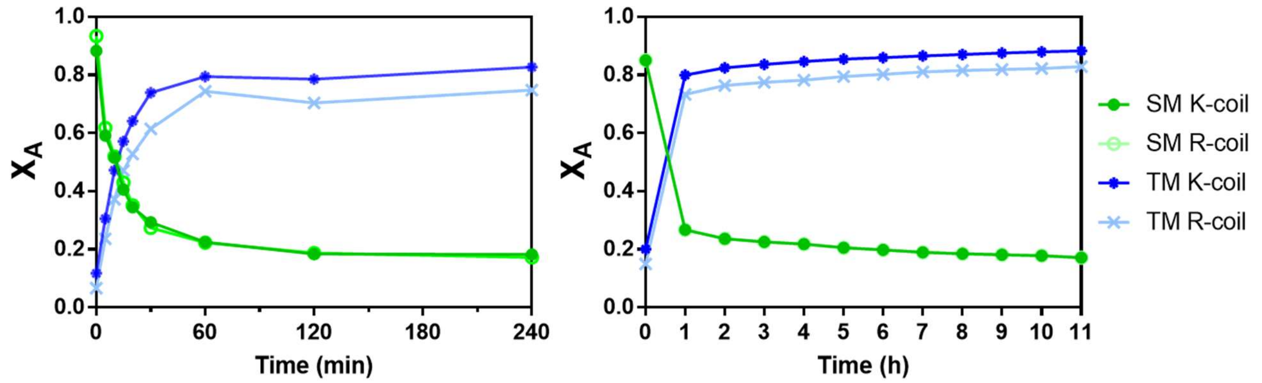

Figure S27: reaction kinetics in coiled coils systems. Ligation experiments performed with **1-Hy-Coil** (or **1-Hy-Coil(R)**) in presence of 1.1 eq of **6-DOP-Coil** at different time points.

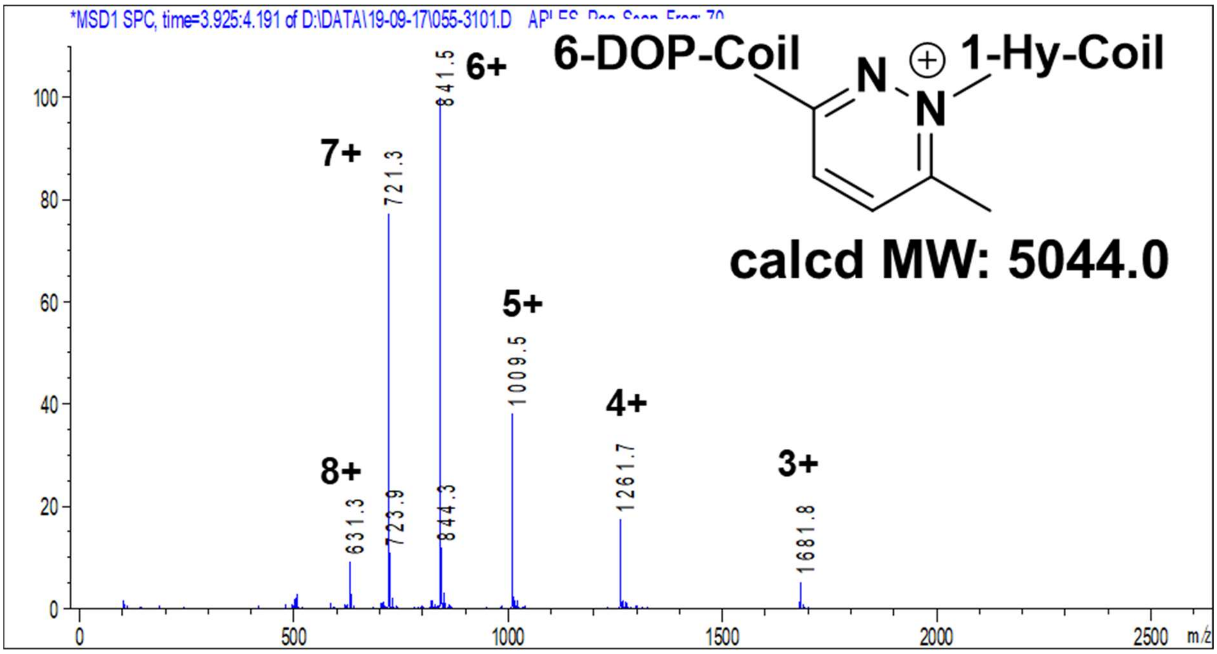

Figure S28: ESI-MS characterization of the product formed between **6-DOP-Coil** and **1-Hy-Coil**.

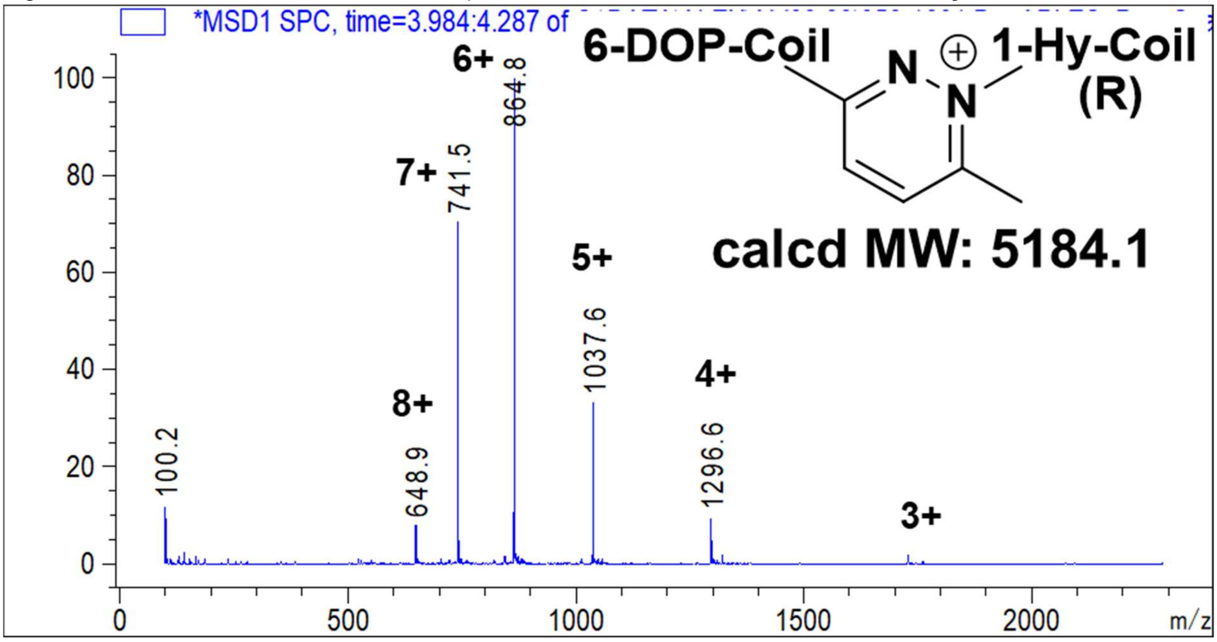

Figure S29: ESI-MS characterization of the product formed between **6-DOP-Coil** and **1-Hy-Coil(R)**.

8. Supplementary References

(1) Manicardi, A.; Cadoni, E.; Madder, A. Visible-Light Triggered Templated Ligation on Surface Using Furan-Modified PNAs. *Chem. Sci.* **2020**, *11* (43), 11729–11739. https://doi.org/10.1039/D0SC04875E.

(2) Díaz-Mochón, J. J.; Bialy, L.; Bradley, M. Full Orthogonality between Dde and Fmoc: The Direct Synthesis of PNA-Peptide Conjugates. *Org. Lett.* **2004**, *6* (7), 1127–1129. https://doi.org/10.1021/ol049905y.

(3) Chouikhi, D.; Ciobanu, M.; Zambaldo, C.; Duplan, V.; Barluenga, S.; Winssinger, N. Expanding the Scope of PNA-Encoded Synthesis (PES): Mtt-Protected PNA Fully Orthogonal to Fmoc Chemistry and a Broad Array of Robust Diversity-Generating Reactions. *Chem. - A Eur. J.* **2012**, *18* (40), 12698–12704. https://doi.org/10.1002/chem.201201337.

(4) Haack, R. A.; Swift, K. M.; Ruan, Q.; Himmelsbach, R. J.; Tetin, S. Y. The Photostability of the Commonly Used Biotin-4-Fluorescein Probe. *Anal. Biochem.* **2017**, *531*, 78–82. https://doi.org/10.1016/J.AB.2017.05.019.

9. HPLC-MS chromatograms of pure PNAs and peptides

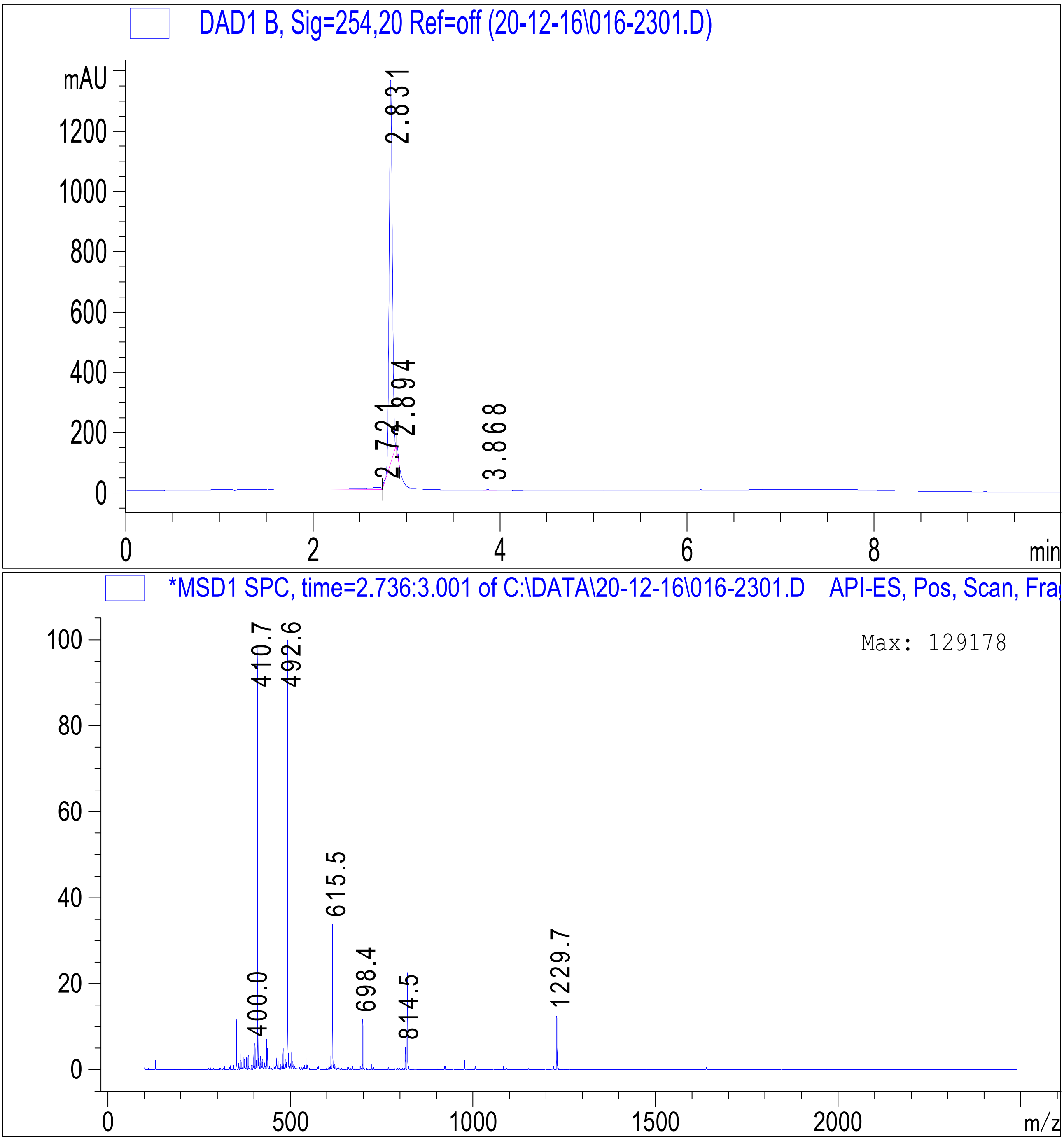

Figure S30: HPLC-MS chromatogram of purified **PNA-Hy1'**. HPLC-UV trace at 254 nm (top) and MS spectrum of the corresponding peak (bottom). Calcd MW: 2458.5.

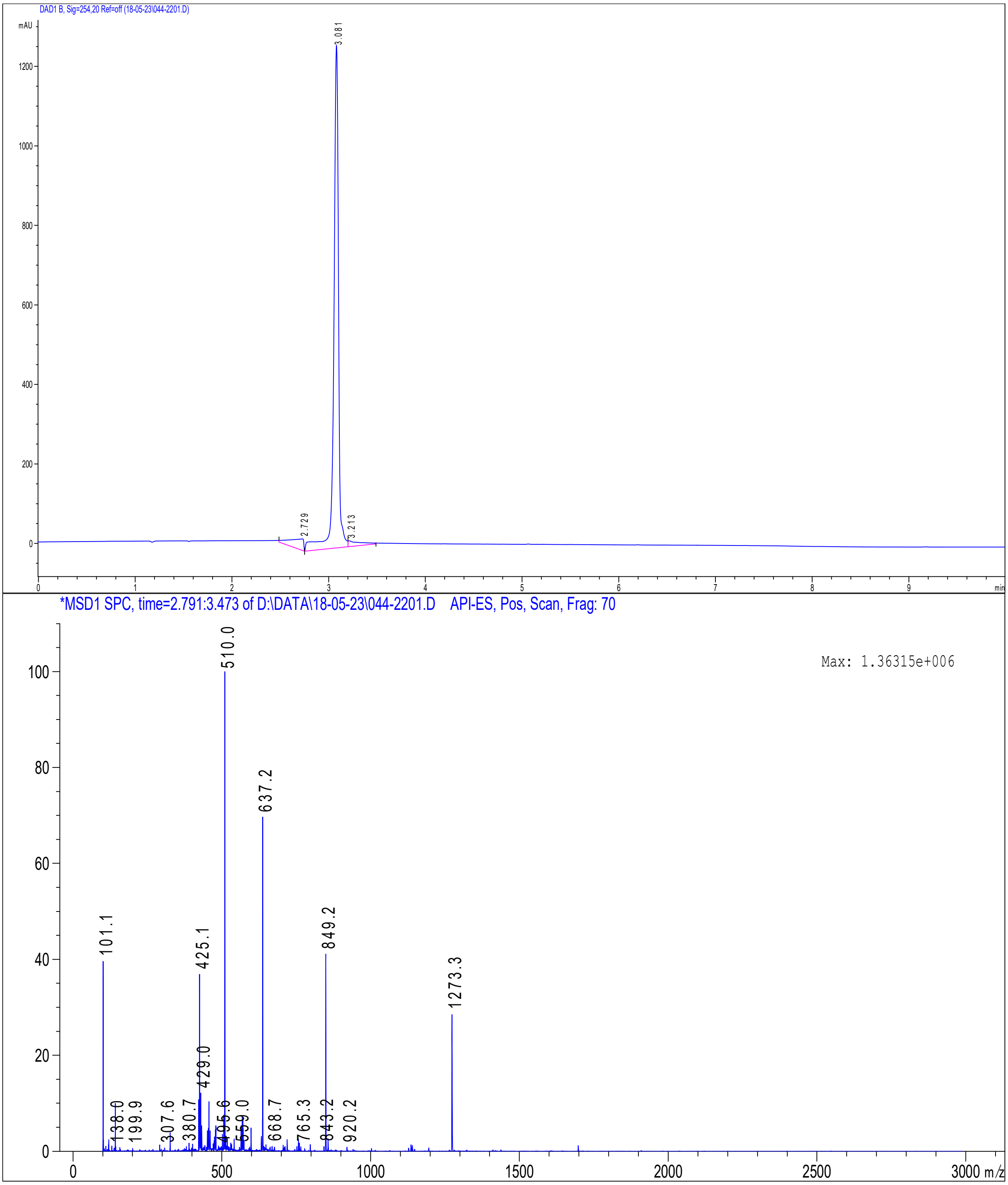

Figure S31: HPLC-MS chromatogram of purified **PNA-DOP1**. HPLC-UV trace at 254 nm (top) and MS spectrum of the corresponding peak (bottom). Calcd MW: 2545.6.

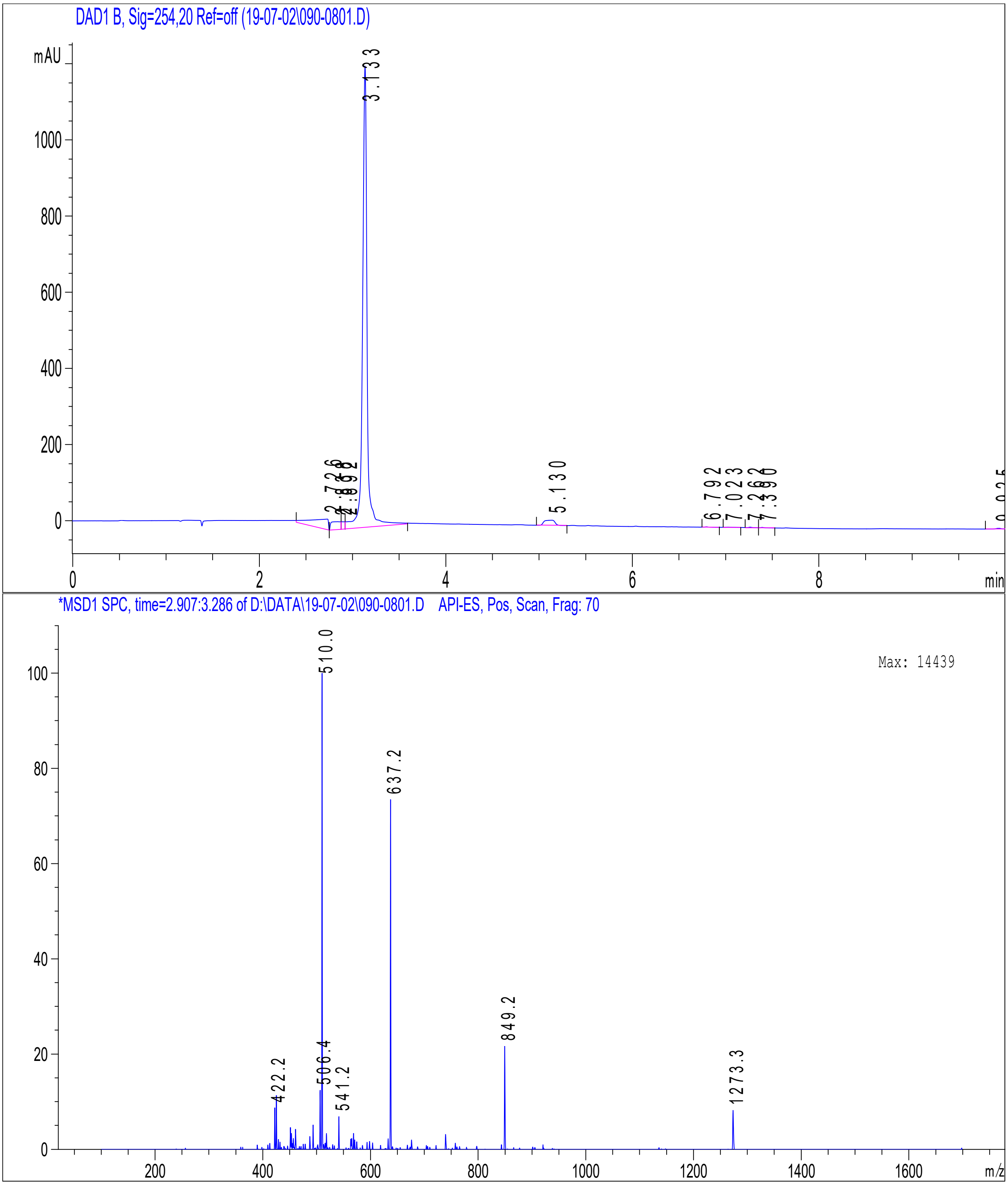

Figure S32: HPLC-MS chromatogram of purified **PNA-DOP2**. HPLC-UV trace at 254 nm (top) and MS spectrum of the corresponding peak (bottom). Calcd MW: 2545.6.

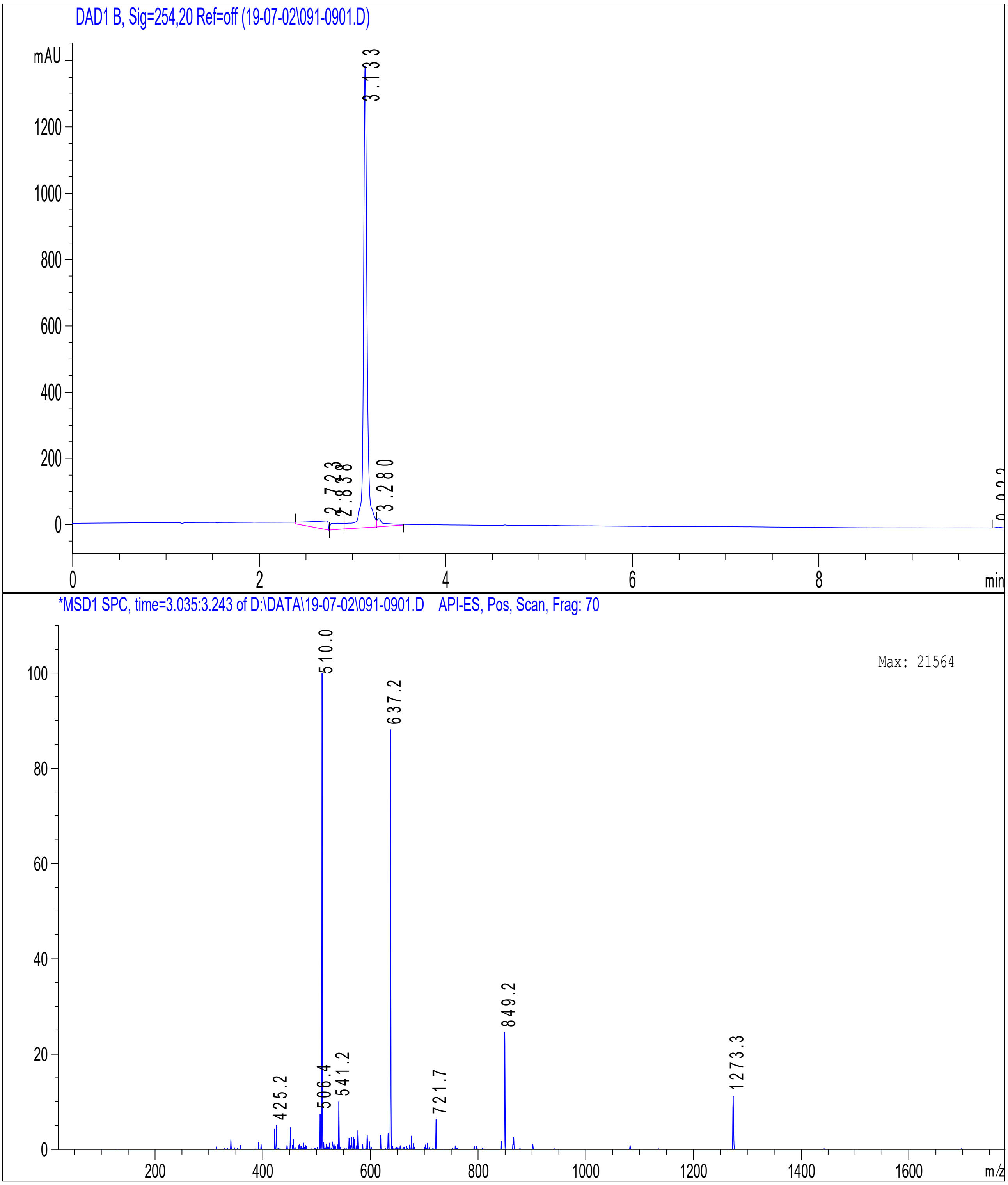

Figure S33: HPLC-MS chromatogram of purified **PNA-DOP3**. HPLC-UV trace at 254 nm (top) and MS spectrum of the corresponding peak (bottom). Calcd MW: 2545.6.

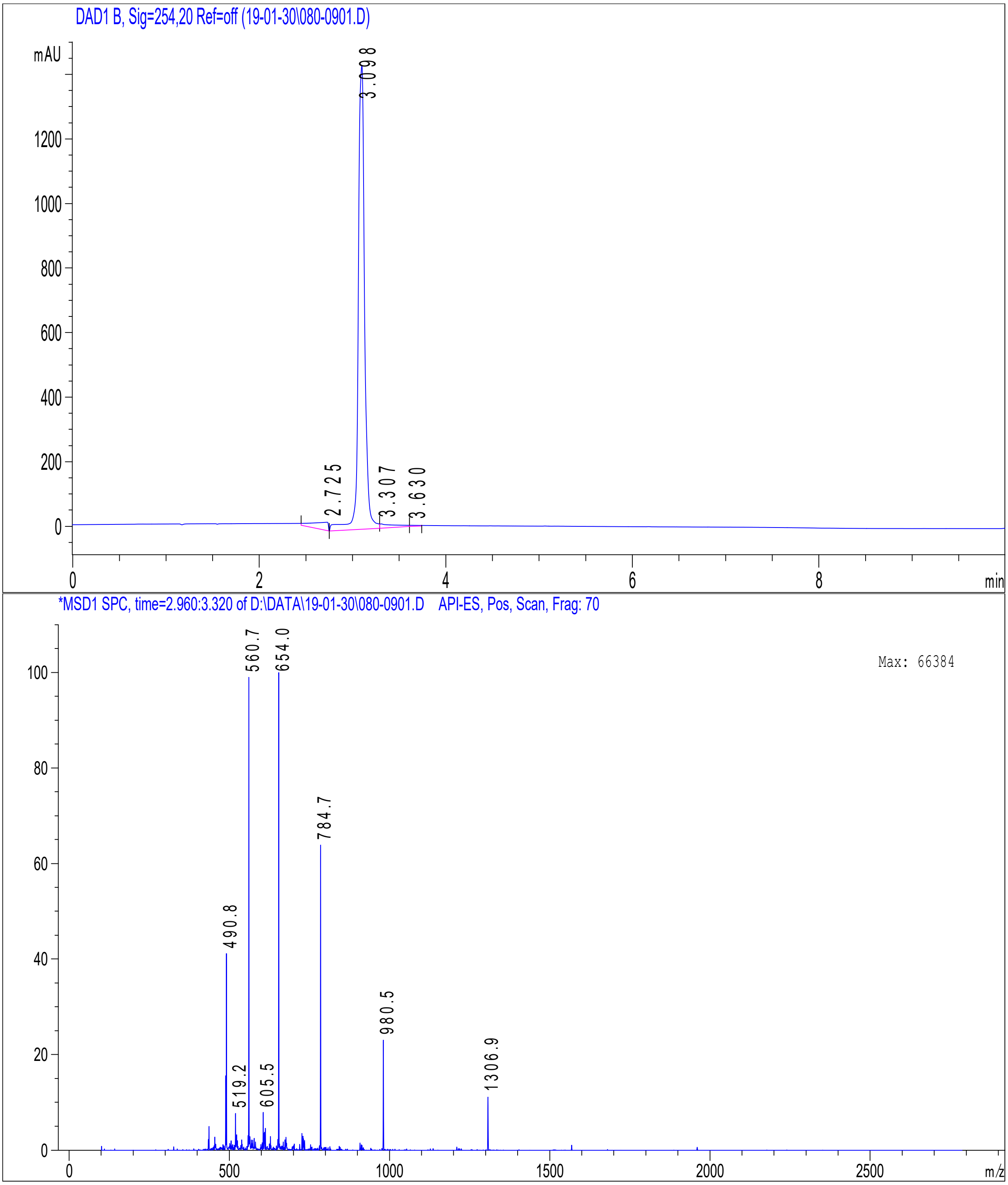

Figure S34: HPLC-MS chromatogram of purified **PNA-DOP4**. HPLC-UV trace at 254 nm (top) and MS spectrum of the corresponding peak (bottom). Calcd MW: 3919.0.

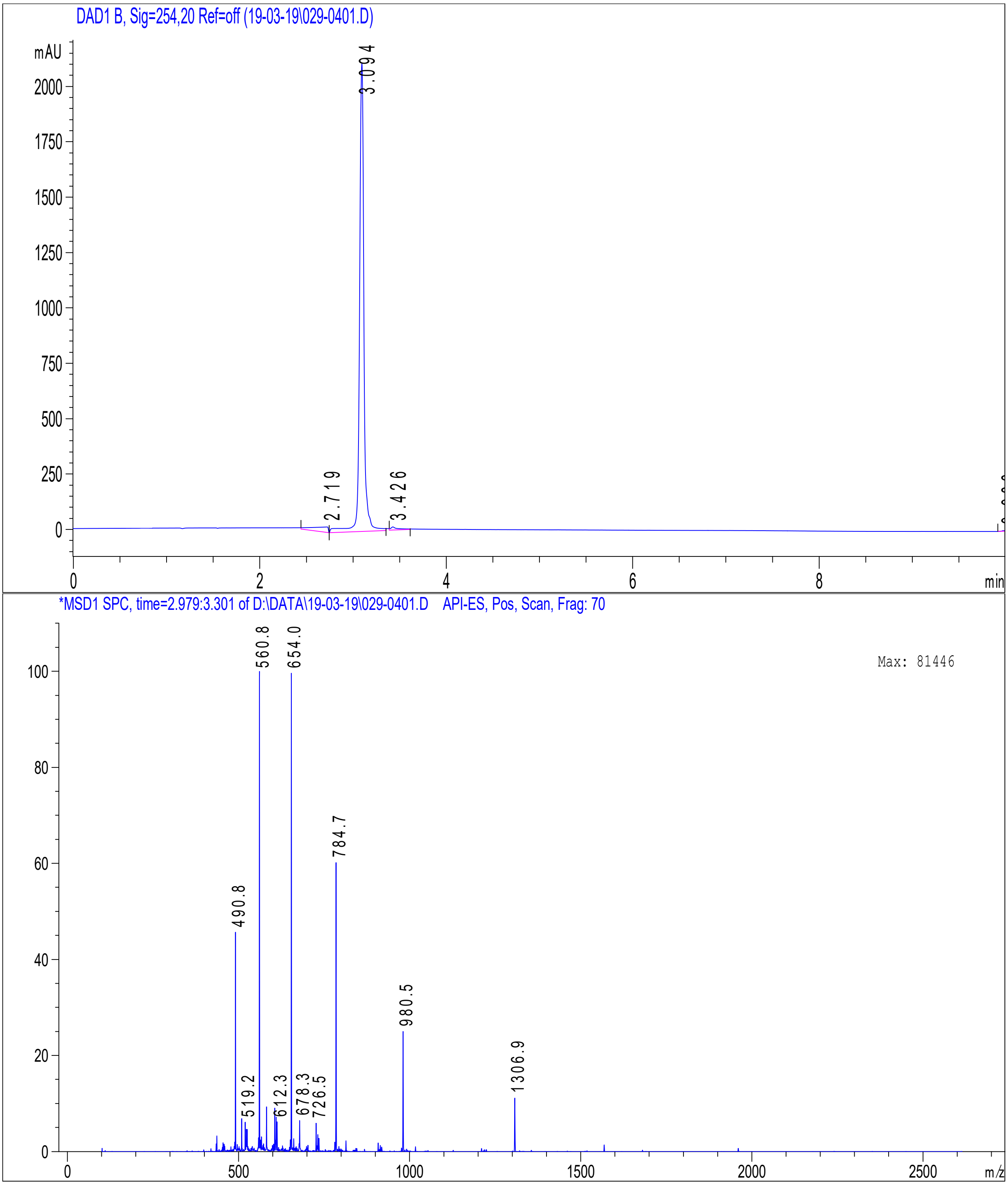

Figure S35: HPLC-MS chromatogram of purified **PNA-DOP5**. HPLC-UV trace at 254 nm (top) and MS spectrum of the corresponding peak (bottom). Calcd MW: 3919.0.

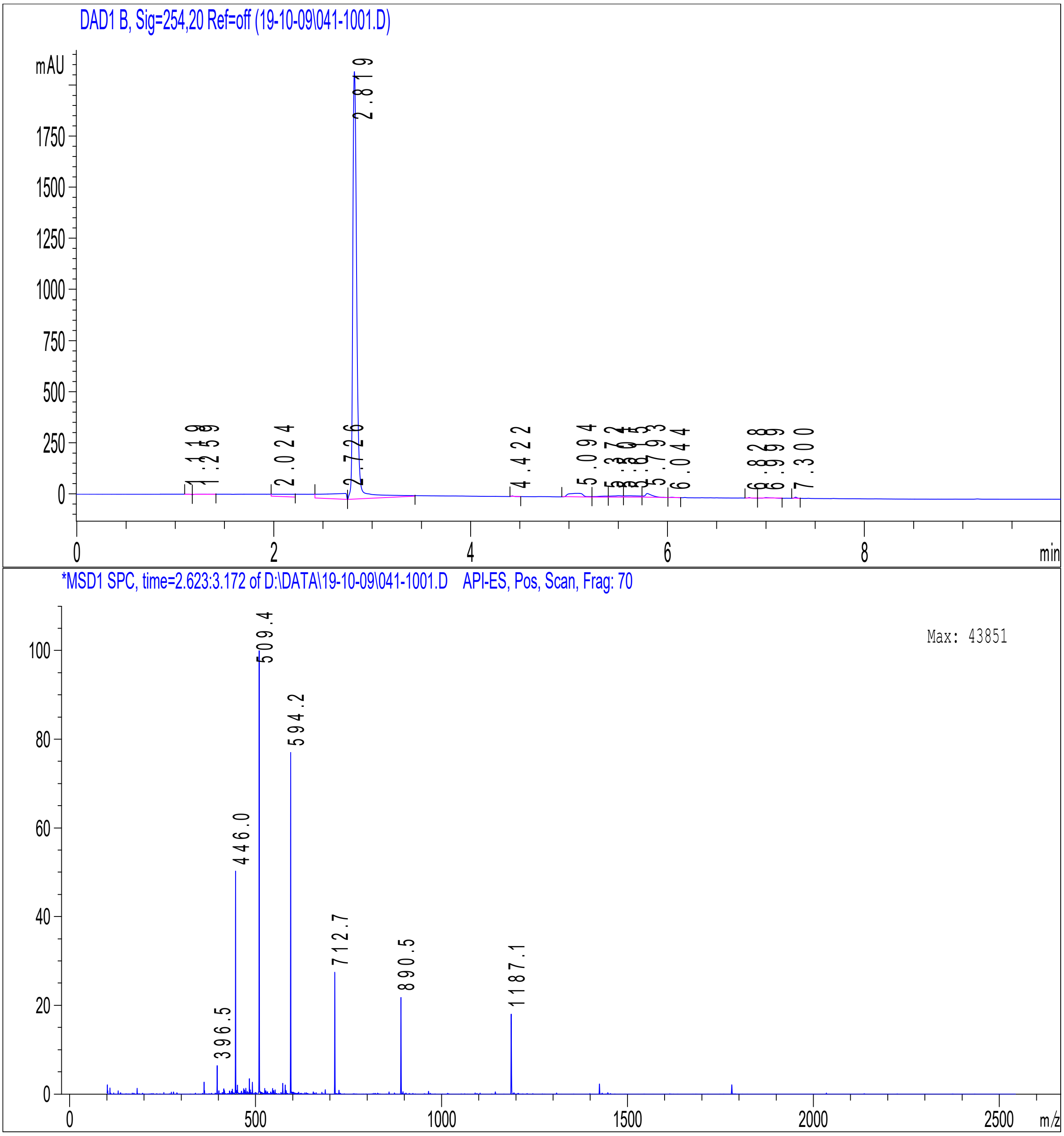

Figure S36: HPLC-MS chromatogram of purified **PNA-K1**. HPLC-UV trace at 254 nm (top) and MS spectrum of the corresponding peak (bottom). Calcd MW: 3559.6.

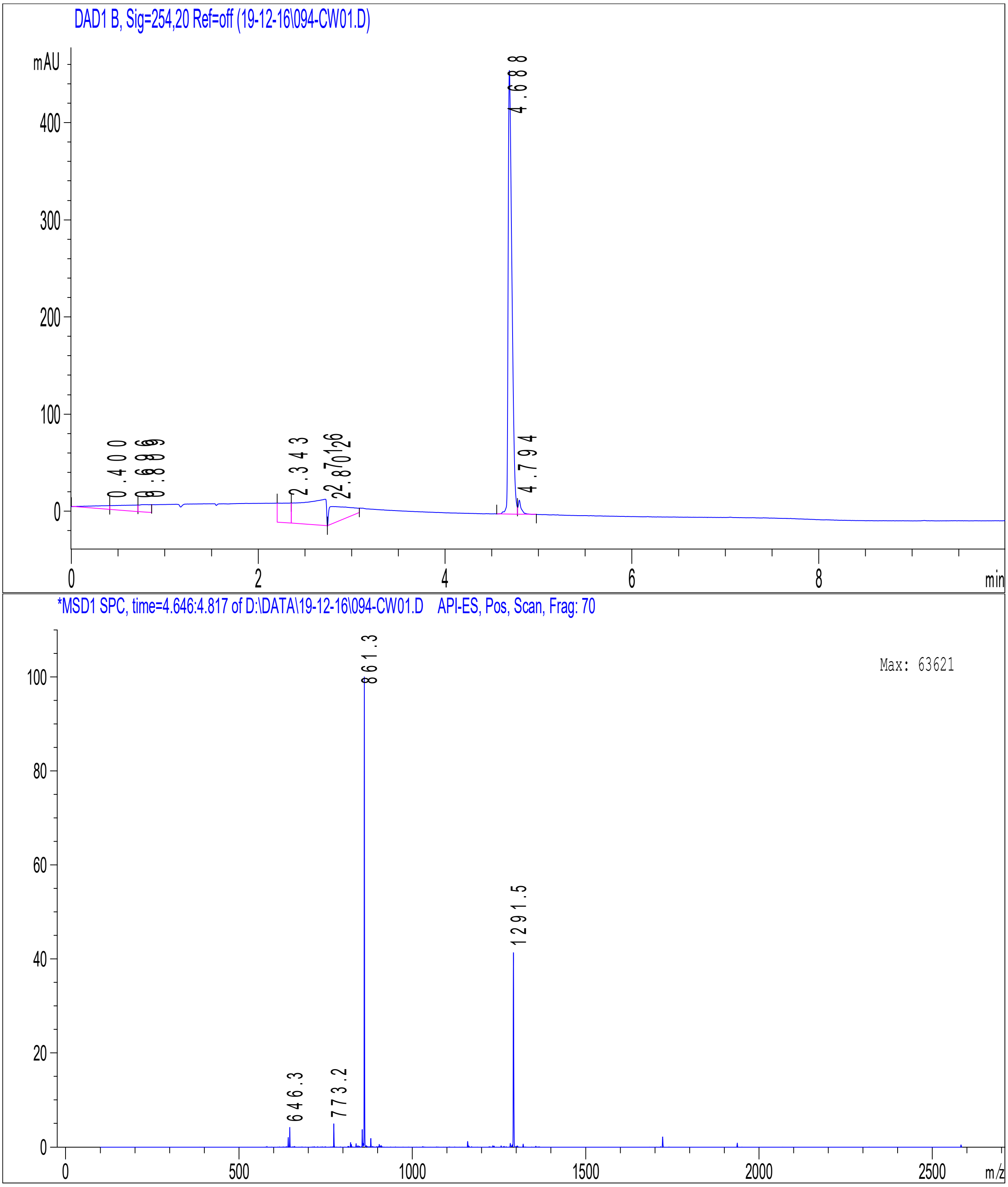

Figure S37: HPLC-MS chromatogram of purified **6-DOP-Coil**. HPLC-UV trace at 254 nm (top) and MS spectrum of the corresponding peak (bottom). Calcd MW: 2582.9.

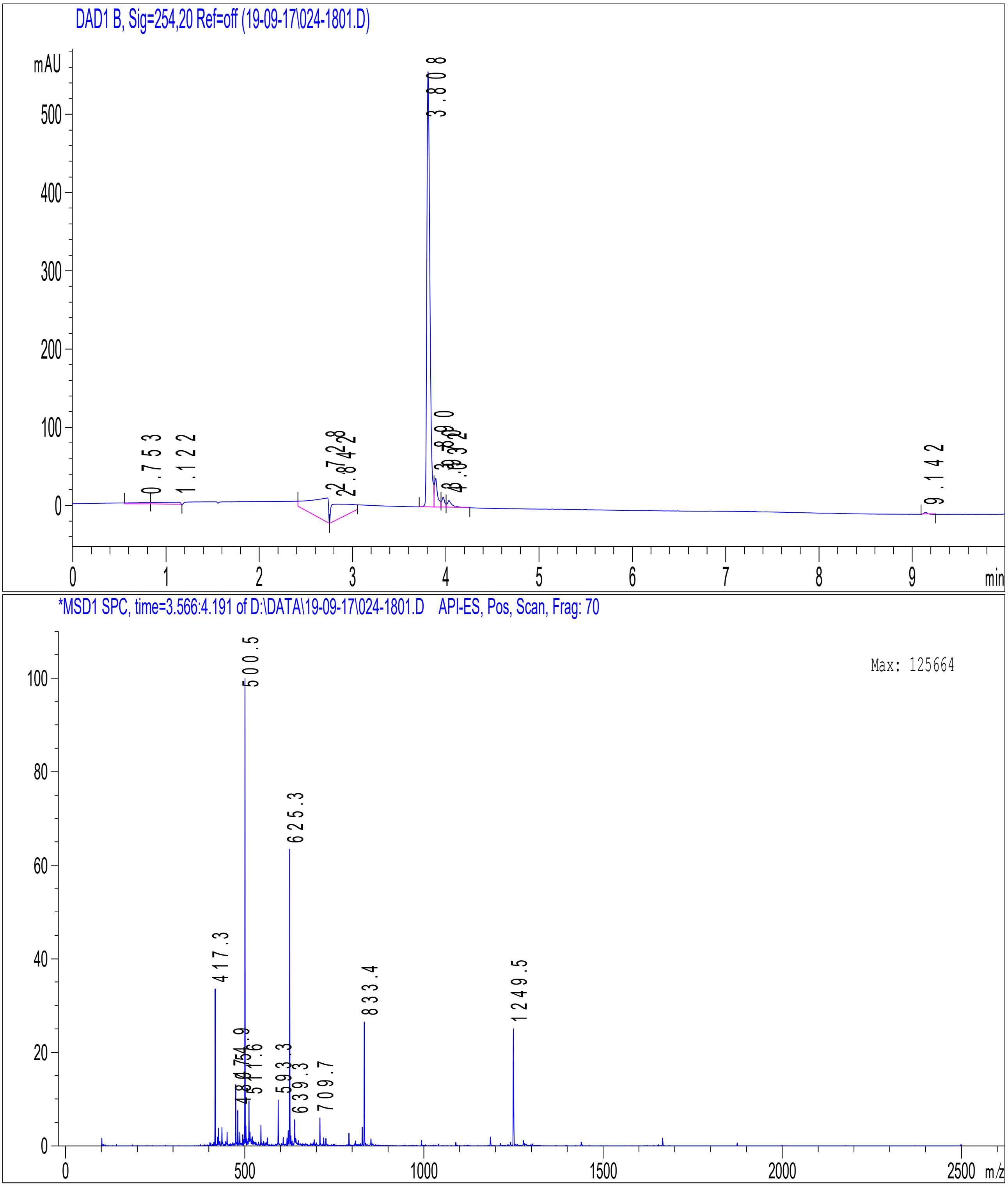

Figure S38: HPLC-MS chromatogram of purified **1-Hy-Coil**. HPLC-UV trace at 254 nm (top) and MS spectrum of the corresponding peak (bottom). Calcd MW: 2425.9.

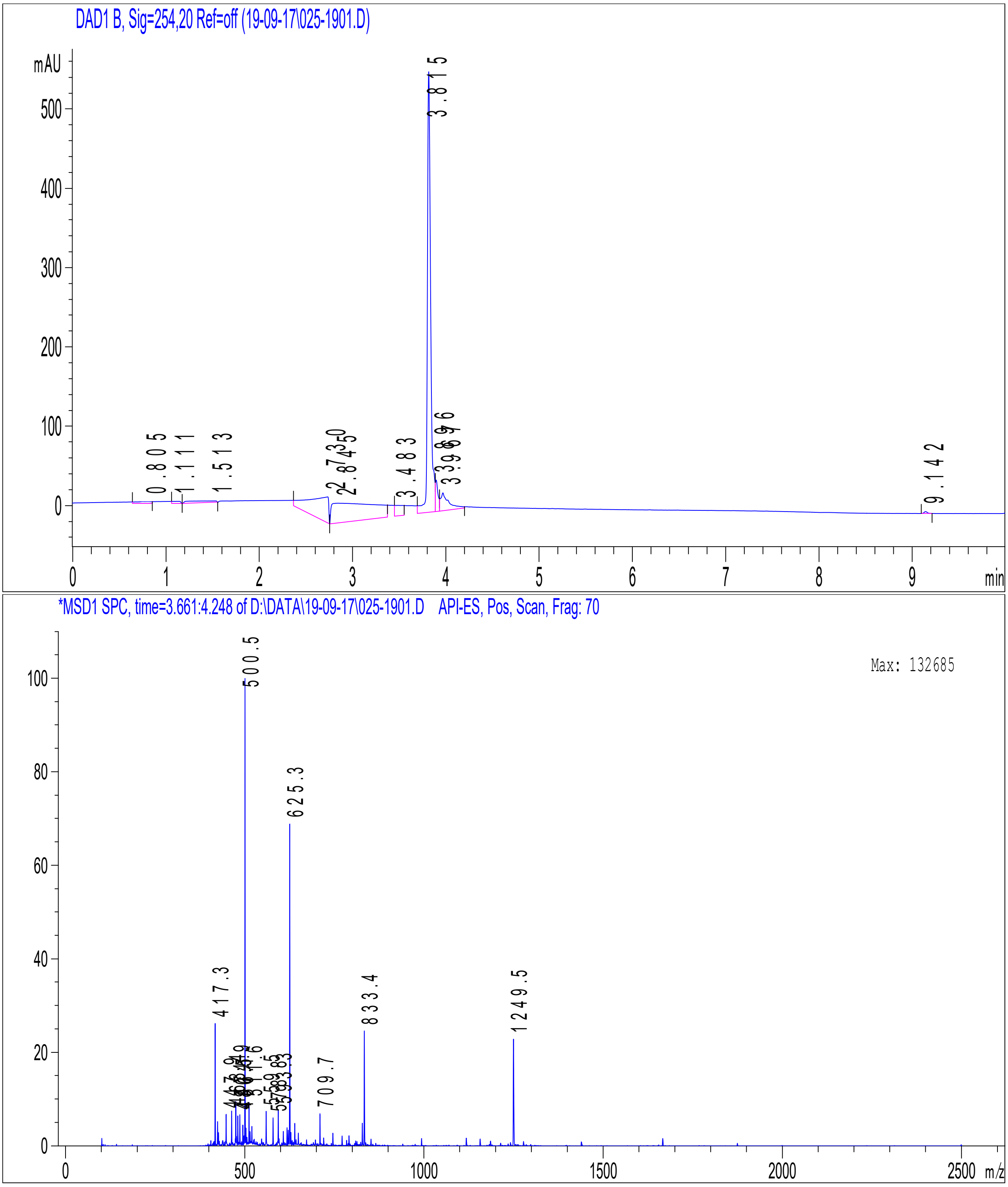

Figure S39: HPLC-MS chromatogram of purified **6-Hy-Coil**. HPLC-UV trace at 254 nm (top) and MS spectrum of the corresponding peak (bottom). Calcd MW: 2425.9.

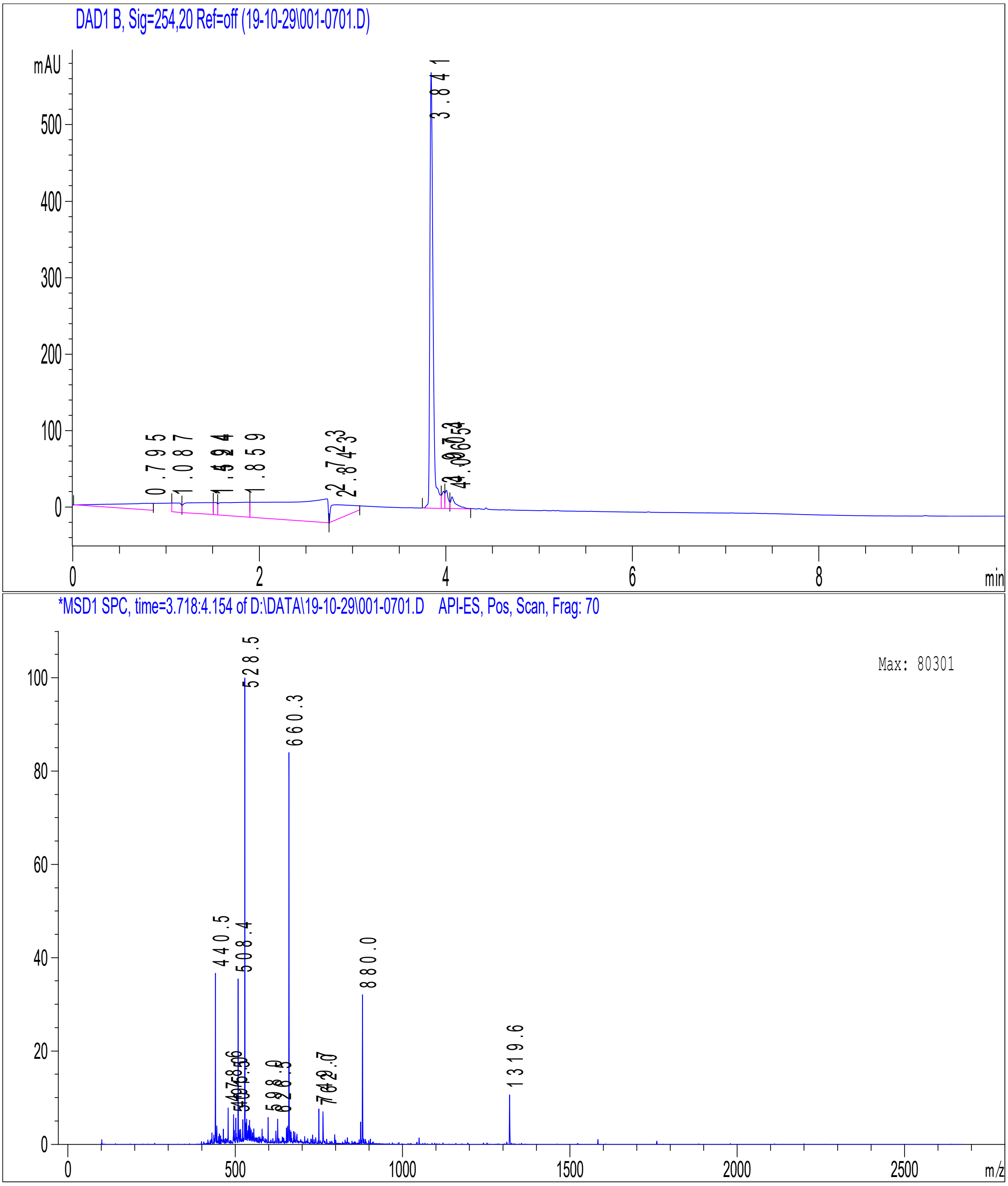

Figure S40: HPLC-MS chromatogram of purified **1-Hy-Coil(R)**. HPLC-UV trace at 254 nm (top) and MS spectrum of the corresponding peak (bottom). Calcd MW: 2638.1.

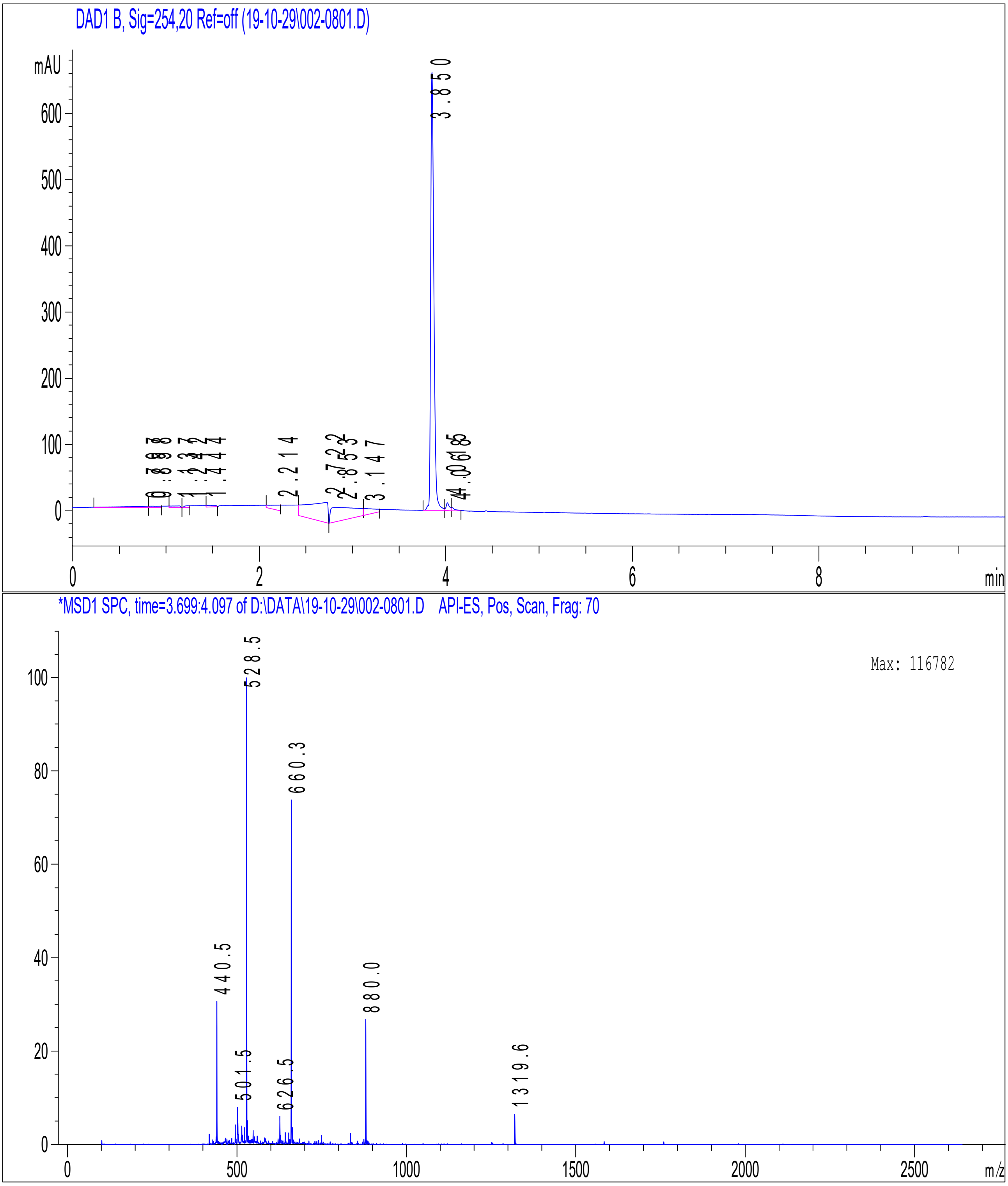

Figure S41: HPLC-MS chromatogram of purified **6-Hy-Coil(R)**. HPLC-UV trace at 254 nm (top) and MS spectrum of the corresponding peak (bottom). Calcd MW: 2638.1.

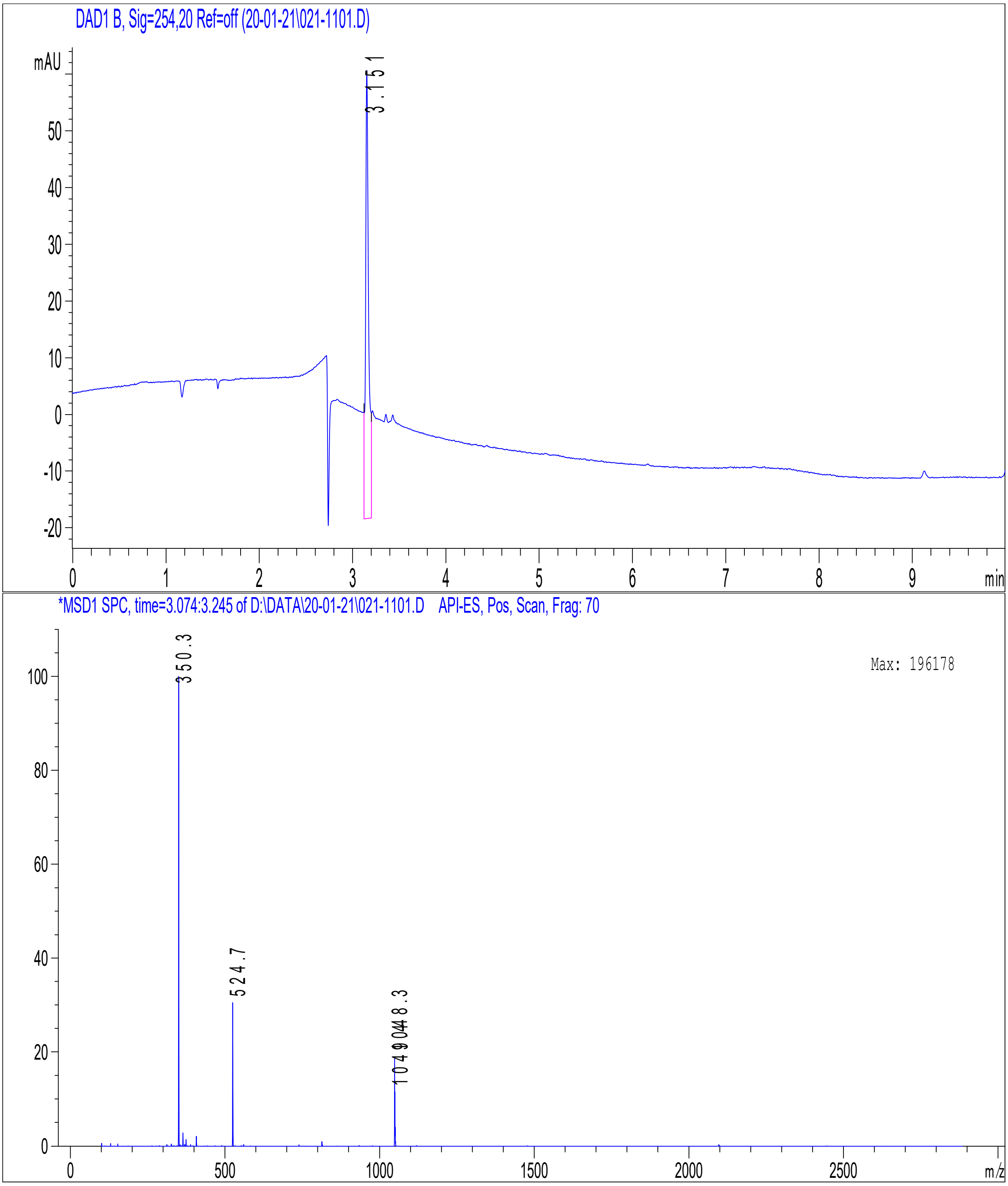

Figure S42: HPLC-MS chromatogram of purified **Hy-random**. HPLC-UV trace at 254 nm (top) and MS spectrum of the corresponding peak (bottom). Calcd MW: 1048.2.
